# Integrating inflammatory and coagulation biomarkers for surgical risk stratification and treatment benefit assessment in Crohn’s disease

**DOI:** 10.3389/fimmu.2026.1657279

**Published:** 2026-05-07

**Authors:** Kailing Xie, Qi Sun, Lichao Yang, Zhixian Jiang, Hao Liu, Yawei Zhang, Hengchang Yao, Qiang Wu, Baojia Yao, Liangxin Peng, Dan Zhang, Lianwen Yuan

**Affiliations:** 1 Department of General Surgery, The Second Xiangya Hospital, Central South University, Changsha, Hunan, China; 2 Department of Geriatric Surgery, The Second Xiangya Hospital, Central South University, Changsha Hunan, China; 3 Clinical Nursing Teaching and Research Section, The Second Xiangya Hospital, Central South University, Changsha, Hunan, China

**Keywords:** biologic therapy, coagulation biomarkers, Crohn’s disease, inflammatory biomarkers, machine learning, surgical risk stratification

## Abstract

**Introduction:**

Inflammatory and coagulation abnormalities are closely linked to disease progression in Crohn’s disease (CD). However, whether integrating these biomarkers can improve long-term surgical risk stratification and inform treatment decision-making remains unclear.

**Methods:**

A total of 1,060 patients with CD were enrolled. Candidate predictors, including inflammatory and coagulation biomarkers, were selected using LASSO-Cox regression and the Boruta algorithm. Surgery-free survival was assessed using Kaplan-Meier analysis. Eight machine learning models were developed and evaluated using five-fold cross-validation. Shapley additive explanations (SHAP) were used to interpret the best-performing model. Inverse probability weighting was applied to reduce confounding and selection bias and to assess the benefit of biologic therapy across risk strata.

**Results:**

Inflammatory and coagulation biomarkers, together with disease-related features, were major determinants of long-term surgical risk in CD. Among the eight models tested, the gradient boosting machine (GBM) achieved the best performance, with a C-index of 0.816 (95% confidence interval [CI], 0.789-0.843), significantly outperforming the Cox model (0.680, 95% CI, 0.643-0.717; *P* < 0.001). The model showed robust time-dependent discrimination, with 1-, 3-, 5-AUC values up to 0.836, 0.851, 0.832, and an integrated Brier score of 0.103. SHAP analysis indicated that inflammatory and coagulation markers together contributed approximately 70% of GBM model explainability. Consistent with these findings, dual-mediator analysis showed that fibrinogen accounted for 44.5% of the inflammation-associated increase in surgical risk, whereas D-dimer mediated 6% of the excess risk. Compared with the Cox model, the GBM improved 5-year risk reclassification, with a net reclassification improvement of 0.425 and an integrated discrimination improvement of 0.181. After inverse probability weighting, biologic therapy was associated with significant benefit in the intermediate- (pooled hazard ratio (HR) = 0.44, 95% CI: 0.26-0.75, pooled *P* = 0.003) and high-risk groups (pooled HR = 0.51, 95% CI: 0.30-0.88, pooled *P* = 0.016), but not in the low-risk group (pooled HR = 0.87, 95% CI: 0.47-1.61, pooled *P* = 0.657). An online platform was developed to support individualized risk stratification and treatment assessment.

**Conclusion:**

Integrating inflammatory and coagulation biomarkers improves surgical risk stratification in Crohn’s disease and may help identify patients most likely to benefit from biologic therapy.

## Graphical Abstract

**Figure:**
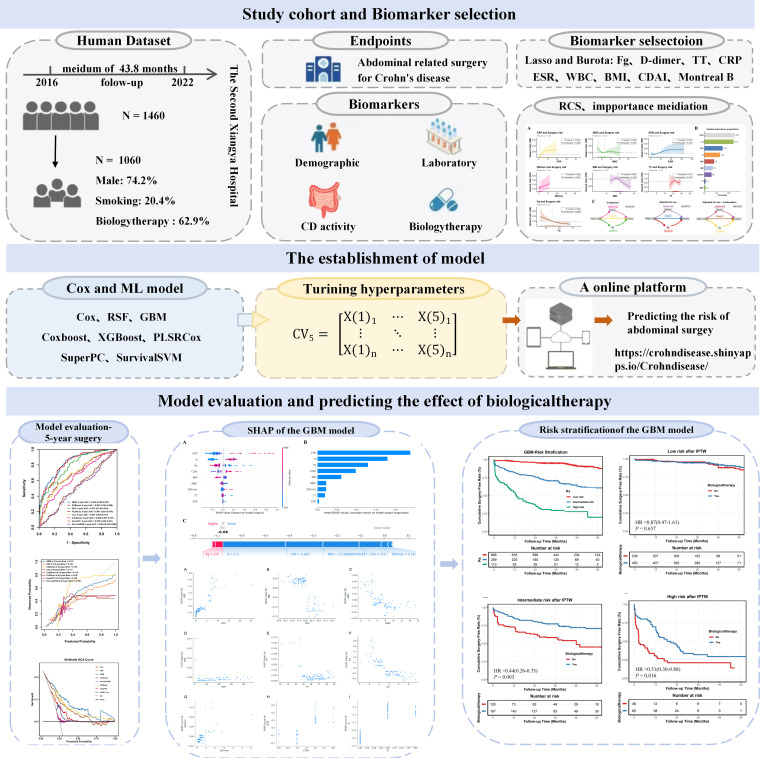

## 1 Introduction

Crohn’s disease (CD) is a chronic, progressive inflammatory bowel disease characterized by relapsing intestinal inflammation and cumulative bowel damage (1, 2). Despite substantial advances in medical therapy, particularly the widespread use of biologics, a considerable proportion of patients with CD still eventually require abdominal surgery because of strictures, fistulas, abscesses, or other irreversible structural complications (2, 3). Identifying patients at high risk of surgical progression therefore remains a major unmet clinical need, especially in the context of long-term disease management (4–6).

Persistent inflammation is a key driver of transmural injury and subsequent bowel damage in CD (1, 2). In parallel, coagulation abnormalities and a prothrombotic state have increasingly been linked to inflammatory activity and adverse clinical outcomes in inflammatory bowel disease, suggesting that coagulation-related biomarkers may provide important insight into disease progression in Crohn’s disease (2, 7–10). Because these markers are readily available in routine practice, they may offer a practical basis for long-term risk stratification and treatment guidance, particularly for identifying patients who are more likely to benefit from earlier biologic therapy (4, 6, 11, 12).

Several studies have developed models to predict surgical risk in CD (13–17). However, most existing models either focus on short-term outcomes or rely on conventional statistical methods that may be insufficient to capture nonlinear relationships and complex interactions among predictors (13–15). In addition, although radiomics and other imaging-based approaches have shown promise in predicting disease activity and prognosis, their use in routine care remains limited by demanding data acquisition, analytical complexity, and insufficient standardization (16, 17). These limitations highlight the need for an accurate, interpretable, and clinically accessible model for long-term surgical risk prediction (6, 14, 15).

In this study, we developed and validated an interpretable machine learning model based on readily available clinical variables, with particular emphasis on inflammatory and coagulation biomarkers, to predict long-term surgical risk in CD. We further explored the contribution of these markers to risk stratification and assessed whether the resulting model could help identify patients more likely to benefit from biologic therapy.

## 2 Materials and methods

### 2.1 Study population

The study included CD patients who visited our hospital between 2016 and 2022. Inclusion criteria: (1) Patients diagnosed with CD according to the current IBD diagnosis and treatment guidelines; (2) Initial diagnosis made at Xiangya Second Hospital. Exclusion criteria: (1) Patients who had undergone abdominal or intestinal resection (except for appendectomy) prior to diagnosis or treatment at our hospital; (2) Clinical data missing more than 20%; (30 Missing follow-up data; (4) Death due to other diseases or natural causes. The primary endpoint of the study was the first abdominal surgery. Surgical indications included recurrent intestinal bleeding, acute perforation, chronic enteric fistula, recurrent intestinal obstruction, malignancy, failure of medical treatment, unclear diagnosis, and growth failure in children. This study was approved by the Ethics Committee of Xiangya Second Hospital (Ethics ID: LYEC2025-0156), and followed the Declaration of Helsinki. Patient data were anonymized throughout the study. As this was a retrospective study, informed consent was waived.

### 2.2 Baseline data and follow-up information

The baseline data for this study were extracted from the hospital’s electronic medical records and included the following variables: gender, smoking history, body mass index (BMI), white blood cell count (WBC), CRP, hemoglobin (Hb), ESR, albumin (ALB), prothrombin time (PT), activated partial thromboplastin time (APTT), Fg, D-dimer (DD), Montreal classification, history of drug use, extra-intestinal manifestations, and the CDAI at the time of diagnosis. To improve the comparability and reliability of laboratory indicators, all laboratory variables included in the analysis were extracted from the baseline assessment at the time of the patient’s first hospitalization for Crohn’s disease diagnosis, and pre-treatment test results were used whenever available. All clinical data were independently extracted by two researchers according to predefined criteria. Inter-rater agreement was assessed using the Kappa statistic, with a Kappa value of 0.85 indicating good agreement. Any discrepancies were reviewed against the original medical records and resolved by a third investigator. Information on whether patients underwent surgical treatment was gathered through telephone follow-up and inpatient electronic medical records. Follow-up visits were scheduled every 3 months, and the follow-up duration was defined as the time from the initial diagnosis to either the date of surgery or the last follow-up. Biological therapy use was defined as the administration of biologics [including infliximab (IFX), adalimumab (ADA), vedolizumab (VDZ), ustekinumab (UST), or upadacitinib (ABT-494)].

### 2.3 Statistical methods

#### 2.3.1 Imputation of missing data

Missing clinical data were handled using multiple imputation with the *mice* package, resulting in five imputed datasets. Subsequent analyses, including variable selection, model development, and validation, were conducted separately within each imputed dataset. Model parameter estimates were pooled across the five datasets according to Rubin’s rules, whereas other analysis results and reported performance measures were summarized using appropriate pooling or aggregation methods, as applicable.

#### 2.3.2 Baseline data description

The Shapiro-Wilk test was used to assess the normality of continuous variables. For continuous variables that followed a normal distribution, they were represented as mean ± standard deviation; otherwise, they were expressed as median and interquartile range (25%, 75%). For continuous variables that followed a normal distribution, independent sample t-tests were used for group comparisons; for non-normally distributed data, the Mann-Whitney U test was applied. Categorical variables were represented as frequency and percentage. For sample sizes larger than 40, if the expected value was greater than 5, the Pearson chi-square test was used; if the expected value was between 1 and 5, Continuity-corrected Chi-square was applied; when the sample size was smaller than 40 or the expected value was less than 1, Fisher’s exact test was used.

#### 2.3.3 Survival analysis and assessment of factors associated with surgical risk

Kaplan–Meier curves were used to estimate the cumulative non-surgical rate, and differences between groups were compared using the log-rank test. In addition, Cox proportional hazards regression was performed to evaluate the associations between candidate variables and surgical risk, with hazard ratios (HRs) and 95% confidence intervals (CIs) reported. Following multiple imputation, all analyses were conducted separately within each of the five imputed datasets, and the regression estimates were pooled according to Rubin’s rules. Furthermore, the relative importance of each variable in the Cox model was quantified by its proportion of adjusted Wald chi-square, calculated as (Wald χ² - df)/Σ (Wald χ² - df) (18, 19). To further explore potential dose–response and nonlinear associations between continuous variables and surgical risk, restricted cubic splines (RCS) with four knots placed at the 5th, 35th, 65th, and 95th percentiles were incorporated into the multivariable Cox regression models. The overall and nonlinear effects were estimated separately in each imputed dataset and then pooled across imputations to obtain pooled *P* for overall and pooled *P* for non-linearity. The final RCS curves were plotted on the basis of the pooled spline coefficients. Meanwhile, multicollinearity among variables was assessed using Pearson correlation coefficients, with an absolute correlation coefficient >0.7 considered indicative of substantial multicollinearity.

#### 2.3.4 Stabilized inverse probability of treatment weighting

To reduce baseline confounding, stabilized IPTW was performed based on propensity scores estimated from a multivariable logistic regression model. The model included demographic factors (sex and smoking status), laboratory indicators (BMI, WBC, Hb, ESR, CRP, TT, APTT, PT, Fg, D-dimer, and ALB), disease activity and Montreal classification variables (CDAI; age at diagnosis, disease location, and disease behavior), and clinical manifestations (extraintestinal symptoms, oral ulcer, hepatobiliary involvement, skin involvement, eye involvement, osteoarthritis, and perianal abscess). Stabilized weights were then calculated according to treatment status: for patients receiving biological therapy, the weight was defined as.


$$SW_{i}=\frac{P(T=1)}{PS_{i}}$$

and for those not receiving biological therapy, the weight was defined as.


$$SW_{i}=\frac{P(T=0)}{1-PS_{i}}$$

where P(T = 1) and P(T = 0) represent the marginal probabilities of receiving and not receiving biological therapy, respectively, and PS_i_ denotes the propensity score for the i-th individual. The weighted cohort was used to improve baseline covariate balance between groups and to reduce confounding bias in subsequent analyses.

#### 2.3.5 ML survival model construction and evaluation

Predictor selection was conducted across the five multiply imputed datasets using a combined feature selection strategy. In each dataset, LASSO-Cox regression with 5-fold cross-validation and the 1-standard-error rule was used in parallel with Boruta feature selection. Predictors identified by both methods and retained in at least three of the five imputed datasets were included as candidate variables for final model development. This strategy was adopted to enhance selection stability and reduce the risk of overfitting.

Eight survival models were developed using the selected predictors, including random survival forest (RSF), gradient boosting machine (GBM), survival support vector machine (SurvivalSVM), XGBoost, supervised principal components (SuperPC), partial least squares regression for Cox models (PLSR-Cox), and CoxBoost, with the conventional Cox model included as a benchmark. For each machine learning model, hyperparameters were tuned separately within each imputed dataset using 5-fold cross-validation. Briefly, the mean C-index was evaluated across candidate hyperparameter combinations within each imputed dataset, and the best-performing combinations identified in the individual datasets were then compared across all five imputations. The final hyperparameter setting for each algorithm was selected according to the mean cross-validated C-index aggregated across the five imputed datasets, with the aim of improving the stability of hyperparameter selection across imputations rather than relying on a single imputed dataset.

To improve comparability across models, the same fold assignment was used for all candidate models within each imputed dataset. In addition, continuous variables were standardized within each fold using the mean and standard deviation from the training subset only, and the corresponding validation subset was transformed using these training-derived parameters to limit information leakage during preprocessing. Model performance was assessed using out-of-fold predictions generated under the same 5-fold cross-validation framework. These predictions were used to evaluate the C-index, time-dependent receiver operating characteristic (ROC) curves and area under the curve (AUC), Brier score, calibration, and decision curve analysis (DCA). Calibration slope was evaluated under the same 5-fold cross-validation framework. Within each fold, model-derived risk scores were first mapped onto a common Cox linear predictor scale by fitting a univariable Cox model in the training portion and applying the estimated coefficient to the corresponding held-out fold. The recalibrated out-of-fold predictions were then pooled across the five folds within each imputed dataset, and a second univariable Cox model was fitted to estimate the imputation-specific cross-validated calibration slope.

To integrate results across multiple imputations, two complementary summarization strategies were applied. First, out-of-fold predicted risks were generated separately within each imputed dataset, and the predictions for the same individual were averaged across the five imputed datasets to obtain pooled individual-level predictions. The primary model performance results, including the C-index, time-dependent AUC/ROC, Brier score, calibration curves, and DCA, were then evaluated based on these pooled predictions. Second, as a sensitivity analysis, the same performance metrics, including calibration slope, were calculated separately within each imputed dataset and then summarized across imputations to assess the consistency of results obtained from the two approaches.

A single global calibration intercept was not reported because, in the Cox model, any constant term is absorbed into the baseline hazard function and is therefore not separately identifiable. Accordingly, calibration-in-the-large was treated as time-specific rather than summarized by a single global intercept.

To enhance model interpretability, SHapley Additive exPlanations (SHAP) were used to explain the machine learning models, and beeswarm plots, mean absolute SHAP summary plots, individual explanation plots, and dependence plots were generated. In addition, X-tile (20) was used to derive risk strata based on the pooled risk scores from the Cox model and the best-performing machine learning model, and reclassification improvement was quantified using the net reclassification improvement (NRI) and integrated discrimination improvement (IDI). Finally, an online calculator was developed based on the best-performing model for individualized risk prediction, with final predicted probabilities obtained by averaging the predictions from the five imputation-specific models. The machine-learning analysis code is provided in the **Supplementary Material 1**. Model development, validation, and reporting were informed by contemporary methodological principles for clinical prediction research and relevant reporting guidance (21–24).

All statistical analyses were performed using R, Python, and GraphPad Prism. A two-tailed *P*-value of<0.05 was considered statistically significant.

## 3 Results

### 3.1 Baseline characteristics

**Figure 1:**
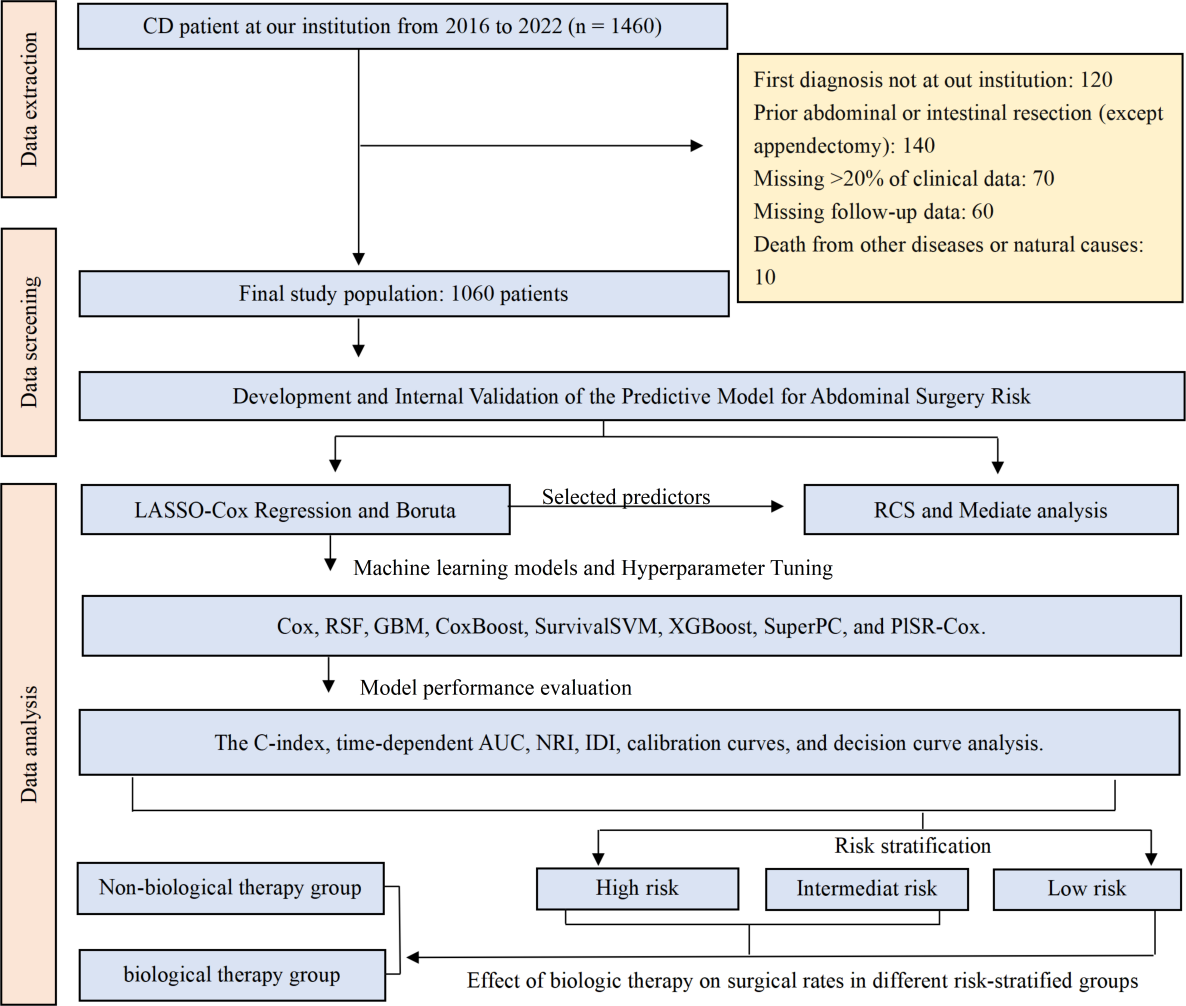
Flowchart for patient selection and data analysis.

Based on the inclusion and exclusion criteria, this research ultimately incorporated a total of 1060 participants. The flowchart presented in **Figure 1** provided a clear visual representation of the data processing steps employed in this study.

As shown in **Table 1**, the study population comprised 786 males (74.2%) and 274 females (25.8%). Among the participants, average 219 individuals (20.7%) had a prior history of smoking, while 373 cases (35.2%) were diagnosed with perianal abscesses. The distribution of patients according to the Montreal classification was as follows: in Type A, 133 (12.5%) were classified as Type 1, 788 (74.3%) as Type 2, and 139 (13.1%) as Type 3; in Type B, 473 (44.6%) were classified as Type 1, 346 (32.6%) as Type 2, and 241 (22.7%) as Type 3; in Type L, 268 (25.3%) were classified as Type 1, 67 (6.3%) as Type 2, 771 (67.1%) as Type 3, and 14 (1.3%) as Type 4. Additionally, 186 patients (17.5%) exhibited extra-intestinal manifestations, specifically: 98 patients (9.2%) had oral ulcers, 18 patients (1.7%) had liver and gallbladder lesions, 34 patients (3.2%) had skin lesions, 12 patients (1.1%) had eye involvement, and 38 patients (3.1%) had osteoarthritis. Among 1060 patients, treatment distribution was: 5-ASA (9.0%, n=95), steroids (14.3%, n=152), immunosuppressants (24.6%, n=261), and biologic agents (62.9%, n=667). The median follow-up for the entire cohort was 43.8 months. During this period, 218 patients underwent abdominal surgery, with 1-, 3-, and 5-year cumulative surgery-free survival rates of 89.3%, 82.5%, and 75.1%, respectively (**Supplementary Figure 1**).

**Table 1:** Baseline characteristics in five imputed datasets.

Characteristic	Imputed dataset 1		Imputed dataset 2		Imputed dataset 3		Imputed dataset 4		Imputed dataset 5	
	1060		1060		1060		1060		1060	
Demographic data										
Gender (n, %)										
Female	274 (25.8)		274 (25.8)		274 (25.8)		274 (25.8)		274 (25.8)	
Male	786 (74.2)		786 (74.2)		786 (74.2)		786 (74.2)		786 (74.2)	
Smoking (n, %)										
Yes	219 (20.7)		215 (20.3)		218 (20.6)		222 (20.9)		222 (20.9)	
No	841 (79.3)		845 (79.7)		842 (79.4)		838 (79.1)		838 (79.1)	
BMI (kg/m^2^)	18.42 (16.7, 20.89)		18.42 (16.7, 20.76)		18.49 (16.69, 20.89)		18.42 (16.69, 20.81)		18.42 (16.69, 20.81)	
Laboratory data										
WBC (10^9^/L)	6.43 (5.04, 8.3)		6.42 (5.07, 8.23)		6.38 (5.03, 8.22)		6.42 (5.05, 8.29)		6.42 (5.05, 8.29)	
Hb (g/dL)	12 (10.3, 13.53)		12 (10.3, 13.6)		12.1 (10.3, 13.6)		12 (10.28, 13.50)		12 (10.28, 13.5)	
ESR (mm/h)	21 (9, 42)		21 (9, 43)		21 (9, 42)		21 (9, 42.25)		21 (9, 42.25)	
CRP (mg/dL)	1.56 (0.46, 3.87)		1.44 (0.45, 3.98)		1.48 (0.46, 3.63)		1.48 (0.45, 4.05)		1.48 (0.45, 4.05)	
TT (s)	16.6 (15.8, 17.2)		16.6 (15.8, 17.3)		16.6 (15.8, 17.2)		16.6 (15.88, 17.2)		16.6 (15.88, 17.2)	
APTT (s)	29.75 (27.8, 33.5)		29.7 (27.9, 33.42)		29.7 (27.8, 33.5)		29.7 (27.8, 33.4)		29.7 (27.8, 33.4)	
PT (s)	11.9 (11.1, 13.)		11.9 (11.1, 12.9)		11.9 (11.1, 13)		11.9 (11.1, 12.9)		11.9 (11.1, 12.9)	
Fg (g/L)	3.7 (2.9, 4.62)		3.7 (2.9, 4.6)		3.7 (2.9, 4.63)		3.7 (2.91, 4.63)		3.7 (2.91, 4.63)	
D-Dimer (mg/L)	0.34 (0.19, 0.65)		0.33 (0.19, 0.63)		0.34 (0.19, 0.63)		0.33 (0.19, 0.64)		0.33 (0.19, 0.64)	
ALB (g/dL)	3.7 (3.22, 4.13)		3.69 (3.22, 4.13)		3.7 (3.22, 4.13)		3.7 (3.23, 4.12)		3.7 (3.23, 4.12)	
Crohn’s disease										
Perianal abscess										
Yes	373 (35.2)		373 (35.2)		373 (35.2)		373 (35.2)		373 (35.2)	
No	687 (64.8)		687 (64.8)		687 (64.8)		687 (64.8)		687 (64.8)	
Crohn’s disease data										
Type A classification										
A1	133 (12.5)		133 (12.5)		133 (12.5)		133 (12.5)		133 (12.5)	
A2	788 (74.3)		788 (74.3)		788 (74.3)		788 (74.3)		788 (74.3)	
A3	139 (13.1)		139 (13.1)		139 (13.1)		139 (13.1)		139 (13.1)	
Type B classification										
B1	473 (44.6)		473 (44.6)		473 (44.6)		473 (44.6)		473 (44.6)	
B2	346 (32.6)		346 (32.6)		346 (32.6)		346 (32.6)		346 (32.6)	
B3	241 (22.7)		241 (22.7)		241 (22.7)		241 (22.7)		241 (22.7)	
Type L classification										
L1	268 (25.3)		268 (25.3)		268 (25.3)		268 (25.3)		268 (25.3)	
L2	67 (6.3)		67 (6.3)		67 (6.3)		67 (6.3)		67 (6.3)	
L3	711 (67.1)		711 (67.1)		711 (67.1)		711 (67.1)		711 (67.1)	
L4	14 (1.3)		14 (1.3)		14 (1.3)		14 (1.3)		14 (1.3)	
CDAI										
Remission	133 (12.5)		133 (12.5)		133 (12.5)		133 (12.5)		133 (12.5)	
Mild active	482 (45.5)		482 (45.5)		482 (45.5)		482 (45.5)		482 (45.5)	
Moderate active	392 (37)		392 (37)		392 (37)		392 (37)		392 (37)	
Severe active	53 (5)		53 (5)		53 (5)		53 (5)		53 (5)	
Extrintestinal manifestations										
Extrintestinal manifestations										
Yes		186 (17.5)		186 (17.5)		186 (17.5)		186 (17.5)		186 (17.5)
No		874 (82.5)		874 (82.5)		874 (82.5)		874 (82.5)		874 (82.5)
Oral ulcer										
Yes		98 (9.2)		98 (9.2)		98 (9.2)		98 (9.2)		98 (9.2)
No		962 (90.8)		962 (90.8)		962 (90.8)		962 (90.8)		962 (90.8)
Liver and gallbladder lesions										
Yes		18 (1.7)		18 (1.7)		18 (1.7)		18 (1.7)		18 (1.7)
No		1042 (98.3)		1042 (98.3)		1042 (98.3)		1042 (98.3)		1042 (98.3)
Skin										
Yes		34 (3.2)		34 (3.2)		34 (3.2)		34 (3.2)		34 (3.2)
No		1026 (96.8)		1026 (96.8)		1026 (96.8)		1026 (96.8)		1026 (96.8)
Eye										
Yes		12 (1.1)		12 (1.1)		12 (1.1)		12 (1.1)		12 (1.1)
No		1048 (98.9)		1048 (98.9)		1048 (98.9)		1048 (98.9)		1048 (98.9)
Osteoarthritis										
Yes		38 (3.6)		38 (3.6)		38 (3.6)		38 (3.6)		38 (3.6)
No		1022 (96.4)		1022 (96.4)		1022 (96.4)		1022 (96.4)		1022 (96.4)
Drugs use, n (%)										
5-ASA		95(9.0%)		95(9.0%)		95(9.0%)		95(9.0%)		95(9.0%)
Steroids		152(14.3%)		152(14.3%)		152(14.3%)		152(14.3%)		152(14.3%)
Immunosuppressant		261(24.6%)		261(24.6%)		261(24.6%)		261(24.6%)		261(24.6%)
Biological therapy		667 (62.9)		667 (62.9)		667 (62.9)		667 (62.9)		667 (62.9)

BMI, Body Mass Index; WBC, White Blood Cell Count; CDAI, clinical disease activity index; CRP, C-Reactive Protein; Hb, Hemoglobin; ESR, Erythrocyte Sedimentation Rate; ALB, Albumin; PT, Prothrombin Time; TT, Thrombin Time; Fg, Fibrinogen; APTT, Activated Partial Thromboplastin Time; DD, D-Dimer.

### 3.2 Identification and characterization of candidate predictors for surgical risk

**Figure 2:**
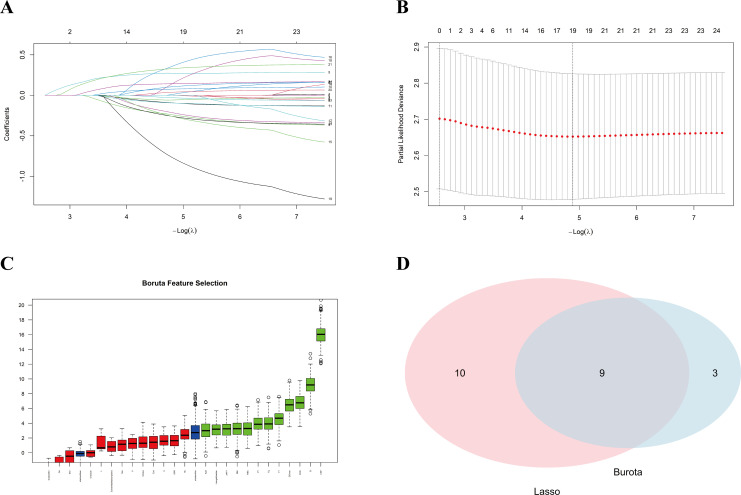
Identification of candidate predictors using least absolute shrinkage and selection operator (LASSO) regression and the Boruta algorithm in imputed dataset 1. **(A)** LASSO coefficient profiles of candidate predictors, illustrating the shrinkage of regression coefficients as the penalty parameter λ changes. The x-axis represents −log(λ), the y-axis represents the coefficient values, and the numbers above indicate the number of nonzero predictors retained in the model. **(B)** Cross-validation plot for selection of the optimal penalty parameter λ in the LASSO model. Red dots indicate the partial likelihood deviance at different λ values, gray error bars indicate the standard errors, and the dotted vertical line marks the optimal λ. **(C)** Results of candidate predictor selection using the Boruta algorithm. Green, red, and blue box plots denote confirmed, rejected, and tentative predictors, respectively. **(D)** Venn diagram showing the overlap between predictors selected by LASSO and Boruta. LASSO identified 19 predictors and Boruta identified 12 predictors, with 9 predictors shared by both methods.

Correlation analysis was performed among the candidate variables, and all pairwise correlation coefficients were below 0.70, indicating the absence of substantial collinearity among the variables (**Supplementary Figure 2**). Subsequently, Lasso-Cox regression using the 1-se criterion and Boruta analysis were conducted (**Figure 2**; **Supplementary Figures 3**-**6**). Variables selected in at least three of the five datasets were defined as candidate predictors, resulting in a total of nine variables for subsequent analyses. These variables were then entered into a Cox regression model, and the pooled results were as follows: BMI (kg/m²) (HR: 0.965, 95% CI: 0.927-1.039, *P* = 0.084), WBC (10^9^/L) (HR: 0.981, 95% CI: 0.927-1.039, *P* = 0.512), CRP (mg/dL) (HR: 1.060, 95% CI: 1.015-1.106, *P* = 0.011), ESR (mm/h) (HR: 1.010, 95% CI: 1.001-1.019, *P* = 0.033), TT (s) (HR: 1.022, 95% CI: 0.935-1.118, *P* = 0.628), Fg (g/L) (HR: 0.759, 95% CI: 0.653-0.882, *P* < 0.001), D-dimer (mg/L) (HR: 1.072, 95% CI: 0.994-1.156, *P* = 0.071), Montreal B classification (HR: 1.252, 95% CI: 1.057-1.482, *P* = 0.010), and CDAI (HR: 1.422, 95% CI: 1.178-1.716, *P* < 0.001) (**Table 2**).

**Table 2:** Pooled multivariable cox regression analysis.

Characteristic	HR (95CI%)	β	Se	Wald	*P*
BMI (kg/m^2^)	0.965(0.927-1.005)	-0.034	0.020	3.031	0.084
WBC	0.981(0.927-1.039)	-0.019	0.029	0.432	0.512
CRP (mg/dL)	1.060(1.015-1.106)	0.058	0.020	8.335	**0.011**
ESR (mm/h)	1.010(1.001-1.019)	0.010	0.005	4.490	**0.033**
TT (s)	1.022(0.935-1.118)	0.022	0.045	0.235	0.628
Fg (g/L)	0.759(0.653-0.882)	-0.274	0.076	13.104	**<0.001**
D-Dimer (mg/L)	1.072(0.994-1.156)	0.070	0.095	3.305	0.071
Montreal B Classification	1.252(1.057-1.482)	0.225	0.086	6.843	**0.010**
CDAI	1.422(1.178-1.716)	0.352	0.095	13.646	**<0.001**

BMI, Body Mass Index; WBC, White Blood Cell Count; CDAI, clinical disease activity index; CRP, C-Reactive Protein; ESR, Erythrocyte Sedimentation Rate; PT, Prothrombin Time; TT, Thrombin Time; Fg, Fibrinogen; APTT, Activated Partial Thromboplastin Time;

Bold values indicate statistically significant results (p < 0.05).

**Figure 3:**
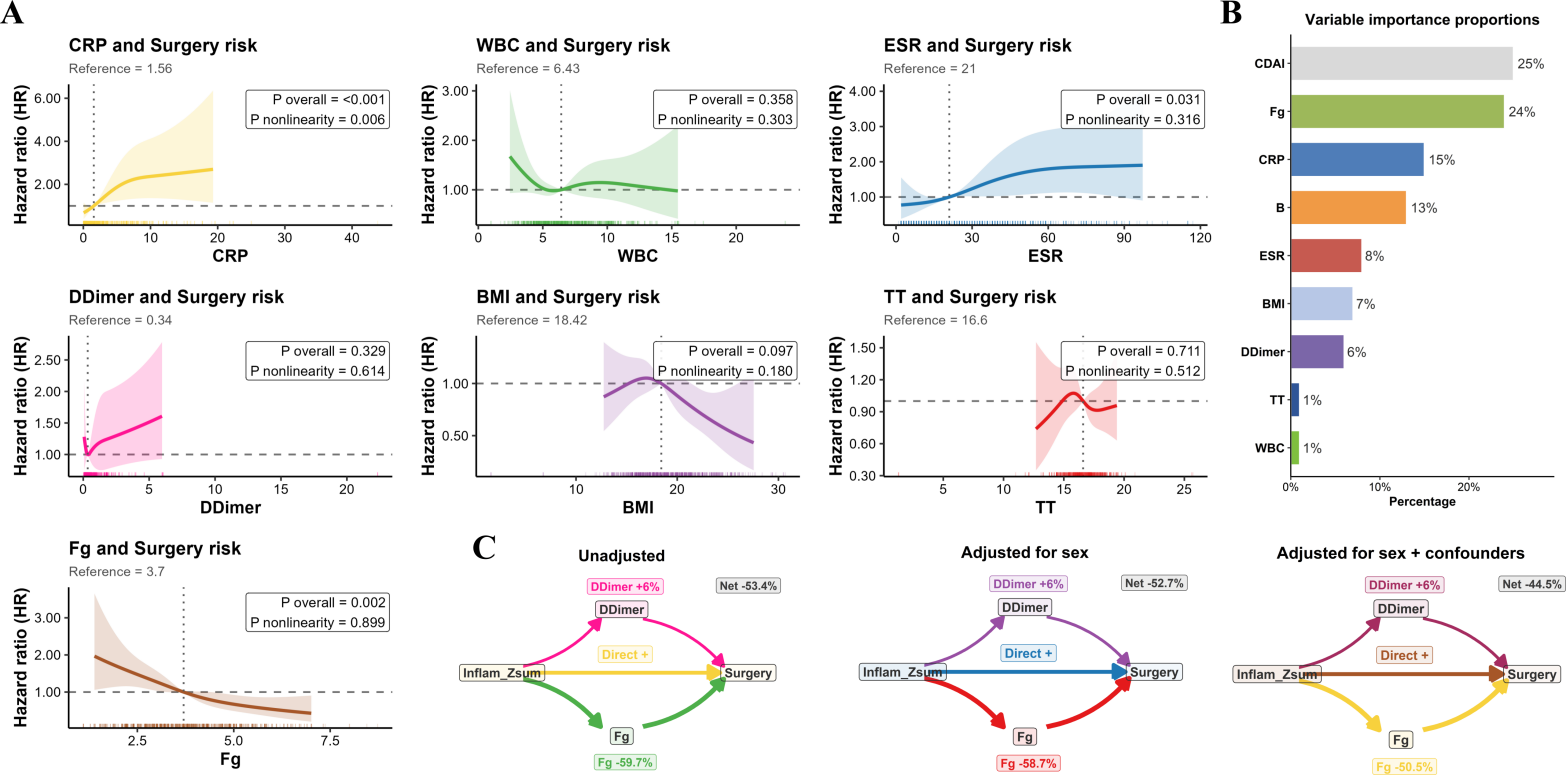
Pooled analyses of selected predictors for surgery risk across five imputed datasets. **(A)** Pooled restricted cubic spline (RCS) curves showing the associations between selected continuous predictors and surgery risk in the cox regression model. Hazard ratios (HRs) and 95% confidence intervals are shown for C-reactive protein (CRP), white blood cell count (WBC), erythrocyte sedimentation rate (ESR), D-dimer, body mass index (BMI), thrombin time (TT), and fibrinogen (Fg). **(B)** Relative importance of predictors in the pooled cox regression model. The bar plot shows the proportional contribution of each predictor to the model. **(C)** Pooled bivariate mediation analyses evaluating the association between the composite inflammatory burden, represented by the combined Z-score of CRP, ESR, and WBC, and surgery risk, with Fg and D-dimer as mediators. Results are shown for the unadjusted model, the model adjusted for sex, and the model adjusted for sex plus confounders.

The pooled RCS analysis based on multivariable Cox regression suggested that WBC (10^9^/L) (*P* for nonlinearity = 0.303), ESR (mm/h) (*P* for nonlinearity = 0.316), D-dimer (mg/L) (*P* for nonlinearity = 0.614), BMI (kg/m²) (*P* for nonlinearity = 0.180), Fg (g/L) (*P* for nonlinearity = 0.899), and TT (s) (*P* for nonlinearity = 0.512) exhibited linear dose-response relationships with surgical risk (**Figure 3A**). CRP (mg/dL) showed a nonlinear association with surgical risk (*P* for nonlinearity = 0.006). Variable importance analysis of the Cox model indicated that CDAI, Fg, CRP, and Montreal B classification were the most important variables, accounting for 25%, 24%, 15%, and 13%, respectively. In contrast, ESR, BMI, D-dimer, TT, and WBC contributed relatively less, accounting for 8%, 7%, 6%, 1%, and 1%, respectively (**Figure 3B**).

As inflammatory and coagulation factors appeared to play important roles, we further performed a parallel multiple mediation analysis (**Figure 3C**). The sum of the Z-scores of WBC, ESR, and CRP was calculated and defined as Inflam_Zsum. In the unadjusted, sex-adjusted, and fully adjusted models, the results showed a consistent pattern: Inflam_Zsum had a significant positive direct effect on surgical risk, whereas D-dimer and Fg exerted significant parallel mediating effects in opposite directions. The indirect effect via D-dimer was positive, whereas that via Fg was negative and stronger in magnitude. Accordingly, in the unadjusted model, the negative indirect effect mediated by Fg offset 59.7% of the direct effect, while the total indirect effect offset 53.4%; after adjustment for sex, these proportions were 58.7% and 52.7%, respectively; and after further adjustment for sex and other confounders, they were 50.5% and 44.5%, respectively. In contrast, the positive indirect effect mediated by D-dimer accounted for only an additional ~6% of the direct effect. Taken together, these results indicate that the negative mediating effect of Fg was stronger than the positive mediating effect of D-dimer, leading to a negative net indirect effect that remained stable across models.

### 3.3 Machine learning model construction and performance evaluation

We developed eight prediction models based on the selected predictors, including Cox, RSF, GBM, CoxBoost, SurvivalSVM, XGBoost, SuperPC, and PLSR-Cox, with hyperparameters shown in **Table 3**. Based on the pooled out-of-fold individual risk scores across the five datasets, GBM achieved the highest C-index of 0.816 (95% CI: 0.789–0.843), followed by XGBoost [0.809 (95% CI: 0.782–0.836)] and RSF [0.789 (95% CI: 0.760–0.818)]; all three models significantly outperformed the Cox model [0.680 (95% CI: 0.643–0.717)] (all *P* < 0.05). PLSR-Cox [0.684 (95% CI: 0.647–0.721)] showed performance comparable to that of the Cox model, whereas CoxBoost [0.670 (95% CI: 0.631–0.709)] showed slightly lower discrimination, although the difference was not statistically significant. In contrast, SurvivalSVM [0.546 (95% CI: 0.505–0.587)] and SuperPC [0.551 (95% CI: 0.516–0.598)] performed less well, and both were significantly inferior to the Cox model (all *P* < 0.05) (**Table 3**).

**Table 3:** Performance metrics of each model, including the C-index, time-dependent Brier score, and integrated Brier score (IBS), and *P*-values for comparisons with the cox model, based on the averaged out-of-fold individual risk predictions across the five imputed datasets.

Model	C-index/*P*-values	1-year Brier	3-year Brier	5-year Brier	IBS	Slope
Cox	0.680(0.643-0.717), Ref	0.091(0.077-0.105)	0.132(0.118-0.146)	0.174(0.155-0.193)	0.132	1.037
RSF	0.789(0.760-0.818),<**0.001**	0.076(0.064-0.088)	0.109(0.096-0.122)	0.145(0.124-0.166)	0.110	0.976
GBM	0.816(0.789-0.843),<**0.001**	0.074(0.062-0.087)	0.103(0.089-0.116)	0.131(0.113-0.149)	0.103	0.987
CoxBoost	0.670(0.631-0.709), 0.353	0.092(0.078-0.107)	0.134(0.120-0.149)	0.179(0.160-0.198)	0.135	1.028
SurvivalSVM	0.546(0.505-0.587),<**0.001**	0.095(0.081-0.110)	0.144(0.129-0.159)	0.188(0.171-0.205)	0.142	0.952
XGBoost	0.809(0.782-0.836),<**0.001**	0.081(0.067-0.095)	0.118(0.104-0.132)	0.153(0.137-0.169)	0.117	0.953
SuperPC	0.551(0.516-0.598),<**0.001**	0.095(0.081-0.110)	0.143(0.128-0.159)	0.188(0.171-0.205)	0.142	0.950
PLSR-Cox	0.684(0.647-0.721), 0.430	0.093(0.078-0.107)	0.139(0.124-0.154)	0.182(0.164-0.199)	0.138	0.980

Hyperparameters of machine learning models:RSF (ntree = 200, nodesize = 30, mtry = 5, nsplit = 3), GBM (n.trees = 600, interaction.depth = 3, n. minobsinnode = 30, shrinkage = 0.01), CoxBoost (stepno = 8, penalty = 50), SurvivalSVM (sgf.sv = 3, sigf = 6, maxiter = 30, margin = 0.1, bound = 30), XGBoost (eta = 0.1, max depth = 1, subsample = 0.5, colsample_bytree = 0.6, gamma = 1), SuperPC (n.components= 4, min.features = 4), PlSR-cox (nt = 3).

Bold values indicate statistically significant results (p < 0.05).

**Figure 4:**
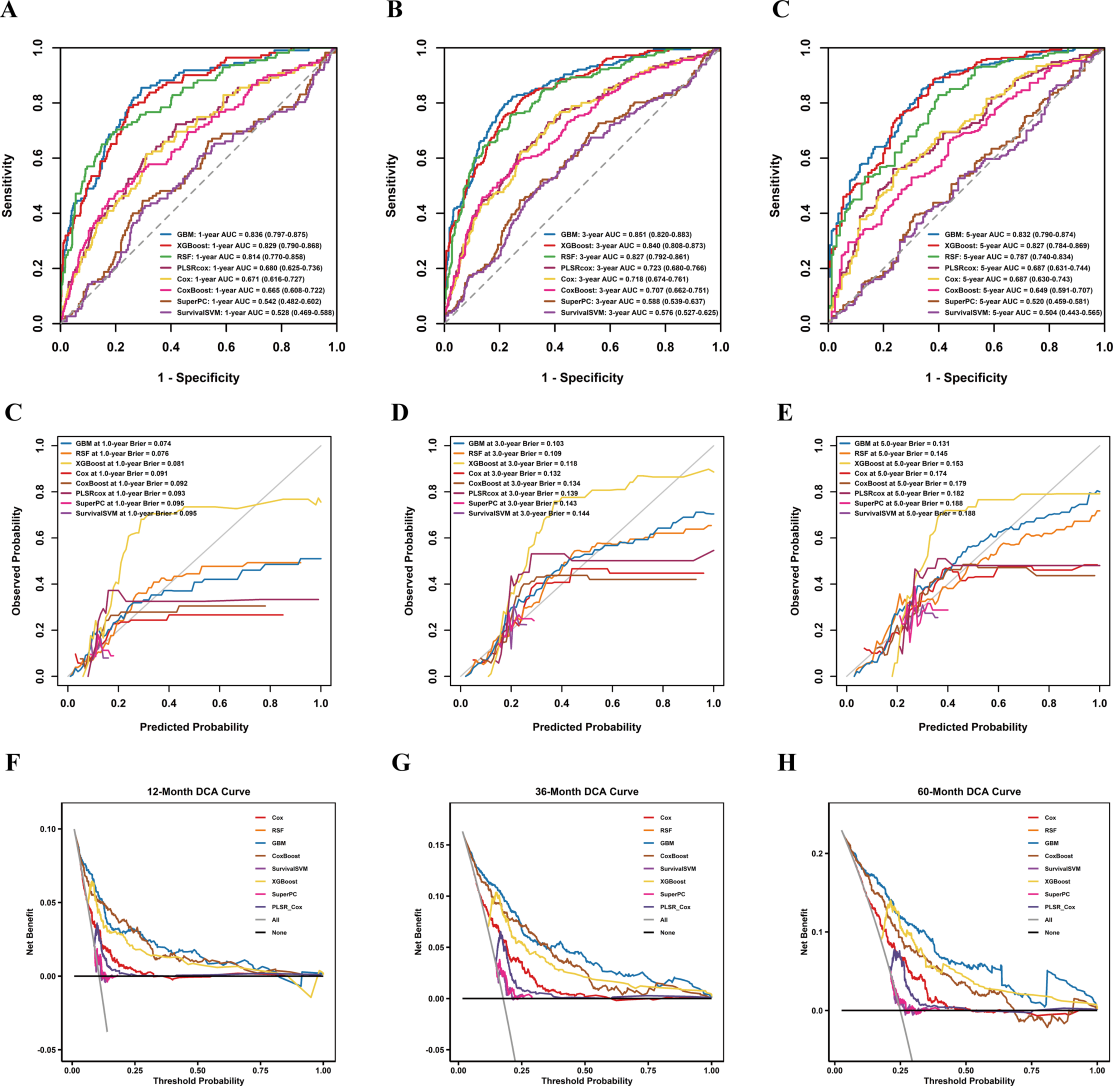
Discrimination, calibration, and clinical utility of different models for predicting surgery risk, based on averaged out-of-fold individual risk predictions across five imputed datasets. **(A–C)** Time-dependent receiver operating characteristic (ROC) curves of the models for predicting surgery risk at 1, 3, and 5 years, respectively. The corresponding areas under the curve (AUCs) are shown for each model. **(D–F)** Calibration curves of the models for predicting surgery risk at 1, 3, and 5 years, respectively. The corresponding Brier scores are provided for each model. **(G–I)** Decision curve analysis (DCA) curves of the models for predicting surgery risk at 1, 3, and 5 years, respectively, illustrating the net clinical benefit of each model across a range of threshold probabilities. Cox, Cox proportional hazards model; RSF, random survival forest; XGBoost, extreme gradient boosting; GBM, gradient boosting machine; CoxBoost, likelihood-based boosting for the Cox proportional hazards model; PLSR-Cox, partial least squares regression for Cox regression; SuperPC, supervised principal components; Survival-SVM, survival support vector machine.

Time-dependent ROC analysis yielded results consistent with the C-index findings. GBM again showed the best predictive performance, with AUCs of 0.836, 0.851, and 0.832 at 1, 3, and 5 years, respectively, followed by XGBoost (0.829, 0.840, and 0.827) and RSF (0.814, 0.827, and 0.787). CoxBoost and PLSR-Cox again showed no clear improvement over the Cox model, whereas SuperPC (0.542, 0.588, and 0.520) and SurvivalSVM (0.528, 0.576, and 0.504) continued to show poor predictive performance (**Figures 4A–C**).

Calibration plots showed good agreement between predicted and observed risks for GBM. Consistently, GBM yielded the lowest Brier scores at 1, 3, and 5 years (0.074, 0.103, and 0.131, respectively), followed by RSF (0.076, 0.109, and 0.145) and XGBoost (0.081, 0.118, and 0.153). GBM also achieved the lowest integrated Brier score (IBS = 0.103) and the calibration slope closest to 1 (0.987). (**Table 3****;** **Figures 4D–F**). In addition, decision curve analysis showed that GBM provided the greatest net clinical benefit at each evaluated time point (**Figures 4D–F**). Separate analyses of the five datasets yielded findings consistent with the pooled results, with no significant between-dataset differences (**Supplementary Figures 7**-**15**; **Supplementary Tables 1**-**7**). Moreover, when model performance was averaged across the five datasets before five-fold cross-validation, the estimates were slightly better, suggesting that five-fold cross-validation reduced some degree of optimism in model performance (**Supplementary Table 8**). Taken together, these results identified GBM as the optimal model.

### 3.4 Risk stratification and reclassification of the GBM and cox models

**Figure 5:**
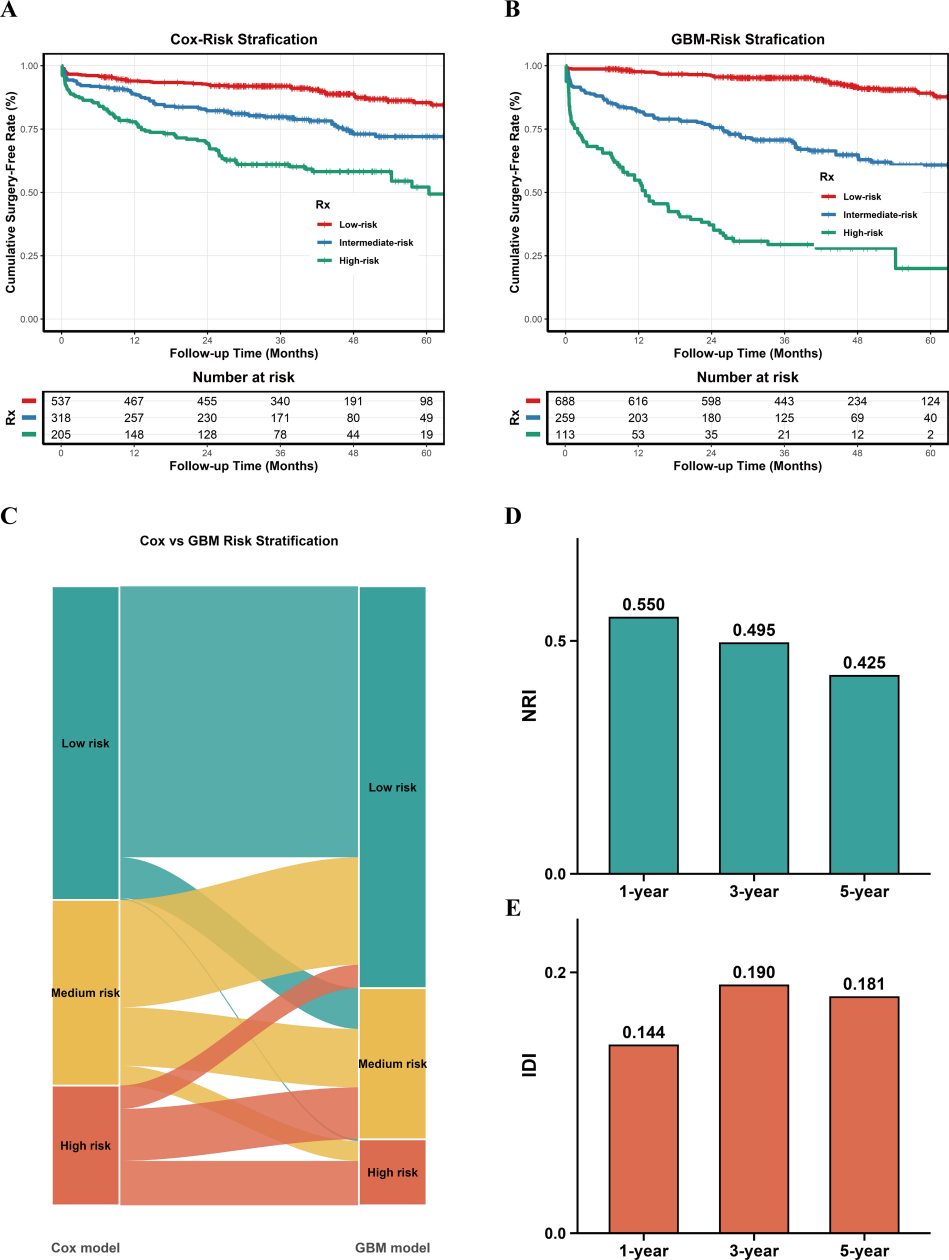
Reclassification and comparative risk-stratification performance of the Cox and GBM models, based on averaged out-of-fold individual risk predictions across the five imputed datasets. **(A, B)** Kaplan–Meier curves of cumulative surgery-free rates according to risk groups defined by the cox model **(A)** and the GBM model **(B)**, respectively. **(C)** Sankey diagram illustrating the redistribution of patients among the low-, intermediate-, and high-risk groups between the cox and GBM models. **(D, E)** Net reclassification improvement (NRI) **(D)** and integrated discrimination improvement (IDI) **(E)** of the GBM-based risk stratification relative to the cox-based risk stratification at 1, 3, and 5 years.

Risk stratification based on the predicted risk scores from the GBM and Cox models showed significant differences in cumulative surgery-free survival among the low-, intermediate-, and high-risk groups at 1, 3, and 5 years (**Figures 5A, B**). In the Cox model, the 1-, 3-, and 5-year surgery-free survival rates were 94.0%, 91.9%, and 85.4% in the low-risk group, 88.8%, 79.8%, and 72.0% in the intermediate-risk group, and 77.9%, 61.0%, and 49.3% in the high-risk group, respectively. In the GBM model, the 1-, 3-, and 5-year surgery-free survival rates were 97.6%, 95.2%, and 89.1% in the low-risk group, 82.1%, 70.6%, and 60.8% in the intermediate-risk group, and 54.8%, 29.4%, and 20.0% in the high-risk group, respectively.

The Sankey diagram illustrated substantial reclassification from the Cox model to the GBM model: 17.4% of Cox intermediate-risk patients and 3.8% of Cox high-risk patients were reassigned to the GBM low-risk group; 6.6% of Cox low-risk patients and 8.4% of Cox high-risk patients were reassigned to the GBM intermediate-risk group; and 0.3% of Cox low-risk patients and **3.2%** of Cox intermediate-risk patients were reassigned to the GBM high-risk group (**Figure 5C**). The NRI and IDI further supported the improvement in classification performance, with NRI values of 0.550, 0.495, and 0.425 at 1, 3, and 5 years, respectively, and corresponding IDI values in the training cohort of 0.144, 0.190, and 0.181 (**Figures 5D, E**).

### 3.5 Association of biologics with surgical risk across GBM-defined risk strata

We further evaluated the association between biologic use and surgical risk across GBM-defined risk strata. Before IPTW, baseline characteristics were imbalanced between the biologics and non-biologics groups within the low-, intermediate-, and high-risk strata, with some covariates showing significant differences. Therefore, IPTW was separately performed within each risk stratum in each of the five imputed datasets, and good post-weighting balance was achieved (**Supplementary Tables 9**-**18**).

**Figure 6:**
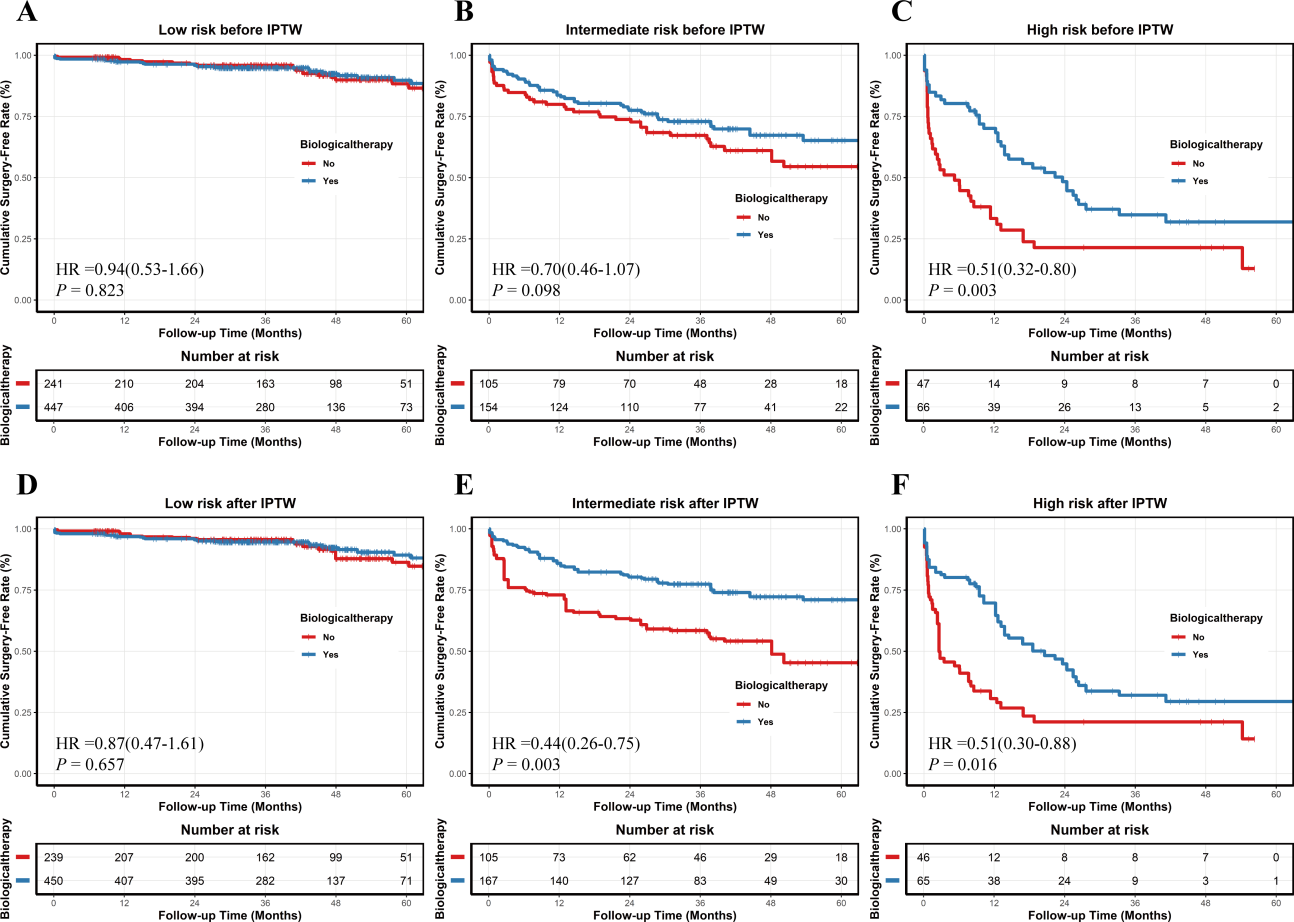
Comparison of cumulative surgery-free rates between patients treated with and without biologics across GBM-defined risk strata before and after inverse probability of treatment weighting (IPTW) in imputed dataset 1. **(A–C)** Kaplan–Meier curves of cumulative surgery-free rates in the low-risk **(A)**, intermediate-risk **(B)**, and high-risk **(C)** groups before IPTW. **(D–F)** Kaplan–Meier curves of cumulative surgery-free rates in the low-risk **(D)**, intermediate-risk **(E)**, and high-risk **(F)** groups after IPTW. HRs and *P* values shown in the plots are pooled across the five imputed datasets.

The pooled analyses showed that in the low-risk stratum, biologic use was not significantly associated with a reduced risk of surgery either before or after IPTW adjustment (before IPTW: pooled HR = 0.94, 95% CI: 0.53-1.66, pooled *P* = 0.823; after IPTW: pooled HR = 0.87, 95% CI: 0.47-1.61, pooled *P* = 0.657). In the intermediate-risk stratum, biologic use was not significantly associated with surgical risk reduction before IPTW adjustment (pooled HR = 0.70, 95% CI: 0.46-1.07, pooled *P* = 0.098), but was significantly associated with a lower surgical risk after IPTW adjustment (pooled HR = 0.44, 95% CI: 0.26-0.75, pooled *P* = 0.003). In the high-risk stratum, biologic use was significantly associated with a reduced risk of surgery both before and after IPTW adjustment (before IPTW: pooled HR = 0.51, 95% CI: 0.32-0.80; pooled *P* = 0.003, after IPTW: pooled HR = 0.51, 95% CI: 0.30-0.88, pooled *P* = 0.016) (**Figure 6**; **Supplementary Figure 16****).**

### 3.6 SHAP interpretation of the GBM model

**Figure 7:**
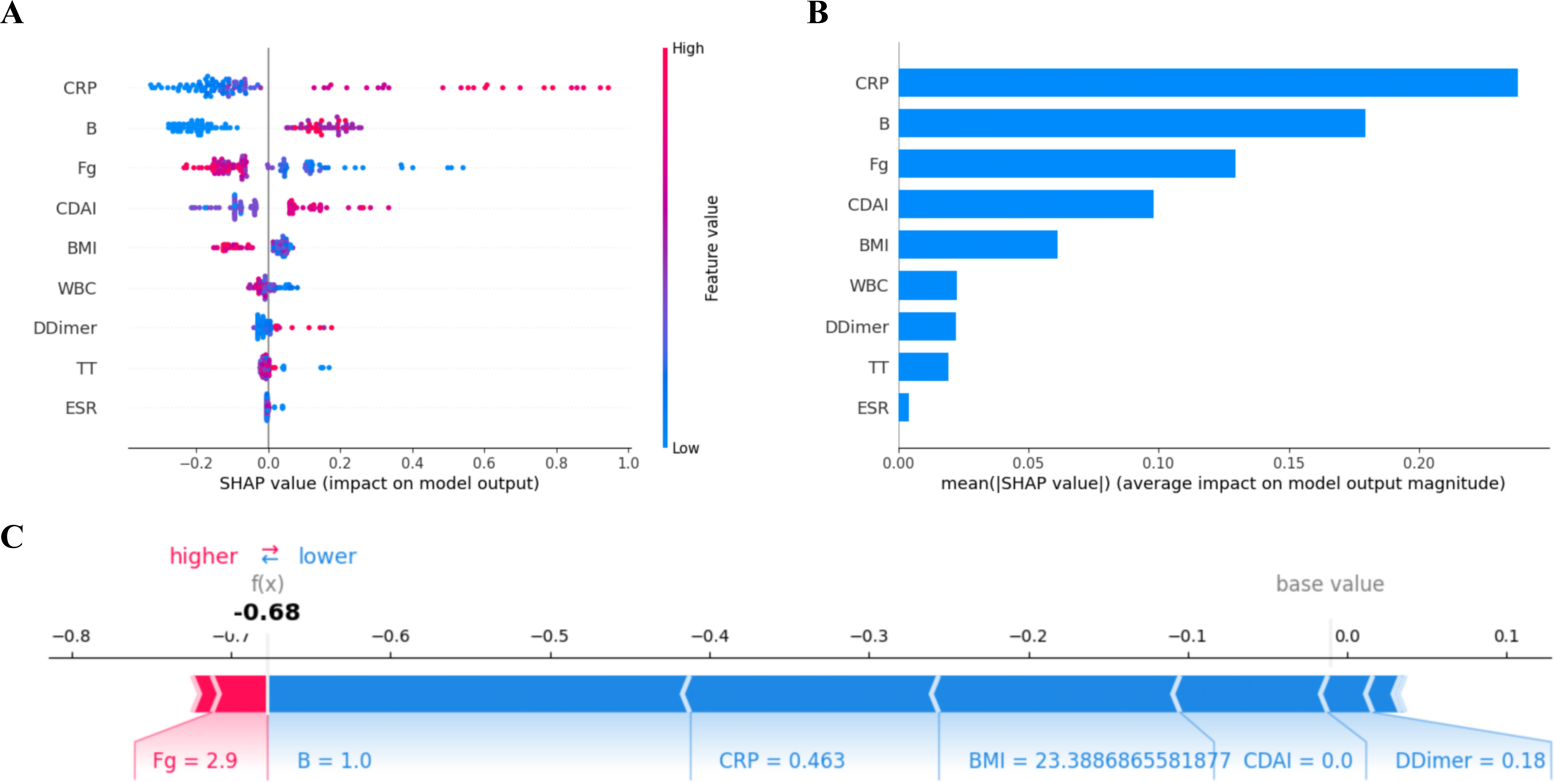
SHapley Additive exPlanations (SHAP) interpretation of the gradient boosting machine (GBM) model. **(A)** SHAP summary plot showing the distribution of SHAP values for each predictor in the GBM model. Each dot represents one individual, and the color indicates the feature value from low to high. **(B)** Bar plot of mean absolute SHAP values, showing the relative importance of predictors in the GBM model. **(C)** Representative SHAP force plot for an individual prediction, illustrating how each predictor contributes to the model output by pushing the prediction higher or lower relative to the base value.

SHAP analysis was performed to further interpret the contribution of individual variables to the model output. The global SHAP summary plot (**Figure 7A**) showed that CRP, Montreal B classification, Fg, and CDAI were the most influential features driving model prediction. In general, higher CRP values, B-type classification, and CDAI scores were associated with positive SHAP values, indicating a tendency to increase the model output, whereas higher Fg and BMI values were more likely to be associated with negative SHAP values, suggesting an inhibitory effect on the prediction. The SHAP feature importance ranking based on mean absolute SHAP values (**Figure 7B**) further confirmed that CRP had the greatest overall impact on the model, followed by Montreal B classification, Fg, CDAI, and BMI, while WBC, D-dimer, TT, and ESR contributed relatively little to the model output.

For individual-level interpretation, the SHAP force plot (**Figure 7C**) illustrated how each variable contributed to the prediction in a representative sample. Starting from a baseline value near zero, the combined effects of the included features shifted the final prediction to −0.68. Among these, Montreal B = 1.0 (represent B1), CRP = 0.463, BMI = 23.39, CDAI = 0.0 (represent remission), and D-dimer = 0.18 mainly drove the prediction toward a lower output, whereas Fg = 2.97 exerted a modest positive contribution. Overall, the cumulative negative effects of multiple variables outweighed the positive contribution of Fg, resulting in a markedly decreased final prediction for this sample.

SHAP dependence plots further illustrated the relationships between feature values and model output. CRP, D-dimer, CDAI, and Montreal B classification were generally positively associated with SHAP values, whereas Fg and BMI were generally negatively associated. WBC showed both positive and negative SHAP values across its range, suggesting a non-linear and value-dependent effect. In contrast, ESR showed only limited influence on the model output (**Supplementary Figure 17**).

### 3.7 Web platform development

**Figure 8:**
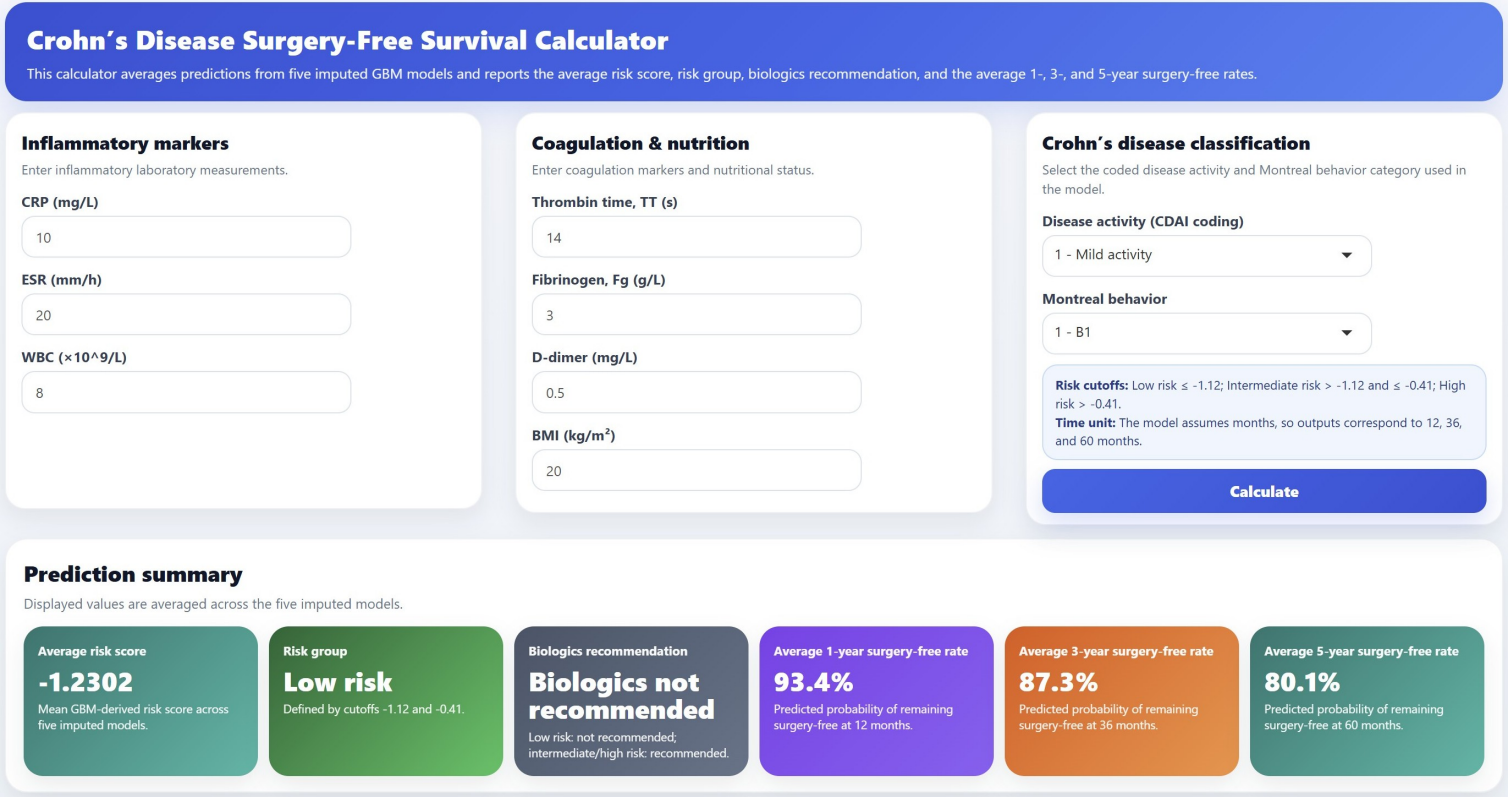
Screenshot of the web-based calculator for individualized prediction of surgery-free survival in patients with Crohn’s disease based on the optimal GBM model.

We presented the optimal model (GBM) through an interactive web application built using the R package Shiny, which allows individualized prediction of surgery-free survival in patients with Crohn’s disease (URL: https://crohndisease.shinyapps.io/Crohndisease/). The web-based calculator takes routinely available clinical and laboratory variables as input and reports the average risk score, risk group, biologics recommendation, and the average 1-, 3-, and 5-year surgery-free rates based on predictions averaged across five imputed GBM models. **Figure 8** provides an example. Consider a patient with a CRP level of 10 mg/L, ESR of 20 mm/h, WBC count of 8 × 10^9^/L, thrombin time of 14 s, fibrinogen of 3 g/L, D-dimer of 0.5 mg/L, and BMI of 20 kg/m^2^. The patient is coded as having mild disease activity and Montreal behavior B1. After entering these values into the calculator, the model generates an average risk score of -1.2302, classifies the patient as low risk, and indicates that biologics are not recommended. The predicted probabilities of remaining surgery-free at 1, 3, and 5 years are 93.4%, 87.3%, and 80.1%, respectively.

## 4 Discussion

### 4.1 Main findings and interpretation of model performance

In this study, we developed and validated a gradient boosting machine (GBM)-based model for predicting abdominal surgery in Crohn’s disease (CD). The final model integrated inflammatory, coagulation-related, nutritional, and disease-behavior variables, and showed better discrimination, calibration, and clinical net benefit than the conventional Cox model (13–17). The GBM model also improved risk stratification and reclassification, suggesting that long-term surgical risk in CD is shaped not only by individual predictors but also by nonlinear relationships and complex interactions among inflammatory burden, coagulation status, nutritional condition, disease behavior, and overall disease activity (6, 13–17). This interpretation is consistent with our findings that CRP showed a nonlinear association with surgical risk and that WBC displayed a value-dependent effect in SHAP analyses. In addition, the model identified subgroups with differential associations between biologic therapy and surgical outcomes, highlighting its potential value for personalized disease management (4, 6, 11).

### 4.2 Association between predictors with surgery risk

A major biological implication of our findings is that long-term surgical progression in CD appears to be shaped by a close interplay between systemic inflammation and coagulation-related responses. Persistent intestinal inflammation promotes epithelial injury, endothelial activation, leukocyte recruitment, and transmural tissue damage, thereby driving bowel wall remodeling and progression to structuring or penetrating complications (1, 2, 25–29). In this context, CRP, ESR, and WBC reflect different but complementary aspects of inflammatory burden. Their collective importance in our model supports the view that uncontrolled inflammation remains a fundamental upstream driver of bowel damage and surgery risk in CD (1, 2, 25–29).

At the same time, inflammation and coagulation are biologically coupled processes. Inflammatory cytokines and endothelial injury can activate tissue factor-dependent coagulation, increase thrombin generation, and promote fibrin formation, whereas coagulation products may in turn influence inflammatory signaling, vascular integrity, leukocyte trafficking, and tissue remodeling (7, 9, 30–32). This framework helps interpret our mediation findings. D-dimer showed a modest positive mediating effect, consistent with the concept that elevated D-dimer reflects enhanced fibrin formation and degradation, ongoing coagulation activation, and a more prothrombotic inflammatory milieu (7, 9, 10, 33). In contrast, fibrinogen showed an inverse association with surgical risk and a stronger negative mediating effect, suggesting that it may capture a distinct aspect of the host response (7, 8, 30, 34–36).

This result should be interpreted cautiously. Although fibrinogen is traditionally regarded as an acute-phase reactant, it may also reflect compensatory hemostatic and reparative responses. Fibrinogen-derived fibrin can provide a provisional matrix for wound healing, support vascular integrity, and facilitate mucosal repair under conditions of ongoing inflammatory injury (8, 30–32, 34–36). Therefore, in CD, a higher fibrinogen level may not simply indicate more severe inflammation, but may also reflect a more effective repair response in some patients (8, 34–36). Importantly, our findings do not establish a protective causal role for fibrinogen; rather, they suggest that the biological meaning of coagulation-related markers in CD is heterogeneous and context-dependent. In this sense, the inflammation–coagulation axis may not follow a simple “more coagulation, worse outcome” pattern, but instead reflect overlapping processes of inflammatory injury, hypercoagulability, fibrin turnover, and tissue repair (7, 8, 30–36).

This framework also explains why not all coagulation-related markers contributed equally to prediction. In our study, Fg was among the most influential variables, whereas D-dimer contributed more modestly and thrombin time contributed little. These differences suggest that coagulation-related biomarkers should not be interpreted as a uniform group, and that Fg may provide clinically distinct information compared with D-dimer or thrombin time (7–10). From a broader perspective, this inflammation–coagulation interplay may help connect systemic inflammatory activity with structural bowel progression, which is also consistent with the strong contributions of Montreal B classification and CDAI in the final model (2, 37–41).

### 4.3 Clinical implications for biologic therapy

One of the most clinically relevant findings of this study is that the model may help identify subgroups with differential associations between biologic therapy and surgical outcomes. After IPTW adjustment, biologic use was not significantly associated with reduced surgical risk in the low-risk group, but it was associated with significantly lower surgical risk in the intermediate- and high-risk groups (4, 11, 12). This pattern suggests that the association between biologic therapy and surgical outcomes may not be uniform across patients, and that risk stratification may provide potentially useful information for treatment decision-making. Patients at low predicted risk may be less likely to require treatment escalation, whereas those at intermediate or high predicted risk may warrant closer evaluation for earlier or more intensive biologic therapy (4, 11, 12).

However, these treatment-related findings should be interpreted with caution. Although we performed additional IPTW-based analyses to reduce baseline confounding between the biologic and non-biologic therapy groups, complete information on the exact duration of biologic therapy was unavailable for some patients, which may introduce time-related bias. In addition, this study was not designed within a formal causal inference framework, and important issues such as time-dependent treatment exposure and confounding by indication could not be fully addressed. Therefore, the observed associations between biologic therapy and surgical risk across different risk strata should be considered exploratory rather than confirmatory. Nevertheless, these findings support the potential value of combining prognostic modeling with treatment-informative stratification in personalized CD management (6). Future studies with prospective longitudinal data and explicit causal methods are needed to more rigorously evaluate the potential treatment effect of biologic therapy in different risk groups.

### 4.4 Limitations

#### 4.4.1 Limitations related to model development, validation, and generalizability

Several limitations should be acknowledged. First, the present model should still be regarded as developmental rather than as a fully validated clinical tool, mainly because external validation in independent cohorts has not yet been performed. In addition, as this was a retrospective single-center study, the model may reflect center-specific case-mix, clinical workflows, and biologic treatment patterns. Therefore, its transportability to other institutions, time periods, or patient populations remains uncertain. Although model performance was evaluated across five imputed datasets using cross-validation, and the gap between apparent and cross-validated performance suggested a reduction in optimism, bootstrap-based resampling validation was not performed. Therefore, some residual optimism may still remain in the reported performance estimates. In addition, calibration assessment also had limitations. This study did not summarize calibration using an intercept-based framework directly analogous to that used in logistic regression, because survival models do not have a directly comparable single global calibration intercept. Therefore, calibration comparisons across models were based mainly on calibration plots, Brier-based measures, and slope, and should be interpreted with appropriate caution. Future studies should include temporal and multicenter geographic external validation, with recalibration or model updating where appropriate.

#### 4.4.2 Limitations related to variables, treatment analysis, and interpretation

Important dynamic variables during follow-up were not incorporated into the current model. These included treatment adherence, treatment duration, regimen modifications, and time-dependent treatment exposure. As a result, the comprehensiveness of the model and its real-world applicability may have been affected. In addition, the biologic therapy analysis was observational. Although IPTW adjustment was applied, residual confounding and confounding by indication cannot be fully excluded. Moreover, the model included only routinely available variables. Therefore, it did not incorporate endoscopic, radiologic, genetic, or histopathologic information that might further improve prediction. In addition, although quality control procedures were implemented during data collection, laboratory indicators may still be affected by short-term fluctuations. This should be considered when interpreting the findings. Abdominal surgery was analyzed as a composite endpoint. Therefore, heterogeneity in surgical indications may have influenced the observed associations. Finally, mediation analysis and SHAP interpretation should be viewed as explanatory rather than causal, especially with respect to the observed role of Fg.

## 5 Conclusion

Integrating inflammatory and coagulation biomarkers with disease-related features substantially improves long-term surgical risk stratification in Crohn’s disease. The GBM-based model demonstrated superior predictive performance over the conventional Cox model and provided clinically interpretable risk estimates. Moreover, this biomarker-informed framework may help identify patients who are more likely to benefit from biologic therapy, particularly those at intermediate and high risk. These findings support the potential value of combining inflammatory and coagulation signatures for individualized prognosis assessment and treatment decision-making in CD.

## Data Availability

The datasets utilized in the present study can be obtained from the corresponding author upon reasonable request. The code for data analysis is available in the **Supplementary Materials**.
