# Supplementary material for: Integrating inflammatory and coagulation biomarkers for surgical risk stratification and treatment benefit assessment in Crohn’s disease
Source: Front Immunol. 2026 May 7;17:1657279. doi: 10.3389/fimmu.2026.1657279 (PMC13189891; doi:10.3389/fimmu.2026.1657279)
Supplement: Supplementary file 1 [file DataSheet1.pdf]

```
##############################################################
## Internal validation workflow for survival ML models
## Outcome: Time / Surgery
## Data object name: data
##
## Supports:
##      1) data.frame (single imputed dataset)
##      2) mice::mids object
##      3) list of imputed data.frames
##
## Main workflow:
##      - Same 5-fold partition across all imputations
##      - Within each imputed dataset: 5-fold CV hyperparameter tuning
##      - Across imputations: two-stage final hyperparameter selection
##      - Final performance based on out-of-fold predictions
##      - Main presentation based on averaged individual-level OOF predictions
##      - Parallel per-imputation evaluation for supplementary results
##############################################################

#############################
## 0. Packages
#############################
required_pkgs <- c(
  "survival",
  "randomForestSRC",
  "gbm",
  "CoxBoost",
  "survivalsvm",
  "xgboost",
```

```r
  "plsRcox",
  "timeROC",
  "ggplot2",
  "dcurves",
  "writexl"
)

missing_pkgs <- required_pkgs[!vapply(required_pkgs, requireNamespace, logical(1), quietly = TRUE)]
if (length(missing_pkgs) > 0) {
  stop(
    "请先安装以下 R 包：\n",
    paste(missing_pkgs, collapse = ", ")
  )
}

invisible(lapply(required_pkgs, library, character.only = TRUE))

if (inherits(data, "mids") && !requireNamespace("mice", quietly = TRUE)) {
  stop("data 是 mids 对象，但未安装 mice 包，请先 install.packages('mice')")
}

##############################
## 1. User settings
##############################
SEED <- 123
set.seed(SEED)
```

```
## 如果是多个插补数据集，建议 5；单个 data.frame 也能跑

N_FOLDS <- 5


## 评估时间点：请按你的研究真实时间点修改

## 例如单位是月，可设为 c(12, 24, 36)

eval_times <- c(12, 24, 36)


## 校准曲线和 DCA 使用的时间点，必须在 eval_times 中

dca_time <- 36


## 输出目录

output_dir <- "internal_validation_outputs"
if (!dir.exists(output_dir)) dir.create(output_dir, recursive = TRUE)

############################
## 2. Input handling
############################
get_imputed_datasets <- function(data) {
  if (inherits(data, "mids")) {
    ds <- mice::complete(data, action = "all")
  } else if (is.list(data) && all(vapply(data, is.data.frame, logical(1)))) {
    ds <- data
  } else if (is.data.frame(data)) {
    ds <- list(data)
  } else {

    stop("data 必须是 data.frame、mice::mids 对象，或由多个插补后 data.frame 组成的 list。
```

```
")
  }

  ds <- lapply(ds, function(d) {
    d <- as.data.frame(d)

    if (!all(c("Time", "Surgery") %in% names(d))) {
      stop("每个数据集必须包含列名：Time 和 Surgery")
    }

    d$Time <- as.numeric(d$Time)
    d$Surgery <- as.integer(d$Surgery)

    if (!all(d$Surgery %in% c(0, 1))) {
      stop("Surgery 必须是 0/1 结局变量。")
    }

    d$.row_id <- seq_len(nrow(d))
    d
  })

  message("识别到 ", length(ds), " 个插补后数据集。")
  ds
}

imputed_list <- get_imputed_datasets(data)
N_IMP <- length(imputed_list)
```

```
## 预测变量：自动去除 Time、Surgery、.row_id

predictors <- setdiff(names(imputed_list[[1]]), c("Time", "Surgery", ".row_id"))


if (length(predictors) == 0) {

  stop("未识别到预测变量。请检查数据列。")

}


message("预测变量个数：", length(predictors))

message("预测变量：", paste(predictors, collapse = ", "))


############################
## 3. Shared 5-fold partition
############################
make_folds <- function(dat, k = 5, seed = 123) {
  set.seed(seed)

  if (length(unique(dat$Surgery)) < 2) {

    stop("结局变量 Surgery 只有一个水平，无法进行分层 5-fold CV。")

  }

  fold_id <- integer(nrow(dat))

  idx1 <- which(dat$Surgery == 1)
  idx0 <- which(dat$Surgery == 0)
```

```
  fold_id[idx1] <- sample(rep(seq_len(k), length.out = length(idx1)))
  fold_id[idx0] <- sample(rep(seq_len(k), length.out = length(idx0)))

  fold_id
}

fold_id <- make_folds(imputed_list[[1]], k = N_FOLDS, seed = SEED)

#############################
## 4. Helper functions
#############################
bt <- function(x) paste0("`", x, "`")

make_surv_formula <- function(predictors) {
  as.formula(
    paste("Surv(Time, Surgery) ~", paste(bt(predictors), collapse = " + "))
  )
}

preprocess_fold <- function(train_df, valid_df, predictors) {
  train_df <- as.data.frame(train_df)
  valid_df <- as.data.frame(valid_df)

  num_vars <- predictors[vapply(train_df[, predictors, drop = FALSE], is.numeric, logical(1))]

  if (length(num_vars) > 0) {
    mu <- sapply(train_df[, num_vars, drop = FALSE], mean, na.rm = TRUE)
    sdv <- sapply(train_df[, num_vars, drop = FALSE], sd, na.rm = TRUE)
    sdv[is.na(sdv) | sdv == 0] <- 1
```

```
    train_df[, num_vars] <- sweep(train_df[, num_vars, drop = FALSE], 2, mu, "-")
    train_df[, num_vars] <- sweep(train_df[, num_vars, drop = FALSE], 2, sdv, "/")

    valid_df[, num_vars] <- sweep(valid_df[, num_vars, drop = FALSE], 2, mu, "-")
    valid_df[, num_vars] <- sweep(valid_df[, num_vars, drop = FALSE], 2, sdv, "/")
  }

  list(train = train_df, valid = valid_df)
}

build_xmat <- function(train_df, valid_df, predictors) {
  mm_formula <- as.formula(
    paste("~", paste(bt(predictors), collapse = " + "), "-1")
  )

  x_train <- model.matrix(mm_formula, data = train_df)
  x_valid <- model.matrix(mm_formula, data = valid_df)

  miss_cols <- setdiff(colnames(x_train), colnames(x_valid))
  extra_cols <- setdiff(colnames(x_valid), colnames(x_train))

  if (length(miss_cols) > 0) {
    add_mat <- matrix(0, nrow = nrow(x_valid), ncol = length(miss_cols))
    colnames(add_mat) <- miss_cols
    x_valid <- cbind(x_valid, add_mat)
  }

  if (length(extra_cols) > 0) {
```

```
    x_valid <- x_valid[, setdiff(colnames(x_valid), extra_cols), drop = FALSE]
  }

  x_valid <- x_valid[, colnames(x_train), drop = FALSE]

  list(x_train = x_train, x_valid = x_valid)
}

get_cindex <- function(time, status, score) {
  cc <- survival::concordance(Surv(time, status) ~ score, reverse = TRUE)
  as.numeric(cc$concordance)
}

rawscore_to_risk <- function(train_time, train_status, train_score, valid_score, times) {
  tmp <- data.frame(
    Time = train_time,
    Surgery = train_status,
    score = train_score
  )

  cal_fit <- coxph(Surv(Time, Surgery) ~ score, data = tmp, ties = "breslow", x = TRUE)

  lp_valid <- as.numeric(predict(cal_fit, newdata = data.frame(score = valid_score), type = "lp"))

  bh <- basehaz(cal_fit, centered = FALSE)
  H0_t <- approx(
    x = bh$time,
    y = bh$hazard,
    xout = times,
```

```
    method = "constant",
    rule = 2,
    f = 0
  )$y

  risk_mat <- sapply(H0_t, function(h) {
    1 - exp(-h * exp(lp_valid))
  })

  if (is.vector(risk_mat)) {
    risk_mat <- matrix(risk_mat, ncol = 1)
  }

  colnames(risk_mat) <- paste0("risk_t", times)

  list(
    lp_valid = lp_valid,
    risk_valid = risk_mat
  )
}

calc_brier_ipcw <- function(time, status, pred_risk, t0) {
  fit_cens <- survfit(Surv(time, 1 - status) ~ 1)

  Ghat <- function(u) {
    g <- summary(fit_cens, times = u, extend = TRUE)$surv
    g[g < 1e-6] <- 1e-6
    g
  }
```

```
  y_t <- as.integer(time <= t0 & status == 1)
  w <- rep(0, length(time))

  idx_event_before_t <- which(time <= t0 & status == 1)
  idx_after_t <- which(time > t0)

  if (length(idx_event_before_t) > 0) {
    w[idx_event_before_t] <- 1 / Ghat(pmax(time[idx_event_before_t] - 1e-08, 0))
  }
  if (length(idx_after_t) > 0) {
    w[idx_after_t] <- 1 / Ghat(t0)
  }

  mean(w * (y_t - pred_risk)^2, na.rm = TRUE)
}

calc_time_auc <- function(time, status, marker, times) {
  roc_obj <- timeROC::timeROC(
    T = time,
    delta = status,
    marker = marker,
    cause = 1,
    times = times,
    iid = TRUE
  )

  data.frame(
    time = times,
```

```r
    AUC = as.numeric(roc_obj$AUC)
  )
}

calc_time_roc_object <- function(time, status, marker, times) {
  timeROC::timeROC(
    T = time,
    delta = status,
    marker = marker,
    cause = 1,
    times = times,
    iid = TRUE
  )
}

make_calibration_df <- function(time, status, pred_risk, t0, n_groups = 10) {
  qs <- quantile(pred_risk, probs = seq(0, 1, length.out = n_groups + 1), na.rm = TRUE)
  qs <- unique(qs)

  if (length(qs) < 3) {
    qs <- seq(min(pred_risk, na.rm = TRUE), max(pred_risk, na.rm = TRUE), length.out = 3)
  }

  grp <- cut(pred_risk, breaks = qs, include.lowest = TRUE, ordered_result = TRUE)

  out <- lapply(split(seq_along(pred_risk), grp), function(idx) {
    sf <- survfit(Surv(time[idx], status[idx]) ~ 1)
    s0 <- summary(sf, times = t0, extend = TRUE)$surv[1]
    obs_risk <- 1 - s0
```

```
    data.frame(
      group = as.character(grp[idx][1]),
      n = length(idx),
      mean_pred = mean(pred_risk[idx], na.rm = TRUE),
      obs_risk = obs_risk
    )
  })

  do.call(rbind, out)
}

plot_calibration_curve <- function(cal_df, model_name, t0, imp_label = "Main") {
  ggplot(cal_df, aes(x = mean_pred, y = obs_risk)) +
    geom_point(size = 3) +
    geom_line() +
    geom_abline(intercept = 0, slope = 1, linetype = 2, color = "red") +
    labs(
      title = paste0(model_name, " - Calibration plot (", imp_label, ")"),
      subtitle = paste0("Time = ", t0),
      x = paste0("Mean predicted risk at time ", t0),
      y = paste0("Observed risk at time ", t0)
    ) +
    theme_bw()
}

plot_time_roc <- function(roc_obj, model_name, imp_label = "Main") {
  roc_df_list <- list()
```

```
  for (i in seq_along(roc_obj$times)) {
    roc_df_list[[i]] <- data.frame(
      FP = roc_obj$FP[, i],
      TP = roc_obj$TP[, i],
      time = paste0("t=", roc_obj$times[i], " (AUC=", sprintf("%.3f", roc_obj$AUC[i]), ")")
    )
  }

  roc_df <- do.call(rbind, roc_df_list)

  ggplot(roc_df, aes(x = FP, y = TP, color = time)) +
    geom_line(linewidth = 1) +
    geom_abline(intercept = 0, slope = 1, linetype = 2, color = "grey50") +
    labs(
      title = paste0(model_name, " - Time-dependent ROC (", imp_label, ")"),
      x = "1 - Specificity",
      y = "Sensitivity",
      color = "Time"
    ) +
    theme_bw()
}

predict_pls_safe <- function(fit, newx) {
  out <- tryCatch(
    as.numeric(predict(fit, newdata = newx, type = "lp")),
    error = function(e) {
      as.numeric(predict(fit, newdata = newx, type = "risk"))
    }
  )
```

```
  out
}

##############################
## 5. Model-specific wrappers
##############################
fit_and_predict <- function(model_name, train_df, valid_df, predictors, params) {
  surv_formula <- make_surv_formula(predictors)

  if (model_name %in% c("CoxBoost", "xgboost", "plsRcox")) {
    xm <- build_xmat(train_df, valid_df, predictors)
    x_train <- xm$x_train
    x_valid <- xm$x_valid
  }

  if (model_name == "RSF") {
    fit <- randomForestSRC::rfsrc(
      surv_formula,
      data = train_df[, c("Time", "Surgery", predictors), drop = FALSE],
      ntree = params$ntree,
      nodesize = params$nodesize,
      mtry = params$mtry,
      nsplit = params$nsplit,
      splitrule = params$splitrule,
      importance = FALSE,
      proximity = FALSE,
      forest = TRUE
    )
```

```
    pred_train <- as.numeric(predict(fit, newdata = train_df[, c("Time", "Surgery", predictors), drop = FALSE])$predicted)
    pred_valid <- as.numeric(predict(fit, newdata = valid_df[, c("Time", "Surgery", predictors), drop = FALSE])$predicted)
  }

  else if (model_name == "GBM") {
    fit <- gbm::gbm(
      formula = surv_formula,
      data = train_df[, c("Time", "Surgery", predictors), drop = FALSE],
      distribution = "coxph",
      n.trees = params$n.trees,
      interaction.depth = params$interaction.depth,
      n.minobsinnode = params$n.minobsinnode,
      shrinkage = params$shrinkage,
      bag.fraction = 1,
      train.fraction = 1,
      cv.folds = 0,
      n.cores = 1,
      verbose = FALSE
    )

    pred_train <- as.numeric(predict(fit, newdata = train_df[, c("Time", "Surgery", predictors), drop = FALSE],
                                     n.trees = params$n.trees, type = "link"))
    pred_valid <- as.numeric(predict(fit, newdata = valid_df[, c("Time", "Surgery", predictors), drop = FALSE],
                                     n.trees = params$n.trees, type = "link"))
  }

  else if (model_name == "CoxBoost") {
```

```
    fit <- CoxBoost::CoxBoost(
      time = train_df$Time,
      status = train_df$Surgery,
      x = x_train,
      stepno = params$stepno,
      penalty = params$penalty
    )

    pred_train <- as.numeric(predict(fit, newdata = x_train, newtime = train_df$Time, newstatus = train_df$Surgery, type = "lp"))
    pred_valid <- as.numeric(predict(fit, newdata = x_valid, newtime = valid_df$Time, newstatus = valid_df$Surgery, type = "lp"))
  }

  else if (model_name == "survivalsvm") {
    fit <- survivalsvm::survivalsvm(
      formula = surv_formula,
      data = train_df[, c("Time", "Surgery", predictors), drop = FALSE],
      gamma.mu = params$gamma.mu,
      opt.meth = "quadprog",
      diff.meth = "makediff3",
      kernel = "lin_kernel",
      maxiter = 30,
      margin = params$margin,
      bound = params$bound
    )

    pred_train <- as.numeric(predict(fit, newdata = train_df[, c("Time", "Surgery", predictors), drop = FALSE])$predicted)
    pred_valid <- as.numeric(predict(fit, newdata = valid_df[, c("Time", "Surgery", predictors), drop = FALSE])$predicted)
```

```
}

else if (model_name == "xgboost") {
  y_train <- ifelse(train_df$Surgery == 1, train_df$Time, -train_df$Time)

  dtrain <- xgboost::xgb.DMatrix(data = x_train, label = y_train)
  dvalid <- xgboost::xgb.DMatrix(data = x_valid)

  xgb_param <- list(
    objective = "survival:cox",
    booster = "gbtree",
    eval_metric = "cox-nloglik",
    eta = params$eta,
    max_depth = params$max_depth,
    subsample = params$subsample,
    colsample_bytree = params$colsample_bytree,
    gamma = params$gamma
  )

  fit <- xgboost::xgb.train(
    params = xgb_param,
    data = dtrain,
    nrounds = params$nrounds,
    verbose = 0
  )

  pred_train <- as.numeric(predict(fit, newdata = dtrain))
  pred_valid <- as.numeric(predict(fit, newdata = dvalid))
}
```

```
    else if (model_name == "plsRcox") {
        fit <- plsRcox::plsRcox(
            Xplan = x_train,
            time = train_df$Time,
            event = train_df$Surgery,
            nt = params$nt
        )

        pred_train <- predict_pls_safe(fit, x_train)
        pred_valid <- predict_pls_safe(fit, x_valid)
    }

    else if (model_name == "CoxPH") {
        fit <- survival::coxph(
            surv_formula,
            data = train_df[, c("Time", "Surgery", predictors), drop = FALSE],
            x = TRUE,
            y = TRUE
        )

        pred_train <- as.numeric(predict(fit, newdata = train_df[, c("Time", "Surgery", predictors), drop
= FALSE], type = "lp"))
        pred_valid <- as.numeric(predict(fit, newdata = valid_df[, c("Time", "Surgery", predictors),
drop = FALSE], type = "lp"))
    }

    else {
        stop("Unsupported model_name: ", model_name)
    }
```

```
  list(train_score = pred_train, valid_score = pred_valid)
}

##############################
## 6. 5-fold CV within one dataset
##############################
cv_one_dataset <- function(dat, model_name, params, fold_id, predictors, eval_times) {
  n <- nrow(dat)

  oof_lp <- rep(NA_real_, n)
  oof_risk <- matrix(NA_real_, nrow = n, ncol = length(eval_times))
  colnames(oof_risk) <- paste0("risk_t", eval_times)

  fold_cindex <- rep(NA_real_, length(unique(fold_id)))

  for (k in sort(unique(fold_id))) {
    train_idx <- which(fold_id != k)
    valid_idx <- which(fold_id == k)

    tr <- dat[train_idx, c("Time", "Surgery", ".row_id", predictors), drop = FALSE]
    va <- dat[valid_idx, c("Time", "Surgery", ".row_id", predictors), drop = FALSE]

    pp <- preprocess_fold(tr, va, predictors)
    tr <- pp$train
    va <- pp$valid

    fp <- fit_and_predict(model_name, tr, va, predictors, params)
```

```
    rr <- rawscore_to_risk(
      train_time = tr$Time,
      train_status = tr$Surgery,
      train_score = fp$train_score,
      valid_score = fp$valid_score,
      times = eval_times
    )

    oof_lp[valid_idx] <- rr$lp_valid
    oof_risk[valid_idx, ] <- rr$risk_valid

    fold_cindex[k] <- get_cindex(va$Time, va$Surgery, rr$lp_valid)
  }

  list(
    mean_cindex = mean(fold_cindex, na.rm = TRUE),
    fold_cindex = fold_cindex,
    oof_lp = oof_lp,
    oof_risk = oof_risk
  )
}

##############################
## 7. Hyperparameter grids
##############################
p <- length(predictors)

model_grids <- list(
  RSF = unique(expand.grid(
```

```
    ntree = c(400),
    nodesize = c(15, 30),
    mtry = unique(pmax(1, c(floor(sqrt(p)), floor(p / 3)))),
    nsplit = c(3, 10),
    splitrule = "logrankscore",
    stringsAsFactors = FALSE
)),

GBM = expand.grid(
    n.trees = c(300, 600),
    interaction.depth = c(1, 3),
    n.minobsinnode = c(10, 30),
    shrinkage = c(0.01, 0.05),
    stringsAsFactors = FALSE
),

CoxBoost = expand.grid(
    stepno = c(50, 100, 150),
    penalty = c(50, 100),
    stringsAsFactors = FALSE
),

survivalsvm = expand.grid(
    gamma.mu = c(0.10, 0.25, 0.50),
    margin = c(0.05, 0.10),
    bound = c(10, 30),
    stringsAsFactors = FALSE
),
```

```
  xgboost = expand.grid(
    nrounds = c(100, 300),
    eta = c(0.05, 0.10),
    max_depth = c(1, 2),
    subsample = c(0.50, 0.80),
    colsample_bytree = c(0.60, 0.80),
    gamma = c(0, 1),
    stringsAsFactors = FALSE
  ),

  plsRcox = expand.grid(
    nt = c(2, 3, 4),
    stringsAsFactors = FALSE
  ),

  CoxPH = data.frame(
    dummy = 1,
    stringsAsFactors = FALSE
  )
)

############################
## 8. Main model function
############################
run_model_across_imputations <- function(model_name, imputed_list, fold_id, predictors, eval_times, grid_df) {
  stage1_best <- vector("list", length(imputed_list))
  stage1_tables <- vector("list", length(imputed_list))

  ## Stage 1: within each imputed dataset
```

```r
for (m in seq_along(imputed_list)) {
  cat("\n[", model_name, "] Stage 1 - dataset ", m, "/", length(imputed_list), "\n", sep = "")

  grid_scores <- rep(NA_real_, nrow(grid_df))

  for (g in seq_len(nrow(grid_df))) {
    params <- as.list(grid_df[g, , drop = FALSE])

    cv_res <- cv_one_dataset(
      dat = imputed_list[[m]],
      model_name = model_name,
      params = params,
      fold_id = fold_id,
      predictors = predictors,
      eval_times = eval_times
    )

    grid_scores[g] <- cv_res$mean_cindex
  }

  tb <- cbind(
    dataset = m,
    grid_id = seq_len(nrow(grid_df)),
    grid_df,
    mean_cindex = grid_scores
  )

  best_idx <- which.max(grid_scores)
```

```
  stage1_tables[[m]] <- as.data.frame(tb)
  stage1_best[[m]] <- grid_df[best_idx, , drop = FALSE]
}

candidate_df <- unique(do.call(rbind, stage1_best))
rownames(candidate_df) <- NULL

## Stage 2: evaluate stage-1 best candidates across all imputations
stage2_records <- list()
rec_i <- 1

for (cand_id in seq_len(nrow(candidate_df))) {
  params <- as.list(candidate_df[cand_id, , drop = FALSE])

  for (m in seq_along(imputed_list)) {
    cat("\n[", model_name, "] Stage 2 - candidate ", cand_id, "/", nrow(candidate_df),
        ", dataset ", m, "/", length(imputed_list), "\n", sep = "")

    cv_res <- cv_one_dataset(
      dat = imputed_list[[m]],
      model_name = model_name,
      params = params,
      fold_id = fold_id,
      predictors = predictors,
      eval_times = eval_times
    )

    stage2_records[[rec_i]] <- cbind(
      candidate_id = cand_id,
```

```
          dataset = m,
          candidate_df[cand_id, , drop = FALSE],
          mean_cindex = cv_res$mean_cindex
        )
        rec_i <- rec_i + 1
    }
}

stage2_table <- do.call(rbind, stage2_records)
stage2_mean <- aggregate(mean_cindex ~ candidate_id, data = stage2_table, FUN = mean)

best_cand_id <- stage2_mean$candidate_id[which.max(stage2_mean$mean_cindex)]
final_params <- as.list(candidate_df[best_cand_id, , drop = FALSE])

## Final OOF predictions using final hyperparameters
final_cv_list <- vector("list", length(imputed_list))

for (m in seq_along(imputed_list)) {
    cat("\n[", model_name, "] Final OOF - dataset ", m, "/", length(imputed_list), "\n", sep = "")

    final_cv_list[[m]] <- cv_one_dataset(
        dat = imputed_list[[m]],
        model_name = model_name,
        params = final_params,
        fold_id = fold_id,
        predictors = predictors,
        eval_times = eval_times
    )
}
```

```r
## Averaged individual-level OOF prediction across imputations
n <- nrow(imputed_list[[1]])
m <- length(imputed_list)

oof_lp_mat <- matrix(NA_real_, nrow = n, ncol = m)
oof_risk_arr <- array(NA_real_, dim = c(n, length(eval_times), m))

for (i in seq_along(final_cv_list)) {
  oof_lp_mat[, i] <- final_cv_list[[i]]$oof_lp
  oof_risk_arr[, , i] <- final_cv_list[[i]]$oof_risk
}

avg_oof_lp <- rowMeans(oof_lp_mat, na.rm = TRUE)
avg_oof_risk <- apply(oof_risk_arr, c(1, 2), mean, na.rm = TRUE)

main_eval_df <- imputed_list[[1]][, c(".row_id", "Time", "Surgery"), drop = FALSE]
main_eval_df$avg_oof_lp <- avg_oof_lp

for (j in seq_along(eval_times)) {
  main_eval_df[[paste0("avg_risk_t", eval_times[j])]] <- avg_oof_risk[, j]
}

## Main metrics
main_cindex <- get_cindex(main_eval_df$Time, main_eval_df$Surgery, main_eval_df$avg_oof_lp)
main_auc <- calc_time_auc(main_eval_df$Time, main_eval_df$Surgery, main_eval_df$avg_oof_lp, eval_times)

main_brier <- data.frame(
```

```r
  time = eval_times,
  Brier = sapply(seq_along(eval_times), function(j) {
    calc_brier_ipcw(
      time = main_eval_df$Time,
      status = main_eval_df$Surgery,
      pred_risk = main_eval_df[[paste0("avg_risk_t", eval_times[j])]],
      t0 = eval_times[j]
    )
  })
)

## Per-dataset metrics
imp_cindex <- rep(NA_real_, length(final_cv_list))
imp_auc_mat <- matrix(NA_real_, nrow = length(final_cv_list), ncol = length(eval_times))
imp_brier_mat <- matrix(NA_real_, nrow = length(final_cv_list), ncol = length(eval_times))

for (i in seq_along(final_cv_list)) {
  dd <- imputed_list[[i]]
  rr <- final_cv_list[[i]]

  imp_cindex[i] <- get_cindex(dd$Time, dd$Surgery, rr$oof_lp)

  auc_tmp <- calc_time_auc(dd$Time, dd$Surgery, rr$oof_lp, eval_times)
  imp_auc_mat[i, ] <- auc_tmp$AUC

  imp_brier_mat[i, ] <- sapply(seq_along(eval_times), function(j) {
    calc_brier_ipcw(
      time = dd$Time,
      status = dd$Surgery,
```

```
      pred_risk = rr$oof_risk[, j],
      t0 = eval_times[j]
    )
  })
}

imp_summary <- list(
  cindex_each = data.frame(dataset = seq_along(imp_cindex), C_index = imp_cindex),
  cindex_summary = data.frame(
    mean = mean(imp_cindex, na.rm = TRUE),
    sd = sd(imp_cindex, na.rm = TRUE)
  ),
  auc_each = data.frame(
    dataset = rep(seq_len(nrow(imp_auc_mat)), each = length(eval_times)),
    time = rep(eval_times, times = nrow(imp_auc_mat)),
    AUC = as.vector(t(imp_auc_mat))
  ),
  auc_summary = data.frame(
    time = eval_times,
    mean = colMeans(imp_auc_mat, na.rm = TRUE),
    sd = apply(imp_auc_mat, 2, sd, na.rm = TRUE)
  ),
  brier_each = data.frame(
    dataset = rep(seq_len(nrow(imp_brier_mat)), each = length(eval_times)),
    time = rep(eval_times, times = nrow(imp_brier_mat)),
    Brier = as.vector(t(imp_brier_mat))
  ),
  brier_summary = data.frame(
    time = eval_times,
```

```
      mean = colMeans(imp_brier_mat, na.rm = TRUE),
      sd = apply(imp_brier_mat, 2, sd, na.rm = TRUE)
    )
  )

  list(
    model_name = model_name,
    final_params = final_params,
    stage1_tables = stage1_tables,
    stage2_table = stage2_table,
    stage2_mean = stage2_mean,
    final_cv_list = final_cv_list,
    main_eval_df = main_eval_df,
    main_metrics = list(
      cindex = main_cindex,
      auc = main_auc,
      brier = main_brier
    ),
    imp_summary = imp_summary
  )
}

############################
## 9. Plot builders for per-dataset results
############################
build_per_dataset_calibration <- function(model_result, imputed_list, eval_time, eval_times) {
  per_dataset_calibration <- vector("list", length(imputed_list))

  for (i in seq_along(imputed_list)) {
```

```r
    dd <- imputed_list[[i]]
    rr <- model_result$final_cv_list[[i]]

    time_idx <- which(eval_times == eval_time)
    if (length(time_idx) != 1) stop("eval_time 不在 eval_times 中。")

    cal_df_i <- make_calibration_df(
      time = dd$Time,
      status = dd$Surgery,
      pred_risk = rr$oof_risk[, time_idx],
      t0 = eval_time,
      n_groups = 10
    )

    p_i <- plot_calibration_curve(
      cal_df = cal_df_i,
      model_name = model_result$model_name,
      t0 = eval_time,
      imp_label = paste0("Dataset ", i)
    )

    per_dataset_calibration[[i]] <- list(
      calibration_df = cal_df_i,
      plot = p_i
    )
  }

  per_dataset_calibration
}
```

```
}

build_per_dataset_dca <- function(model_result, imputed_list, eval_time, eval_times) {
  per_dataset_dca <- vector("list", length(imputed_list))

  for (i in seq_along(imputed_list)) {
    dd <- imputed_list[[i]]
    rr <- model_result$final_cv_list[[i]]

    time_idx <- which(eval_times == eval_time)
    if (length(time_idx) != 1) stop("eval_time 不在 eval_times 中。")

    dca_df_i <- data.frame(
      Time = dd$Time,
      Surgery = dd$Surgery,
      pred_risk = rr$oof_risk[, time_idx]
    )

    dca_obj_i <- dcurves::dca(
      formula = Surv(Time, Surgery) ~ pred_risk,
      data = dca_df_i,
      time = eval_time
    )

    per_dataset_dca[[i]] <- list(
      data = dca_df_i,
      dca = dca_obj_i
    )
  }
```

```
  per_dataset_dca
}

build_per_dataset_roc <- function(model_result, imputed_list, eval_times) {
  per_dataset_roc <- vector("list", length(imputed_list))

  for (i in seq_along(imputed_list)) {
    dd <- imputed_list[[i]]
    rr <- model_result$final_cv_list[[i]]

    roc_obj_i <- calc_time_roc_object(
      time = dd$Time,
      status = dd$Surgery,
      marker = rr$oof_lp,
      times = eval_times
    )

    p_i <- plot_time_roc(
      roc_obj = roc_obj_i,
      model_name = model_result$model_name,
      imp_label = paste0("Dataset ", i)
    )

    per_dataset_roc[[i]] <- list(
      roc = roc_obj_i,
      plot = p_i
    )
  }
```

```
  per_dataset_roc
}

save_per_dataset_plots <- function(per_dataset_calibration, per_dataset_dca, per_dataset_roc, outdir)
{
  if (!dir.exists(outdir)) dir.create(outdir, recursive = TRUE)

  for (i in seq_along(per_dataset_calibration)) {
    ggsave(
      filename = file.path(outdir, paste0("Calibration_Dataset_", i, ".png")),
      plot = per_dataset_calibration[[i]]$plot,
      width = 6,
      height = 5,
      dpi = 300
    )
  }

  for (i in seq_along(per_dataset_roc)) {
    ggsave(
      filename = file.path(outdir, paste0("ROC_Dataset_", i, ".png")),
      plot = per_dataset_roc[[i]]$plot,
      width = 6,
      height = 5,
      dpi = 300
    )
  }

  for (i in seq_along(per_dataset_dca)) {
    png(
      filename = file.path(outdir, paste0("DCA_Dataset_", i, ".png")),
```

```
            width = 1800,
            height = 1500,
            res = 300
        )
        plot(
            per_dataset_dca[[i]]$dca,
            main = paste0("Decision Curve Analysis - Dataset ", i)
        )
        dev.off()
    }
}

##############################
## 10. Run all models
##############################
model_names <- names(model_grids)

all_results <- lapply(model_names, function(mn) {
    run_model_across_imputations(
        model_name = mn,
        imputed_list = imputed_list,
        fold_id = fold_id,
        predictors = predictors,
        eval_times = eval_times,
        grid_df = model_grids[[mn]]
    )
})
names(all_results) <- model_names
```

```
##############################
## 11. Rank models by main C-index
##############################
model_rank <- do.call(rbind, lapply(names(all_results), function(mn) {
  data.frame(
    Model = mn,
    C_index = all_results[[mn]]$main_metrics$cindex,
    stringsAsFactors = FALSE
  )
}))

model_rank <- model_rank[order(-model_rank$C_index), , drop = FALSE]
print(model_rank)

best_model <- model_rank$Model[1]
best_res <- all_results[[best_model]]

cat("\nBest model based on averaged individual-level OOF C-index: ", best_model, "\n", sep = "")
cat("\nFinal parameters of best model:\n")
print(best_res$final_params)

##############################
## 12. Main results for best model
##############################
cat("\nMain C-index:\n")
print(best_res$main_metrics$cindex)

cat("\nMain time-dependent AUC:\n")
print(best_res$main_metrics$auc)
```

```
cat("\nMain Brier score:\n")
print(best_res$main_metrics$brier)

cat("\nPer-dataset summary:\n")
print(best_res$imp_summary$cindex_summary)
print(best_res$imp_summary$auc_summary)
print(best_res$imp_summary$brier_summary)

##############################
## 13. Main ROC plot
##############################
main_roc_obj <- calc_time_roc_object(
  time = best_res$main_eval_df$Time,
  status = best_res$main_eval_df$Surgery,
  marker = best_res$main_eval_df$avg_oof_lp,
  times = eval_times
)

p_main_roc <- plot_time_roc(
  roc_obj = main_roc_obj,
  model_name = best_model,
  imp_label = "Main averaged OOF prediction"
)

print(p_main_roc)

##############################
## 14. Main calibration plot
```

```
##############################
risk_col <- paste0("avg_risk_t", dca_time)

if (!risk_col %in% names(best_res$main_eval_df)) {
  stop("在 main_eval_df 中未找到主分析风险列： ", risk_col)
}

cal_df_main <- make_calibration_df(
  time = best_res$main_eval_df$Time,
  status = best_res$main_eval_df$Surgery,
  pred_risk = best_res$main_eval_df[[risk_col]],
  t0 = dca_time,
  n_groups = 10
)

p_cal_main <- plot_calibration_curve(
  cal_df = cal_df_main,
  model_name = best_model,
  t0 = dca_time,
  imp_label = "Main averaged OOF prediction"
)

print(p_cal_main)

##############################
## 15. Main DCA
##############################
dca_formula <- as.formula(paste0("Surv(Time, Surgery) ~ ", risk_col))
```

```
dca_obj_main <- dcurves::dca(
  formula = dca_formula,
  data = best_res$main_eval_df,
  time = dca_time
)

plot(dca_obj_main)

##############################
## 16. Per-dataset plots
##############################
per_dataset_calibration <- build_per_dataset_calibration(
  model_result = best_res,
  imputed_list = imputed_list,
  eval_time = dca_time,
  eval_times = eval_times
)

per_dataset_dca <- build_per_dataset_dca(
  model_result = best_res,
  imputed_list = imputed_list,
  eval_time = dca_time,
  eval_times = eval_times
)

per_dataset_roc <- build_per_dataset_roc(
  model_result = best_res,
  imputed_list = imputed_list,
  eval_times = eval_times
```

```r
)

## 控制台显示
for (i in seq_along(per_dataset_roc)) {
  print(per_dataset_roc[[i]]$plot)
}
for (i in seq_along(per_dataset_calibration)) {
  print(per_dataset_calibration[[i]]$plot)
}
for (i in seq_along(per_dataset_dca)) {
  plot(
    per_dataset_dca[[i]]$dca,
    main = paste0(best_model, " - DCA (Dataset ", i, ")")
  )
}

############################
## 17. Save plots
############################
ggsave(
  filename = file.path(output_dir, paste0(best_model, "_Main_ROC.png")),
  plot = p_main_roc,
  width = 6,
  height = 5,
  dpi = 300
)

ggsave(
  filename = file.path(output_dir, paste0(best_model, "_Main_Calibration.png")),
```

```
  plot = p_cal_main,
  width = 6,
  height = 5,
  dpi = 300
)

png(
  filename = file.path(output_dir, paste0(best_model, "_Main_DCA.png")),
  width = 1800,
  height = 1500,
  res = 300
)
plot(dca_obj_main, main = paste0(best_model, " - Main DCA"))
dev.off()

save_per_dataset_plots(
  per_dataset_calibration = per_dataset_calibration,
  per_dataset_dca = per_dataset_dca,
  per_dataset_roc = per_dataset_roc,
  outdir = file.path(output_dir, paste0(best_model, "_Per_Dataset_Plots"))
)

##############################
## 18. Export tables to Excel
##############################
out_list <- list(
  model_rank = model_rank,
  best_model_main_auc = best_res$main_metrics$auc,
  best_model_main_brier = best_res$main_metrics$brier,
```

```r
  best_model_cindex_each_dataset = best_res$imp_summary$cindex_each,
  best_model_auc_each_dataset = best_res$imp_summary$auc_each,
  best_model_brier_each_dataset = best_res$imp_summary$brier_each,
  best_model_auc_summary = best_res$imp_summary$auc_summary,
  best_model_brier_summary = best_res$imp_summary$brier_summary,
  best_model_stage2_mean = best_res$stage2_mean
)

## 每个模型的主结果也导出
for (mn in names(all_results)) {
  out_list[[paste0(mn, "_main_auc")]] <- all_results[[mn]]$main_metrics$auc
  out_list[[paste0(mn, "_main_brier")]] <- all_results[[mn]]$main_metrics$brier
  out_list[[paste0(mn, "_stage2_mean")]] <- all_results[[mn]]$stage2_mean
}

## 最佳模型每个数据集的校准数据导出
for (i in seq_along(per_dataset_calibration)) {
  out_list[[paste0(best_model, "_Calibration_Dataset_", i)]] <- per_dataset_calibration[[i]]$calibration_df
}

writexl::write_xlsx(
  out_list,
  path = file.path(output_dir, "internal_validation_5fold_results.xlsx")
)

##############################
## 19. Save R objects
```

```
##############################
saveRDS(
  list(
    all_results = all_results,
    best_model = best_model,
    best_res = best_res,
    main_roc_obj = main_roc_obj,
    per_dataset_roc = per_dataset_roc,
    per_dataset_calibration = per_dataset_calibration,
    per_dataset_dca = per_dataset_dca
  ),
  file = file.path(output_dir, "internal_validation_results.rds")
)


cat("\n 全部运行完成。\n")

cat("输出目录：", normalizePath(output_dir), "\n")
```

------------------------Python---

########SHAP---

```
import pandas as pd
import numpy as np
import shap
import matplotlib.pyplot as plt
from sksurv.ensemble import GradientBoostingSurvivalAnalysis
# =======================
# 1. Data Loading and Preprocessing
```

```
# ========================
df = pd.read_excel("C:/Users/32121/Desktop/ /train.xlsx", sheet_name="Sheet2")
# Convert to survival analysis format
y_structured = np.array(
[(event, time) for event, time in zip(df["Surgery"], df["Time"])],
dtype=[("event", "?"), ("time", "<f8")]
)
# Extract feature matrix (automatically excluding Time and Surgery columns)
X = df.drop(["Time", "Surgery"], axis=1)
# ========================
# 2. Construct GBM Survival Model
# ========================
gbm_model = GradientBoostingSurvivalAnalysis(
n_estimators=500,
max_depth=3,
learning_rate=0.02,
min_samples_leaf=35,
loss="coxph",
random_state=42,
verbose=1
)
gbm_model.fit(X, y_structured)
# ========================
# 3. SHAP Analysis (using GBM Link)
# ========================
def predict_link(X):
return gbm_model.predict(X)
```

```
background_data = shap.sample(X, 740)explainer = shap.KernelExplainer(
model=predict_link,
data=background_data,
link="identity"
)
sample_data = X.iloc[:740]
shap_values = explainer.shap_values(sample_data)
# ======================
# 4. Visualization (using Waterfall Plot)
# ======================
# Wrap SHAP values as SHAP Explanation object
shap_values_explanation = shap.Explanation(values=shap_values[10],
base_values=explainer.expected_value,
data=sample_data.iloc[10, :],
feature_names=X.columns.tolist())
# Use SHAP values of a single sample
plt.figure(figsize=(18, 10))
shap.waterfall_plot(shap_values_explanation, max_display=15)
plt.title("Sample SHAP Contribution Breakdown (Waterfall Plot)")
plt.tight_layout()
# ======================
# 5. Visualization (using Force Plot instead of Waterfall Plot)
# ======================
plt.figure(figsize=(18, 10))
shap.force_plot(
explainer.expected_value,
shap_values[1, :], # Choose SHAP values of the second sample
```

```
sample_data.iloc[1, :], # Choose feature data of the second sample
feature_names=X.columns.tolist(),
matplotlib=True
)
plt.title("Sample SHAP Contribution Breakdown (Force Plot)")
plt.tight_layout()
# ======================
# 6. Global Feature Importance (Beehive Plot)
# ======================
# Custom color map (purple to yellow)
from matplotlib.colors import LinearSegmentedColormap
# Create a gradient from purple to yellow
cmap = LinearSegmentedColormap.from_list("purple_yellow", ["purple", "yellow"])
# Plot Beehive chart with custom color scheme
plt.figure(figsize=(10, 6))shap.summary_plot(shap_values, sample_data, plot_type="dot",
color=cmap)
plt.title("GBM Feature Importance (Beehive Plot)")
plt.tight_layout()
# ======================
# 7. Global Feature Importance (Bar Plot)
# ======================
plt.figure(figsize=(10, 6))
shap.summary_plot(shap_values, sample_data, plot_type="bar")
plt.title("GBM Feature Importance (Bar Plot)")
plt.tight_layout()
# ======================
# 8. Feature Dependency Plots
```

```
# =========================
for col in X.columns[:6]: # Show the first feature
plt.figure()
shap.dependence_plot(
col,
shap_values,
sample_data,
interaction_index=None
)
plt.title(f"'{col}' Feature Dependency")
plt.tight_layout()
# Show all plots
plt.show()
```
