# Supplementary material for: Integrating inflammatory and coagulation biomarkers for surgical risk stratification and treatment benefit assessment in Crohn’s disease
Source: Front Immunol. 2026 May 7;17:1657279. doi: 10.3389/fimmu.2026.1657279 (PMC13189891; doi:10.3389/fimmu.2026.1657279)

Supplementary Figure 1. Surgery-free survival in the overall study population

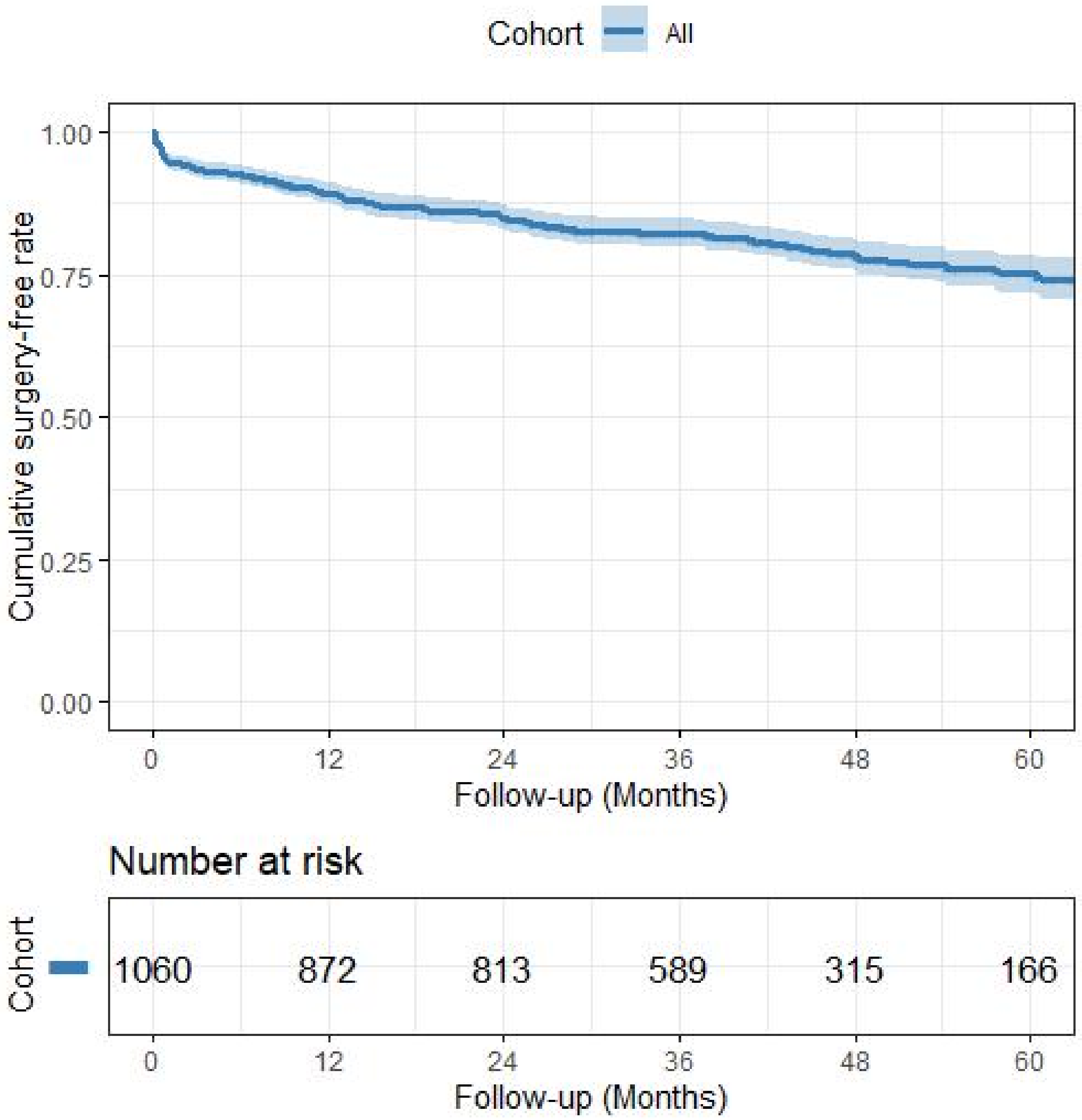

Supplementary Figure 2. Heatmaps of correlations among all candidate predictors in the five imputed datasets. Panels A–E represent imputed datasets 1–5, respectively.

A

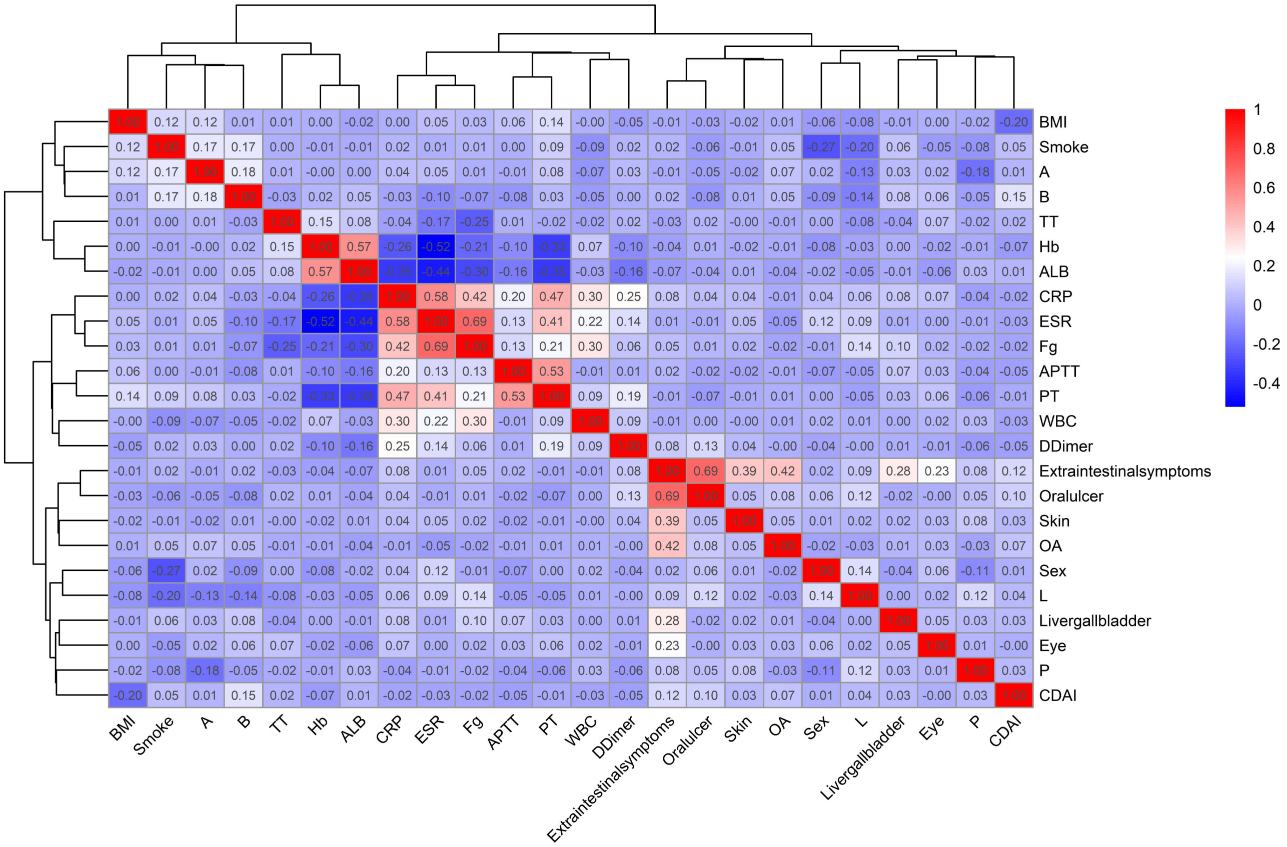

B

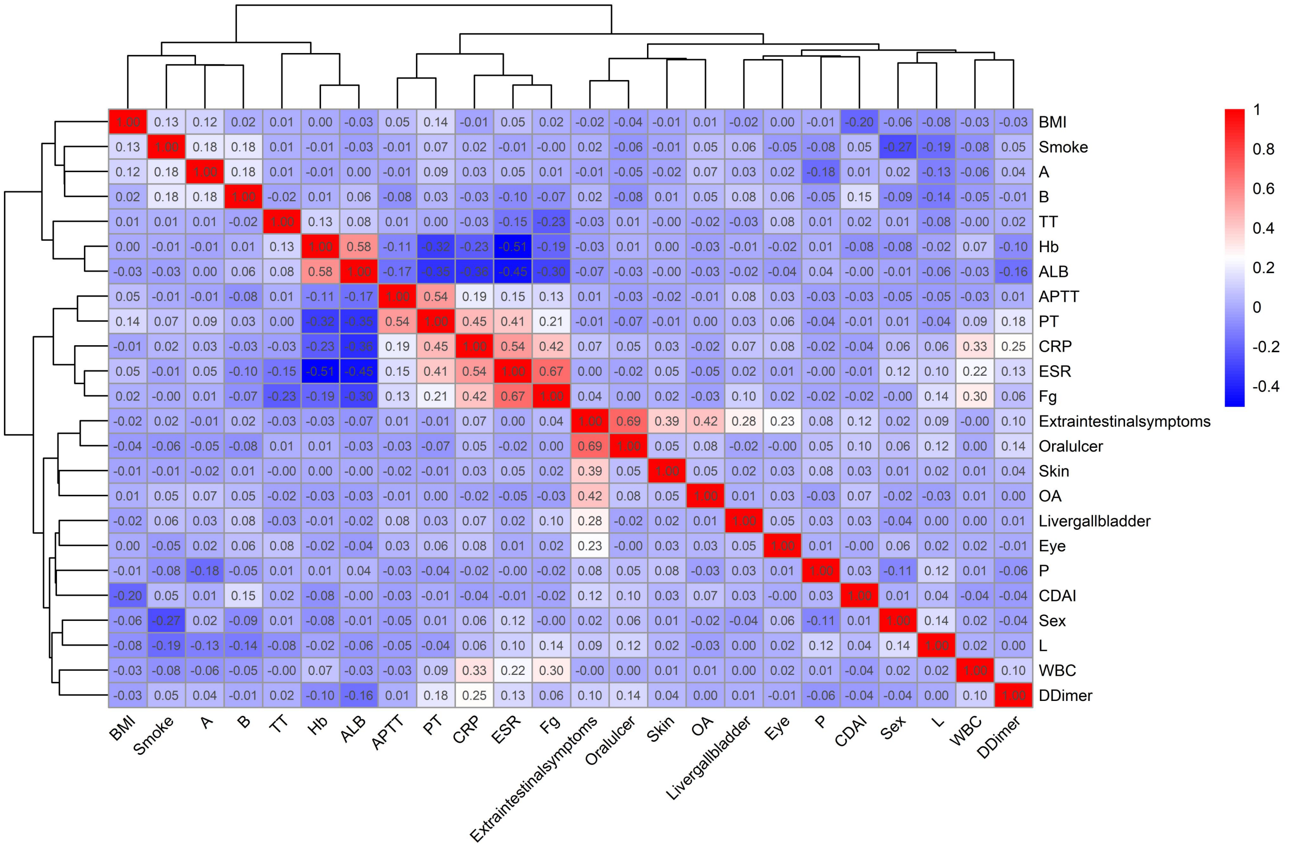
BMI
Smoke
A
B
TT
Hb
ALB
APTT
PT
CRP
ESR
Fg
Extraintestinalsymptoms
Oralulcer
Skin
OA
Livergallbladder
Eye
P
CDAI
Sex
L
WBC
DDimer
1
0.8
0.6
0.4
0.2
0
-0.2
-0.4

C

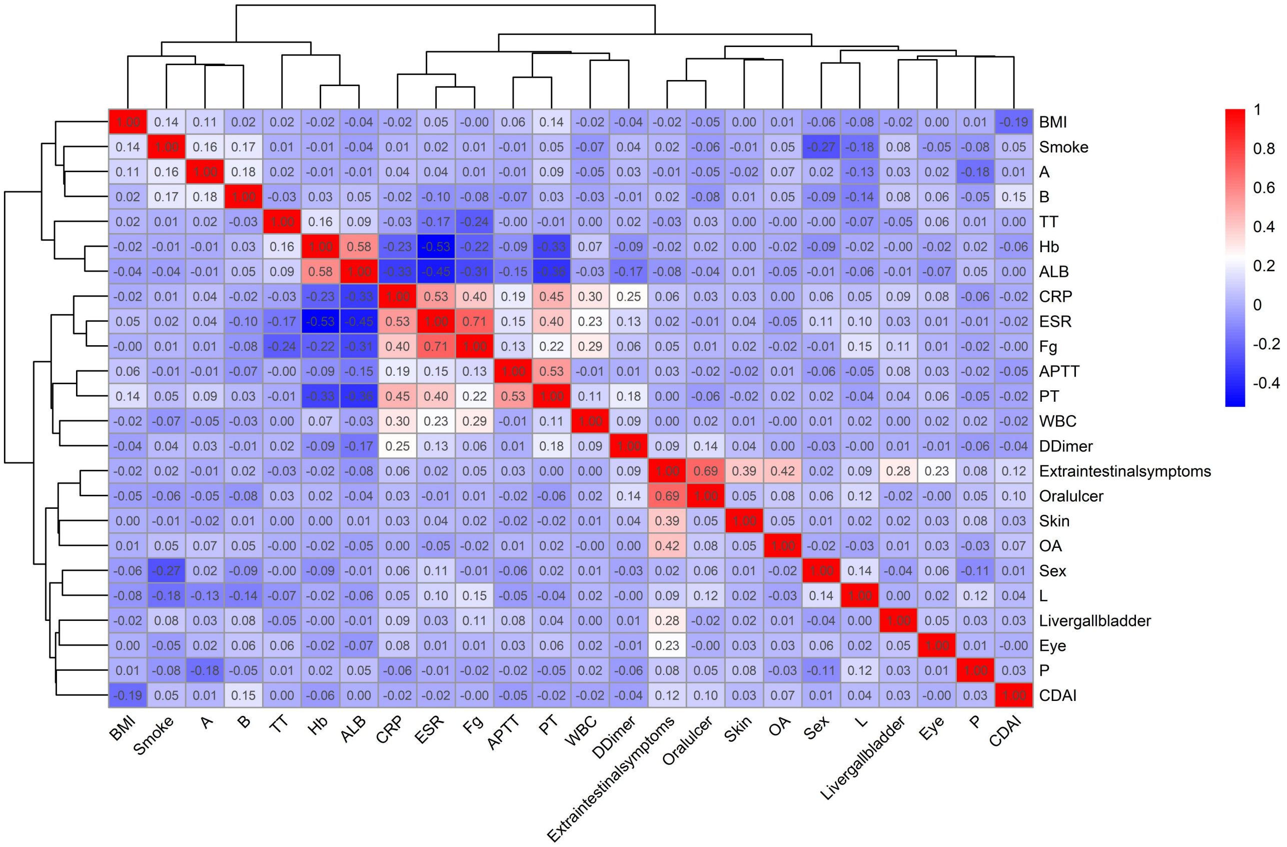
BMI
Smoke
A
B
TT
Hb
ALB
CRP
ESR
Fg
APTT
PT
WBC
DDimer
Extraintestinalsymptoms
Oralulcer
Skin
OA
Sex
L
Livergallbladder
Eye
P
CDAI
1
0.8
0.6
0.4
0.2
0
-0.2
-0.4

D

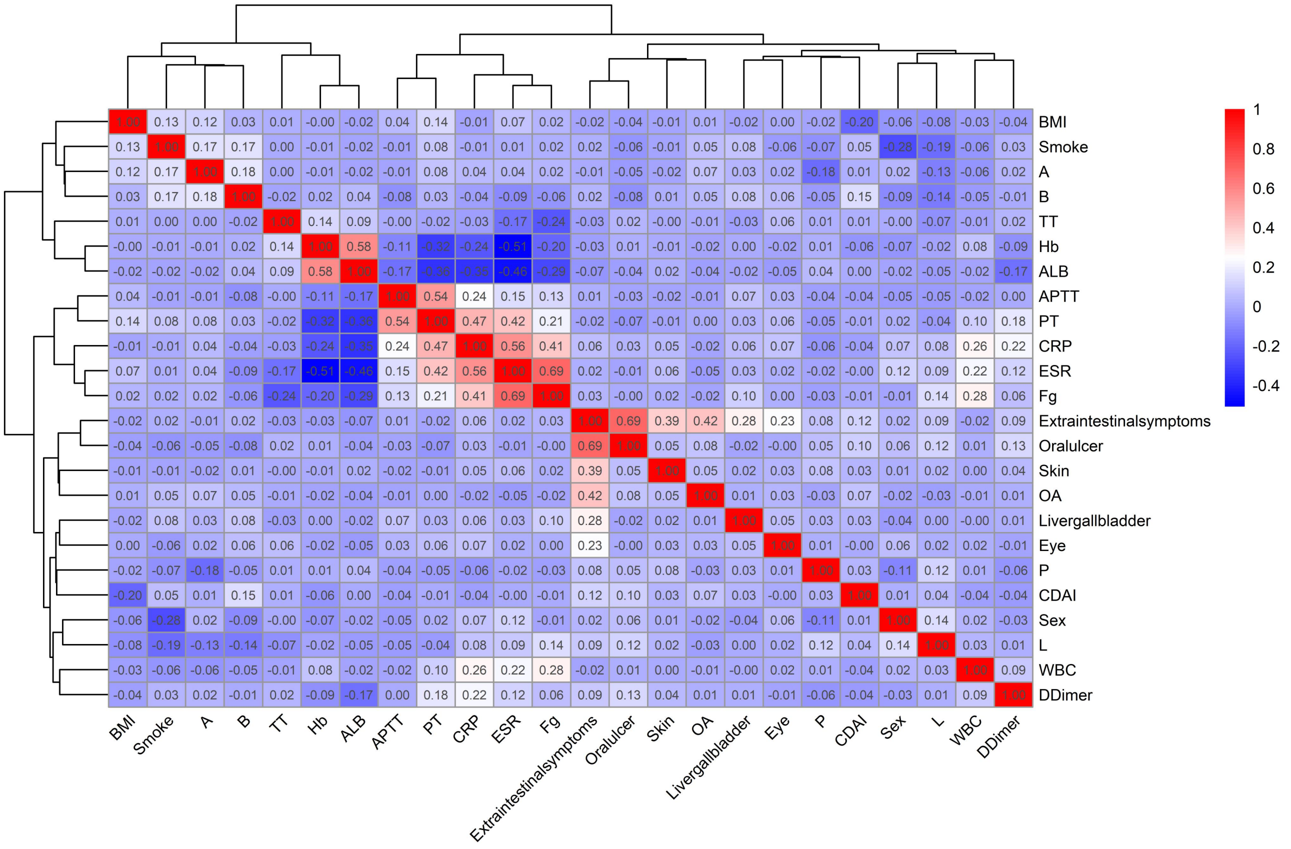
BMI
Smoke
A
B
TT
Hb
ALB
APTT
PT
CRP
ESR
Fg
Extraintestinalsymptoms
Oralulcer
Skin
OA
Livergallbladder
Eye
P
CDAI
Sex
L
WBC
DDimer
1
0.8
0.6
0.4
0.2
0
-0.2
-0.4

E

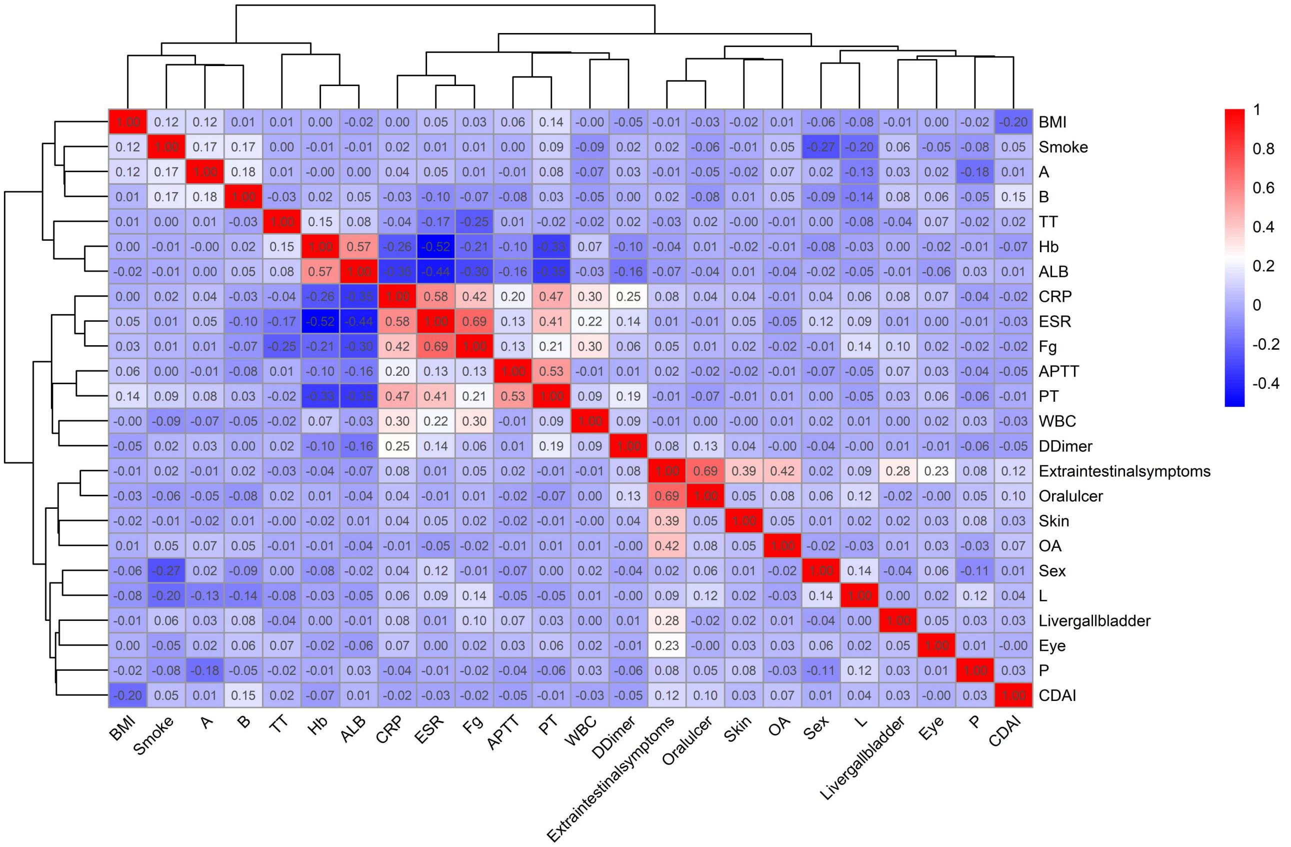
BMI
Smoke
A
B
TT
Hb
ALB
CRP
ESR
Fg
APTT
PT
WBC
DDimer
Extraintestinalsymptoms
Oralulcer
Skin
OA
Sex
L
Livergallbladder
Eye
P
CDAI
1
0.8
0.6
0.4
0.2
0
-0.2
-0.4

Supplementary Figure 3. Identification of candidate predictors using least absolute shrinkage and selection operator (LASSO) regression and the Boruta algorithm in imputed dataset 2.

(A) LASSO coefficient profiles of candidate predictors, illustrating the shrinkage of regression coefficients as the penalty parameter λchanges. The x-axis represents $-\log(\lambda)$, the y-axis represents the coefficient values, and the numbers above indicate the number of nonzero predictors retained in the model.

(B) Cross-validation plot for selection of the optimal penalty parameter λin the LASSO model. Red dots indicate the partial likelihood deviance at different λvalues, gray error bars indicate the standard errors, and the dotted vertical line marks the optimal λ.

(C) Results of candidate predictor selection using the Boruta algorithm. Green, red, and blue box plots denote confirmed, rejected, and tentative predictors, respectively.

(D) Venn diagram showing the overlap between predictors selected by LASSO and Boruta. LASSO identified 19 predictors and Boruta identified 12 predictors, with 9 predictors shared by both methods.

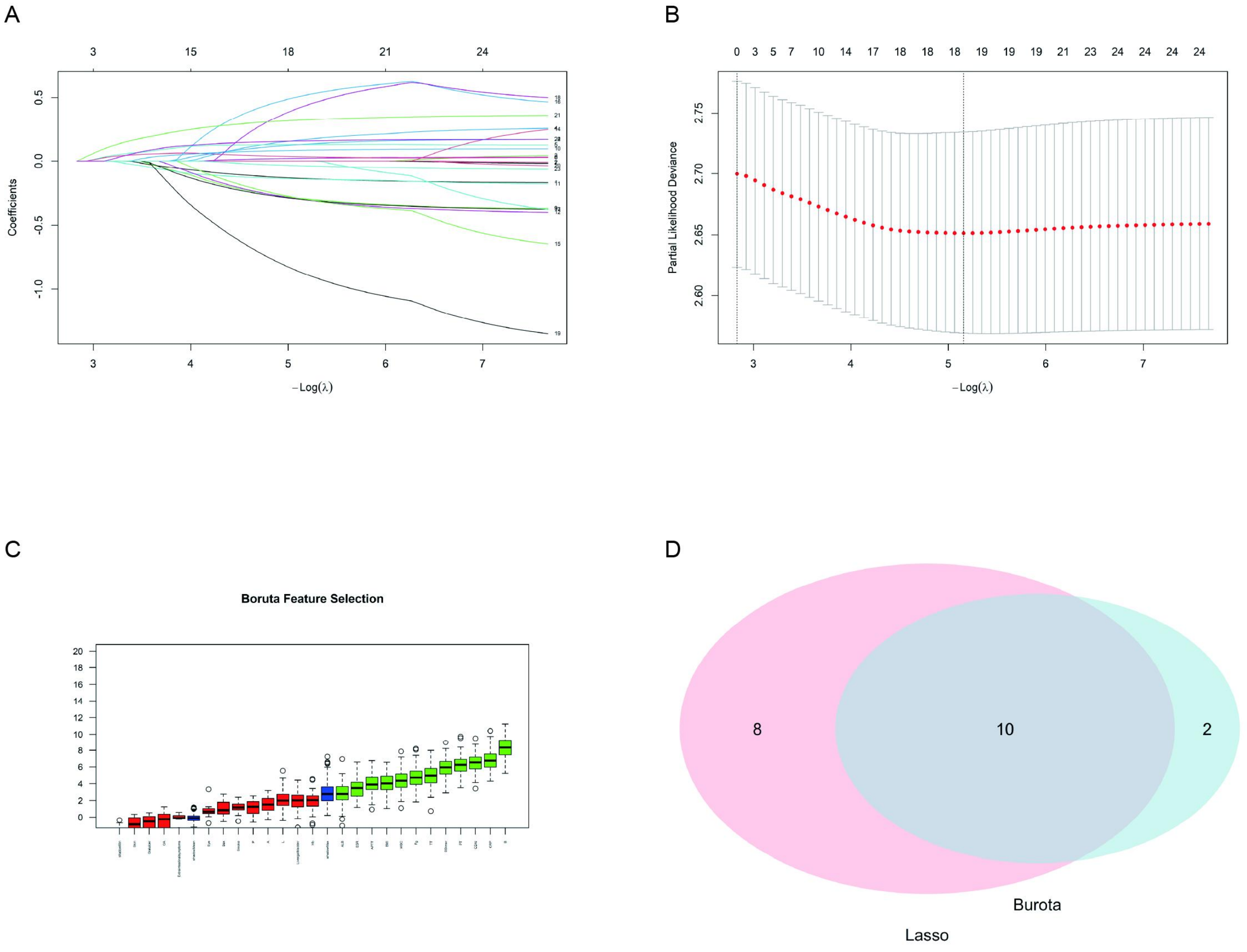

Supplementary Figure 4. Identification of candidate predictors using least absolute shrinkage and selection operator (LASSO) regression and the Boruta algorithm in imputed dataset 3.

(A) LASSO coefficient profiles of candidate predictors, illustrating the shrinkage of regression coefficients as the penalty parameter λ changes. The x-axis represents $-\log(\lambda)$, the y-axis represents the coefficient values, and the numbers above indicate the number of nonzero predictors retained in the model.

(B) Cross-validation plot for selection of the optimal penalty parameter λ in the LASSO model. Red dots indicate the partial likelihood deviance at different λ values, gray error bars indicate the standard errors, and the dotted vertical line marks the optimal λ.

(C) Results of candidate predictor selection using the Boruta algorithm. Green, red, and blue box plots denote confirmed, rejected, and tentative predictors, respectively.

(D) Venn diagram showing the overlap between predictors selected by LASSO and Boruta. LASSO identified 19 predictors and Boruta identified 12 predictors, with 9 predictors shared by both methods.

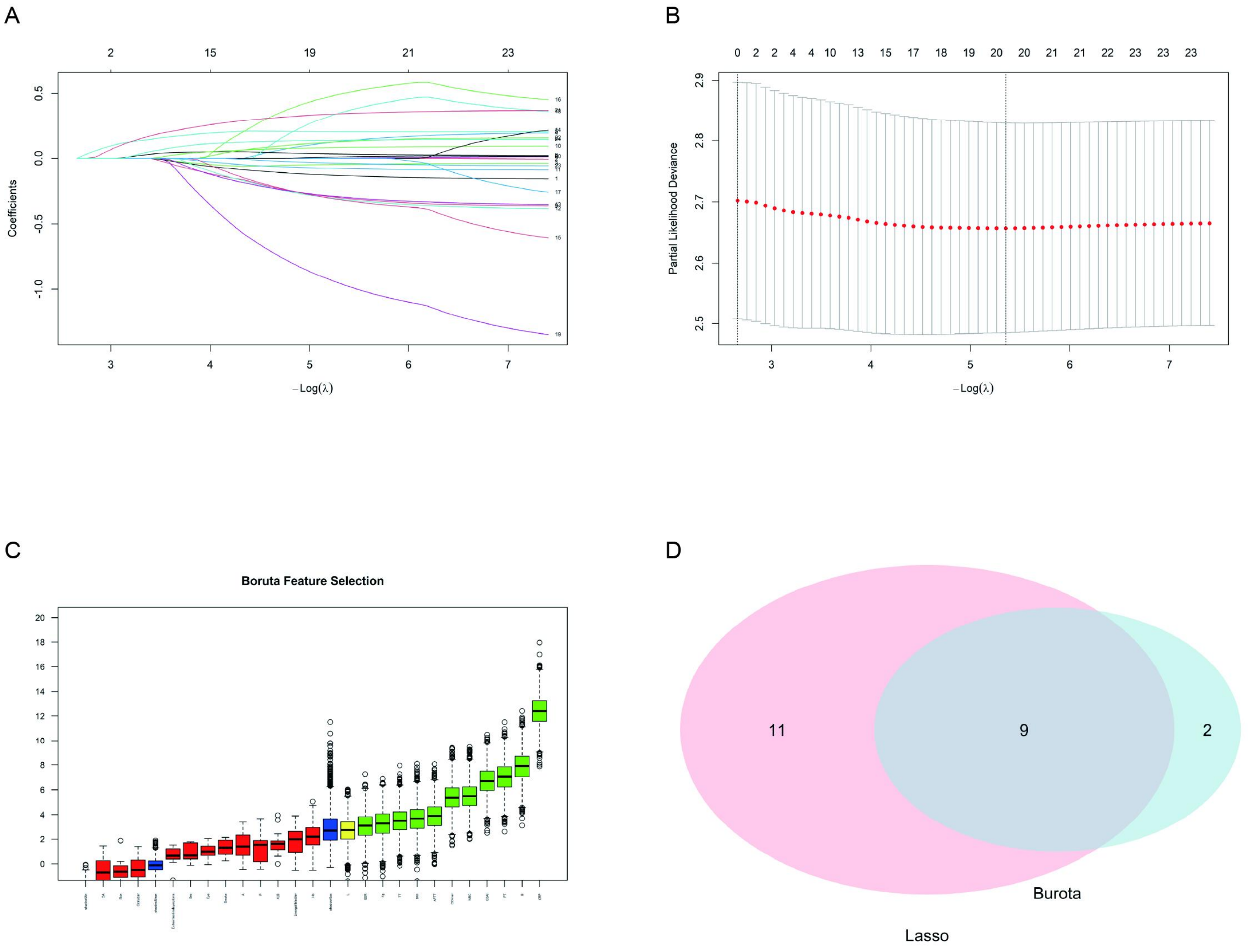

Supplementary Figure 5. Identification of candidate predictors using least absolute shrinkage and selection operator (LASSO) regression and the Boruta algorithm in imputed dataset 4.

(A) LASSO coefficient profiles of candidate predictors, illustrating the shrinkage of regression coefficients as the penalty parameter λchanges. The x-axis represents $-\log(\lambda)$, the y-axis represents the coefficient values, and the numbers above indicate the number of nonzero predictors retained in the model.

(B) Cross-validation plot for selection of the optimal penalty parameter λin the LASSO model. Red dots indicate the partial likelihood deviance at different λvalues, gray error bars indicate the standard errors, and the dotted vertical line marks the optimal λ.

(C) Results of candidate predictor selection using the Boruta algorithm. Green, red, and blue box plots denote confirmed, rejected, and tentative predictors, respectively.

(D) Venn diagram showing the overlap between predictors selected by LASSO and Boruta. LASSO identified 19 predictors and Boruta identified 12 predictors, with 9 predictors shared by both methods.

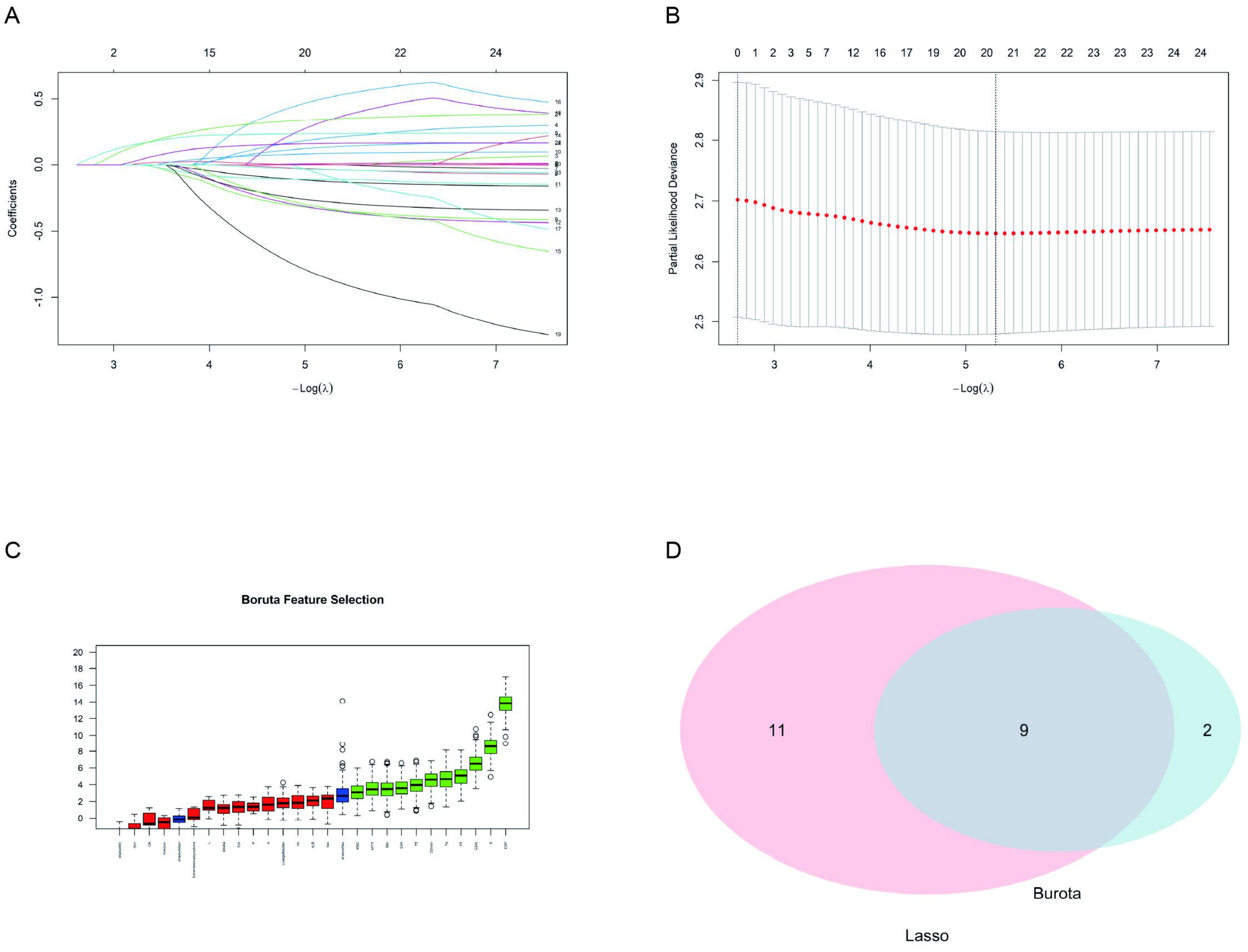

Supplementary Figure 6. Identification of candidate predictors using least absolute shrinkage and selection operator (LASSO) regression and the Boruta algorithm in imputed dataset 5.

(A) LASSO coefficient profiles of candidate predictors, illustrating the shrinkage of regression coefficients as the penalty parameter λ changes. The x-axis represents $-\log(\lambda)$, the y-axis represents the coefficient values, and the numbers above indicate the number of nonzero predictors retained in the model.

(B) Cross-validation plot for selection of the optimal penalty parameter λ in the LASSO model. Red dots indicate the partial likelihood deviance at different λ values, gray error bars indicate the standard errors, and the dotted vertical line marks the optimal λ.

(C) Results of candidate predictor selection using the Boruta algorithm. Green, red, and blue box plots denote confirmed, rejected, and tentative predictors, respectively.

(D) Venn diagram showing the overlap between predictors selected by LASSO and Boruta. LASSO identified 19 predictors and Boruta identified 12 predictors, with 9 predictors shared by both methods.

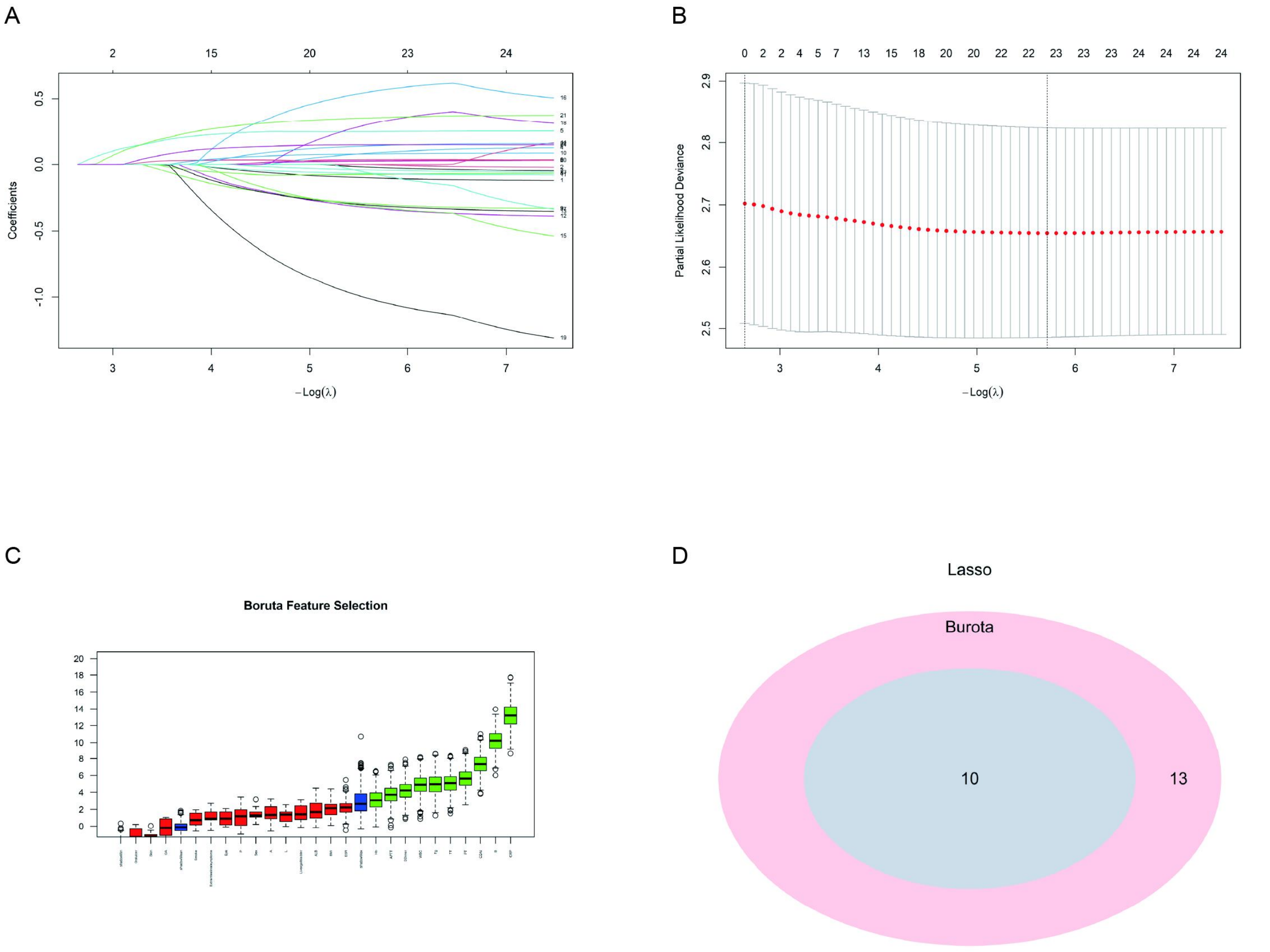

Supplementary Figure 7. Time-dependent receiver operating characteristic (ROC) curves of different models for predicting 1-year surgery risk in five imputed dataset, based on out-of-fold individual risk predictions. The corresponding areas under the curve (AUCs) are presented for each model. Panels A–E represent imputed datasets 1–5, respectively.

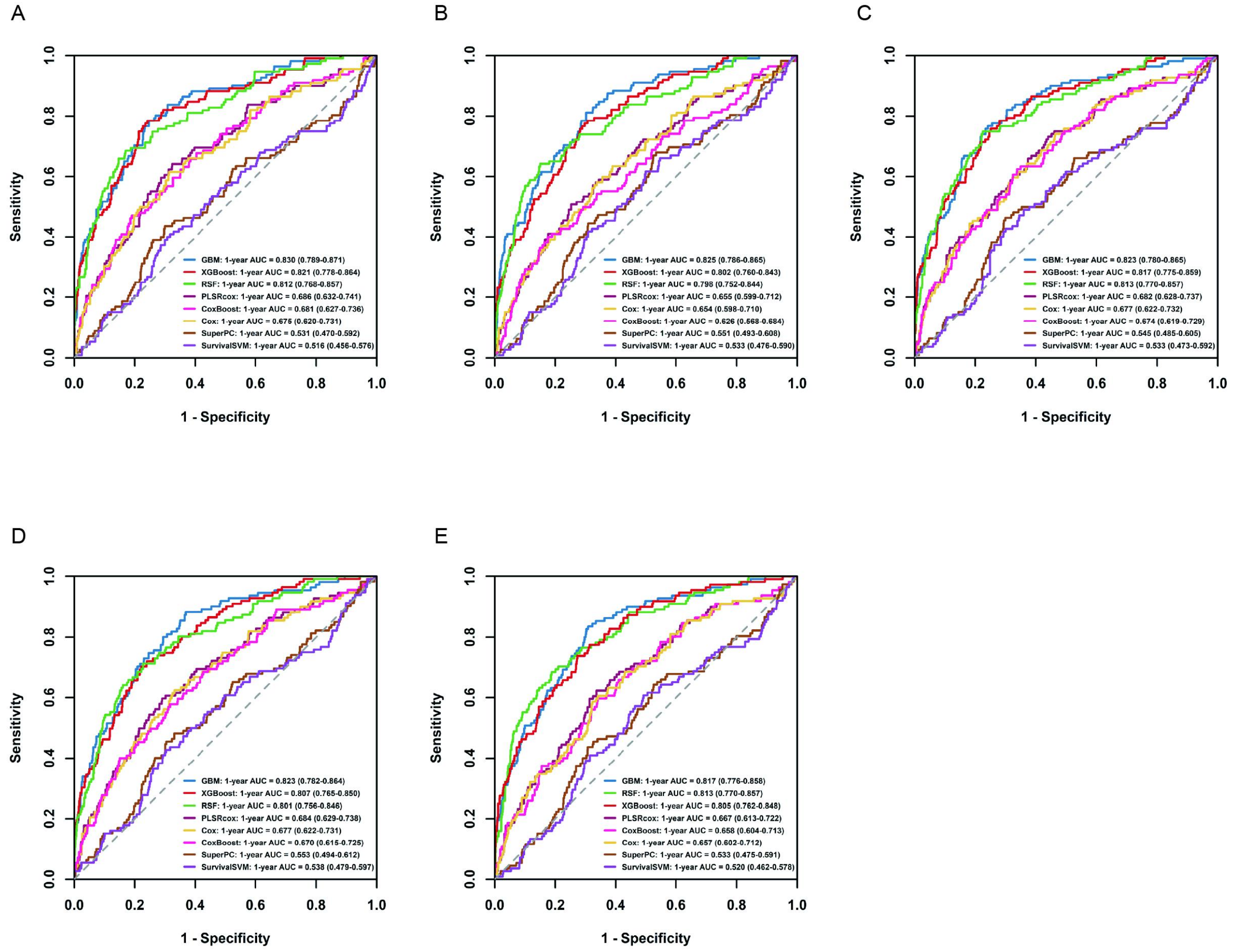

Supplementary Figure 8. Time-dependent receiver operating characteristic (ROC) curves of different models for predicting 3-year surgery risk in five imputed dataset, based on out-of-fold individual risk predictions. The corresponding areas under the curve (AUCs) are presented for each model. Panels A–E represent imputed datasets 1–5, respectively.

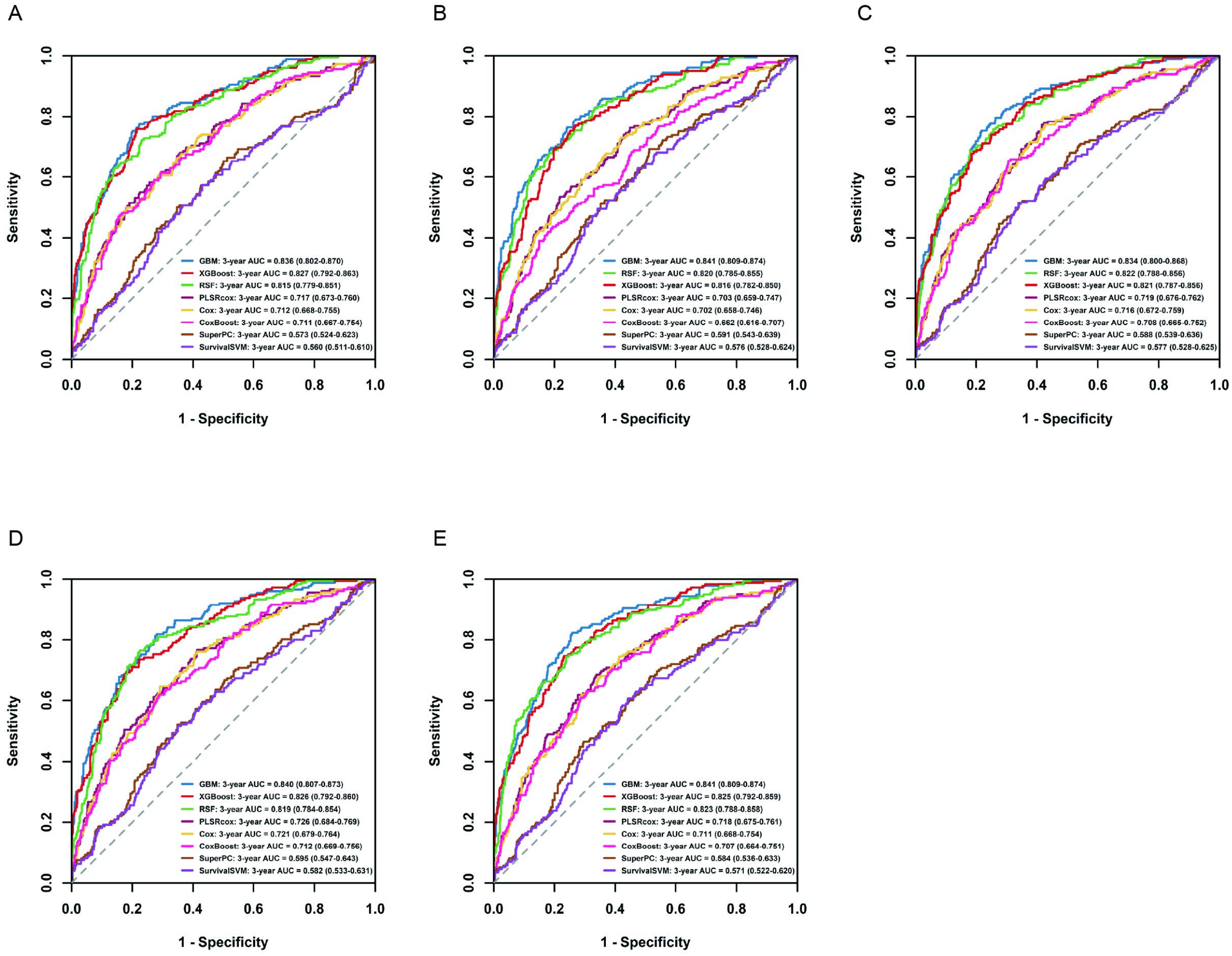

Supplementary Figure 9. Time-dependent receiver operating characteristic (ROC) curves of different models for predicting 5-year surgery risk in five imputed dataset, based on out-of-fold individual risk predictions. The corresponding areas under the curve (AUCs) are presented for each model. Panels A–E represent imputed datasets 1–5, respectively.

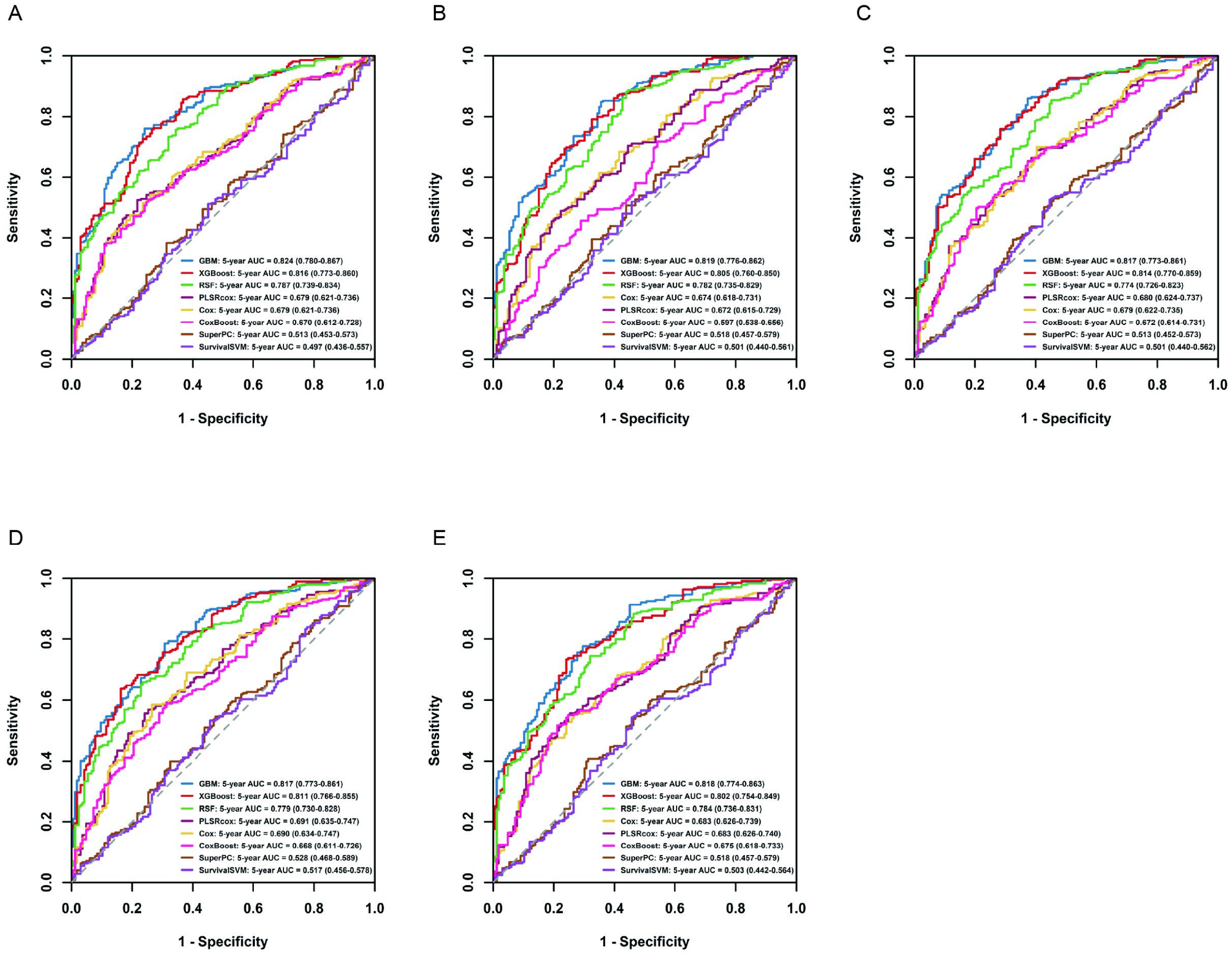

Supplementary Figure 10. Calibration curves of the models of different models for predicting 1-year surgery risk in five imputed dataset, based on out-of-fold individual risk predictions. The corresponding Brier scores are presented for each model. Panels A–E represent imputed datasets 1–5, respectively.

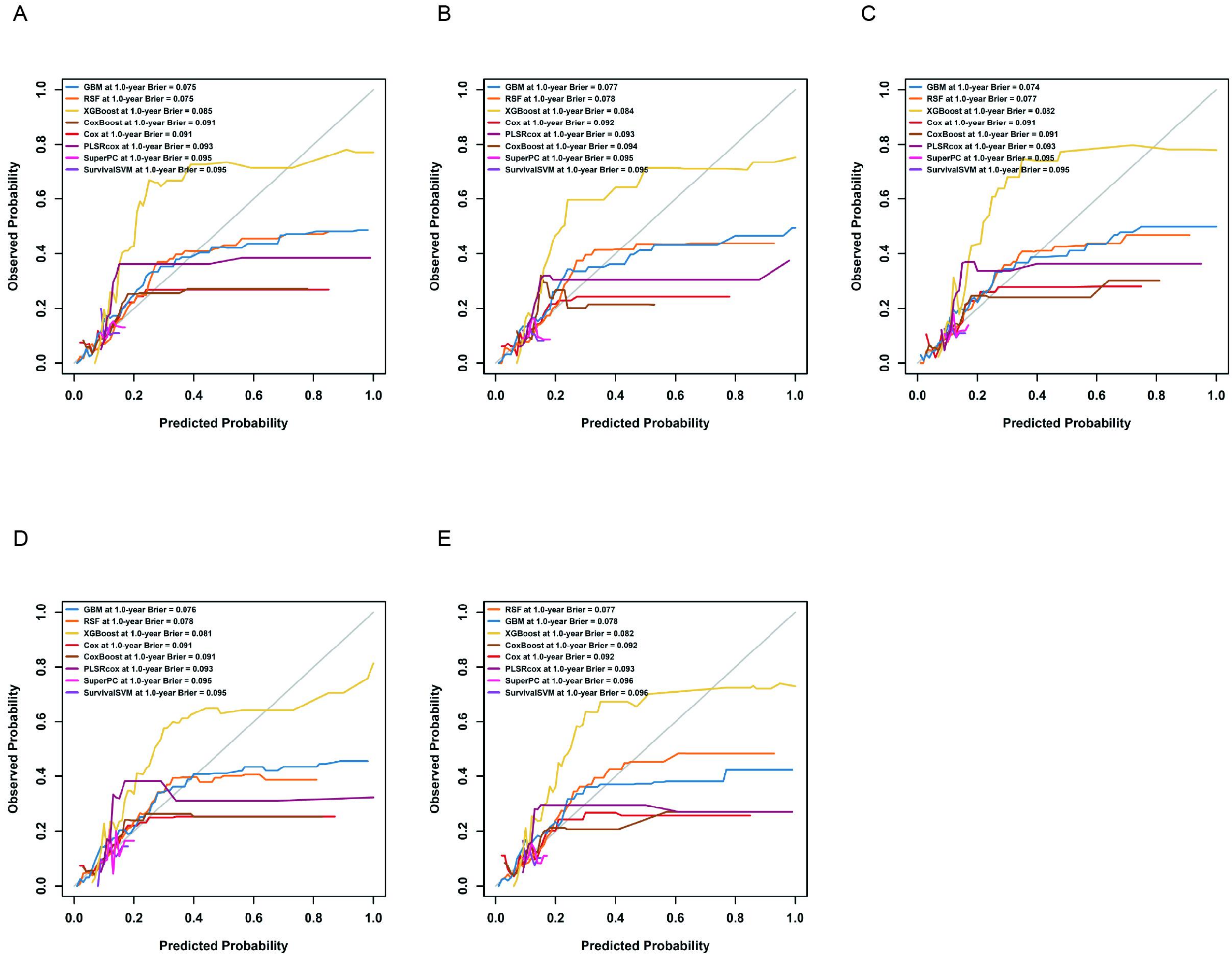

Supplementary Figure 11. Calibration curves of the models of different models for predicting 3-year surgery risk in five imputed dataset, based on out-of-fold individual risk predictions. The corresponding Brier scores are presented for each model. Panels A–E represent imputed datasets 1–5, respectively.

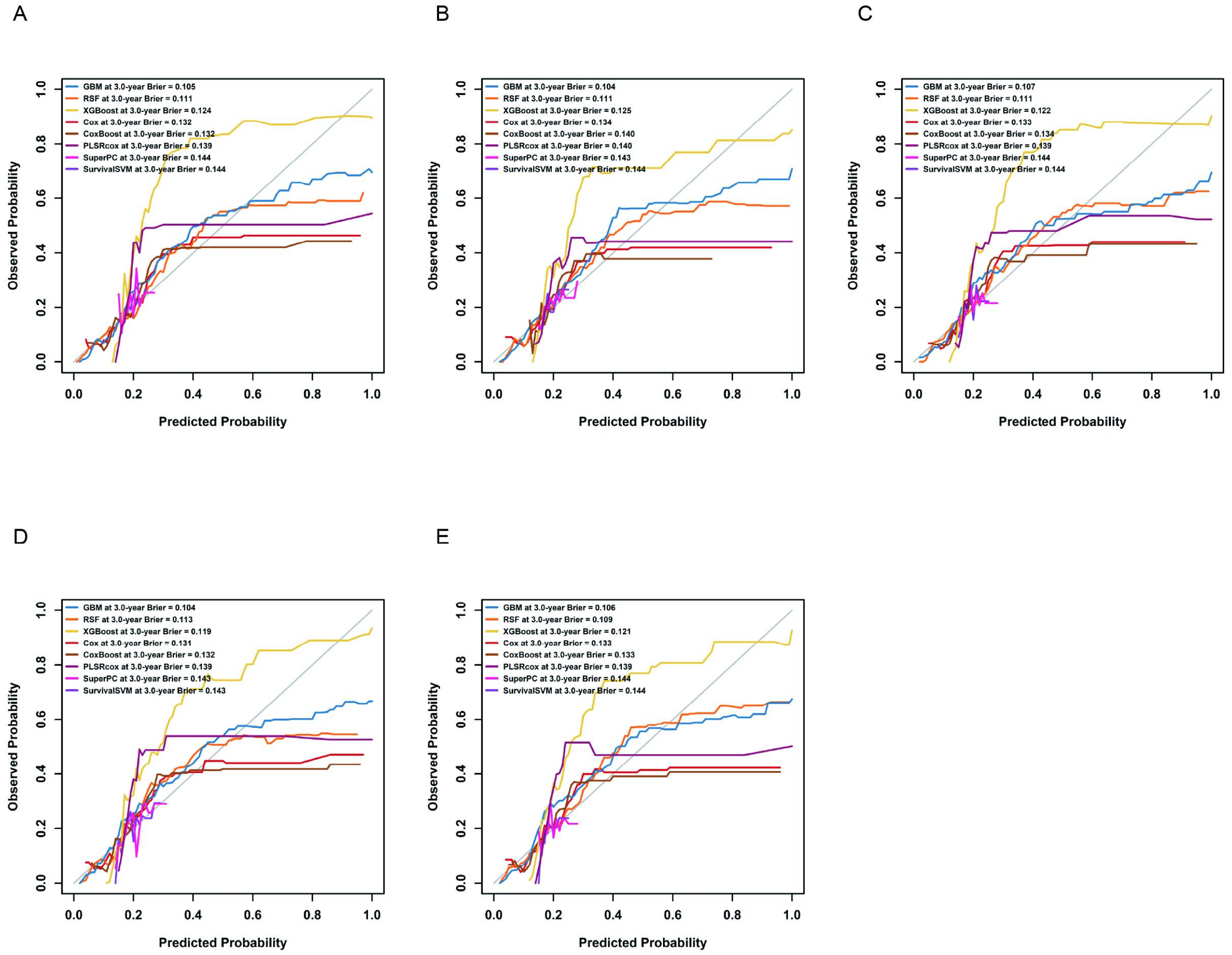

Supplementary Figure 12. Calibration curves of the models of different models for predicting 5-year surgery risk in five imputed dataset, based on out-of-fold individual risk predictions. The corresponding Brier scores are presented for each model. Panels A–E represent imputed datasets 1–5, respectively.

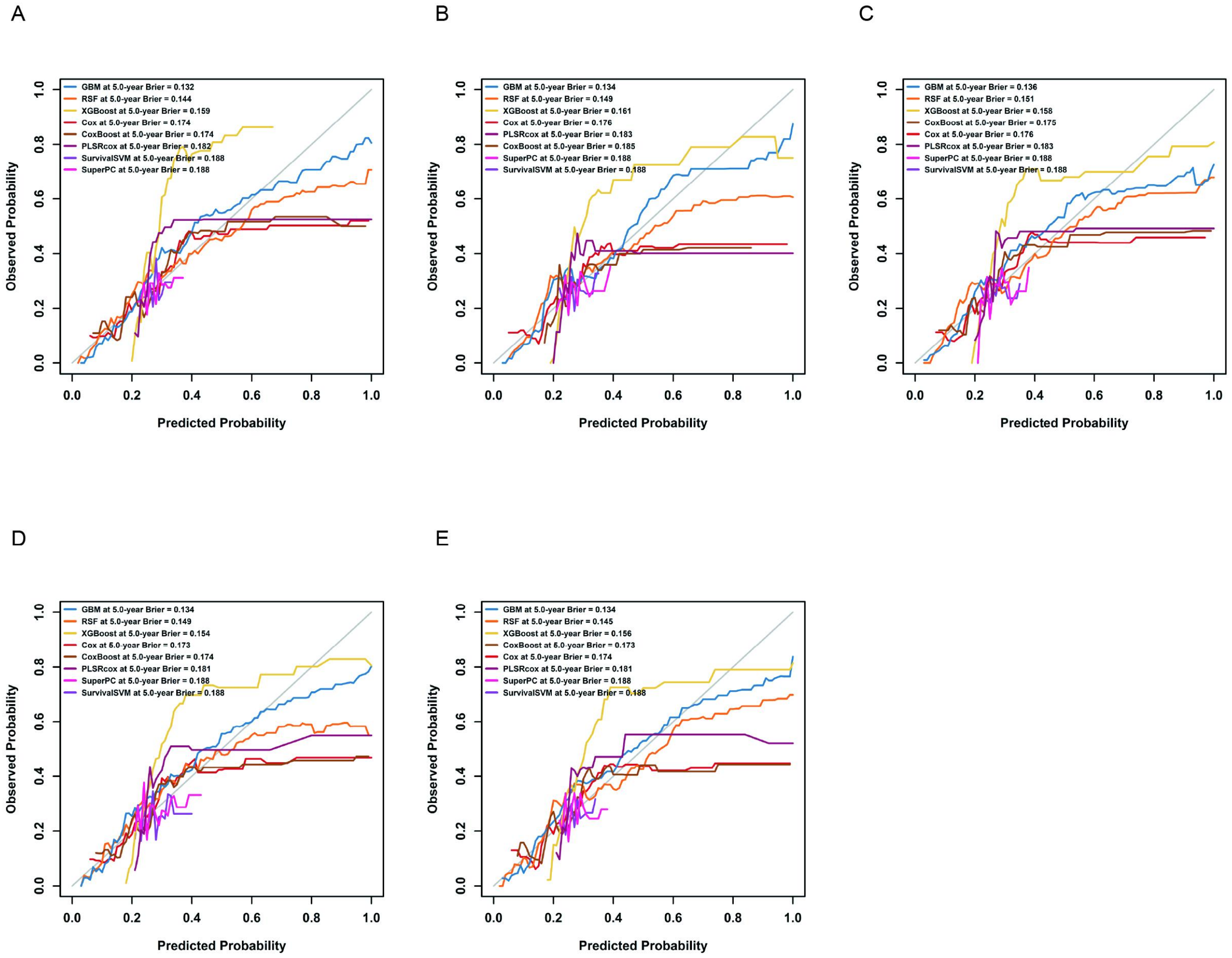

Supplementary Figure 13. Decision curve analysis (DCA) curves of the models for predicting 1-year surgery risk in five imputed dataset, based on out-of-fold individual risk predictions, showing the net clinical benefit of each model across a range of threshold probabilities. Panels A–E represent imputed datasets 1–5, respectively.

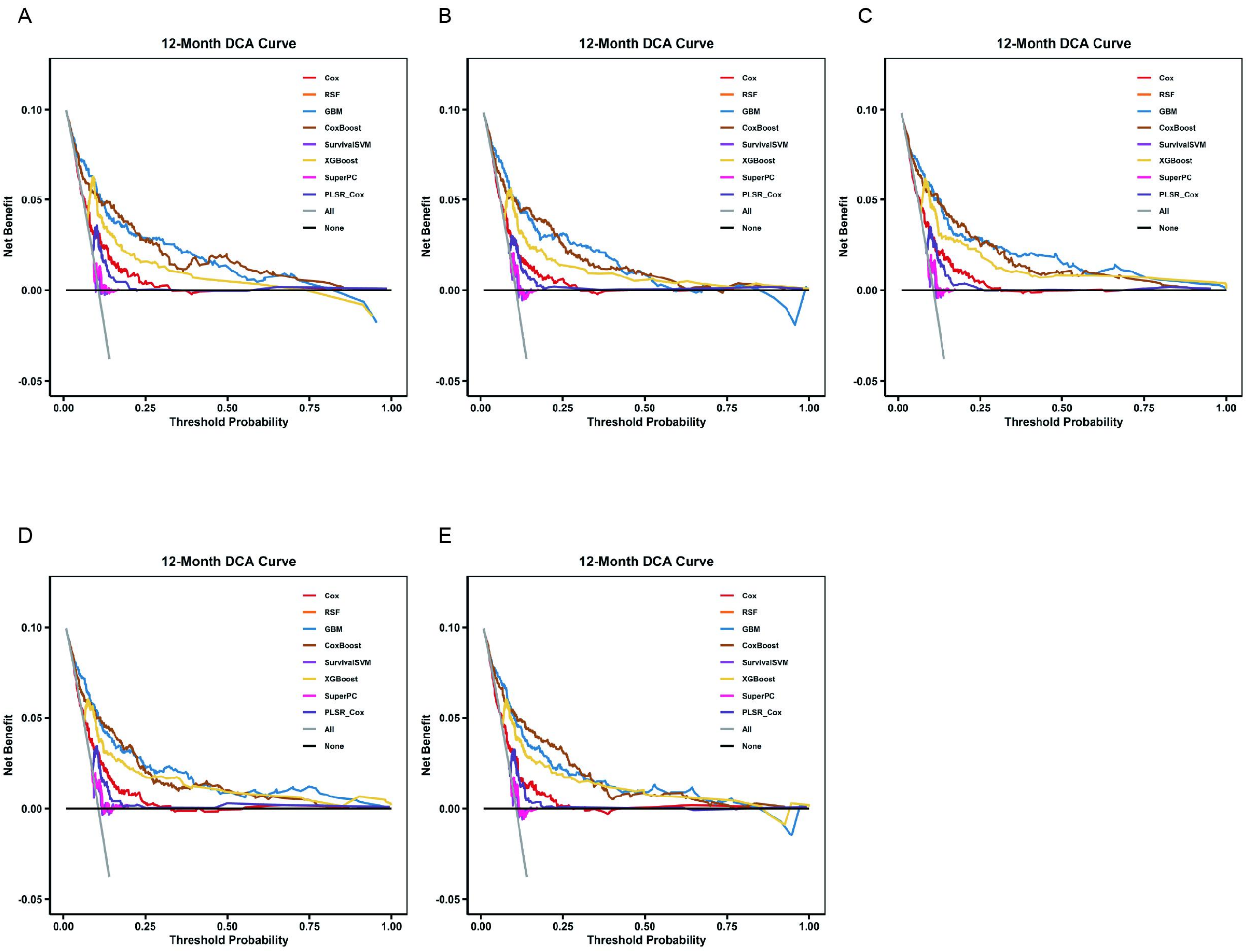

Supplementary Figure 14. Decision curve analysis (DCA) curves of the models for predicting 3-year surgery risk in five imputed dataset, based on out-of-fold individual risk predictions, showing the net clinical benefit of each model across a range of threshold probabilities. Panels A–E represent imputed datasets 1–5, respectively.

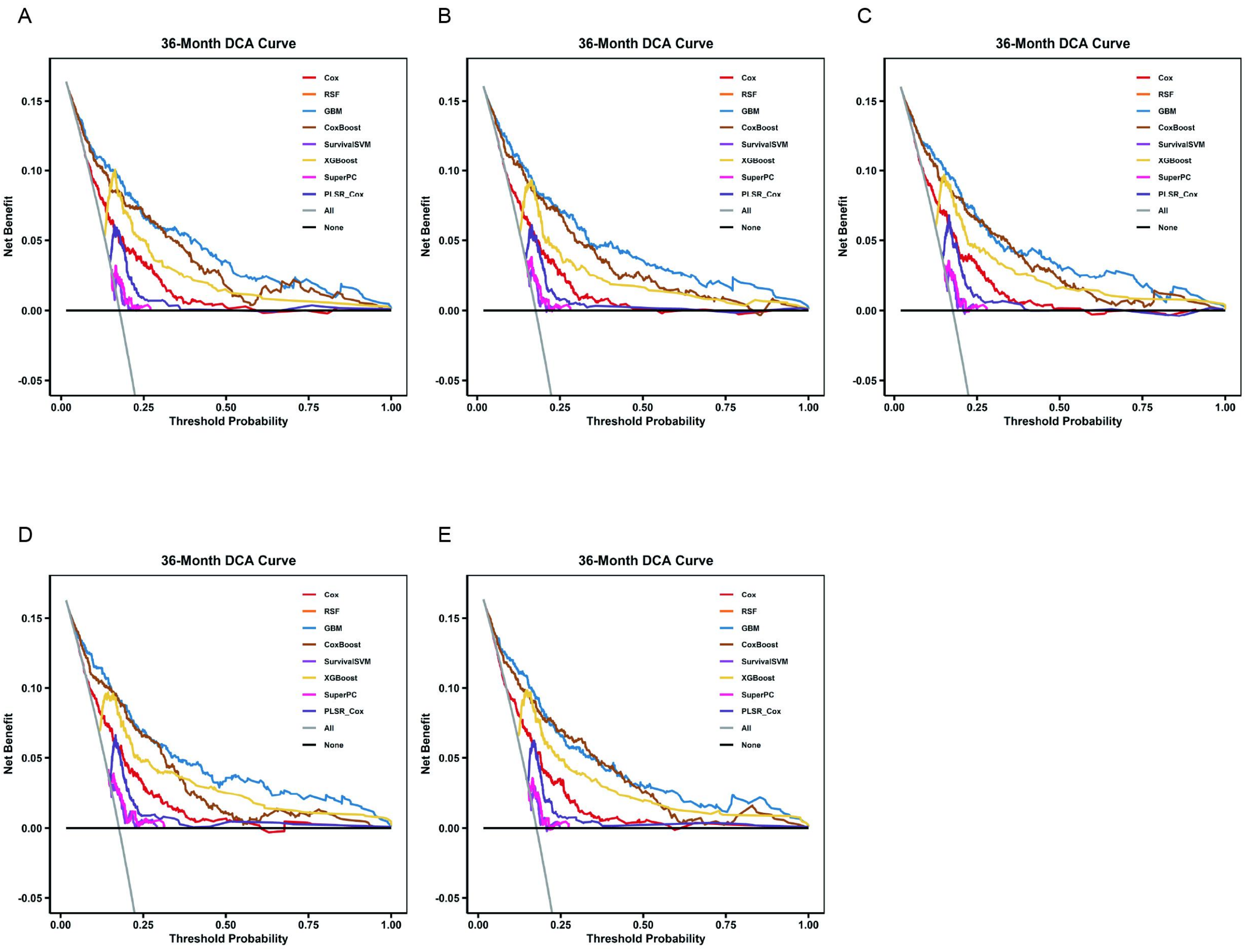

Supplementary Figure 15. Decision curve analysis (DCA) curves of the models for predicting 5-year surgery risk in five imputed dataset, based on out-of-fold individual risk predictions, showing the net clinical benefit of each model across a range of threshold probabilities. Panels A–E represent imputed datasets 1–5, respectively.

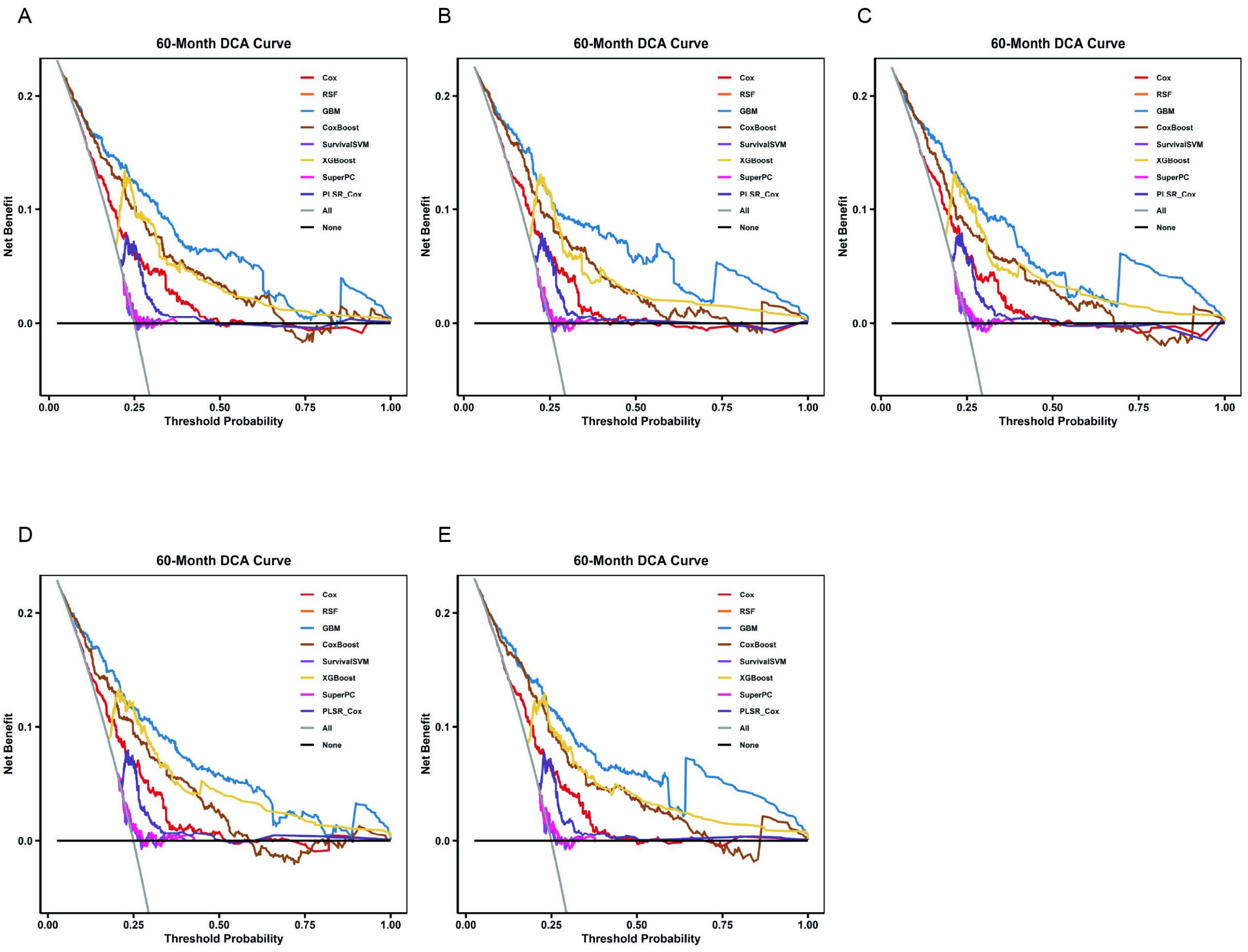

Supplementary Figure 16. Comparison of cumulative surgery-free rates between patients with and without biologic therapy across gradient boosting machine (GBM)-defined risk strata after inverse probability of treatment weighting (IPTW) in imputed datasets 2–5. Panels A–D correspond to low-risk patients in imputed datasets 2–5, panels E–H correspond to intermediate-risk patients in imputed datasets 2–5, and panels I–L correspond to high-risk patients in imputed datasets 2–5.

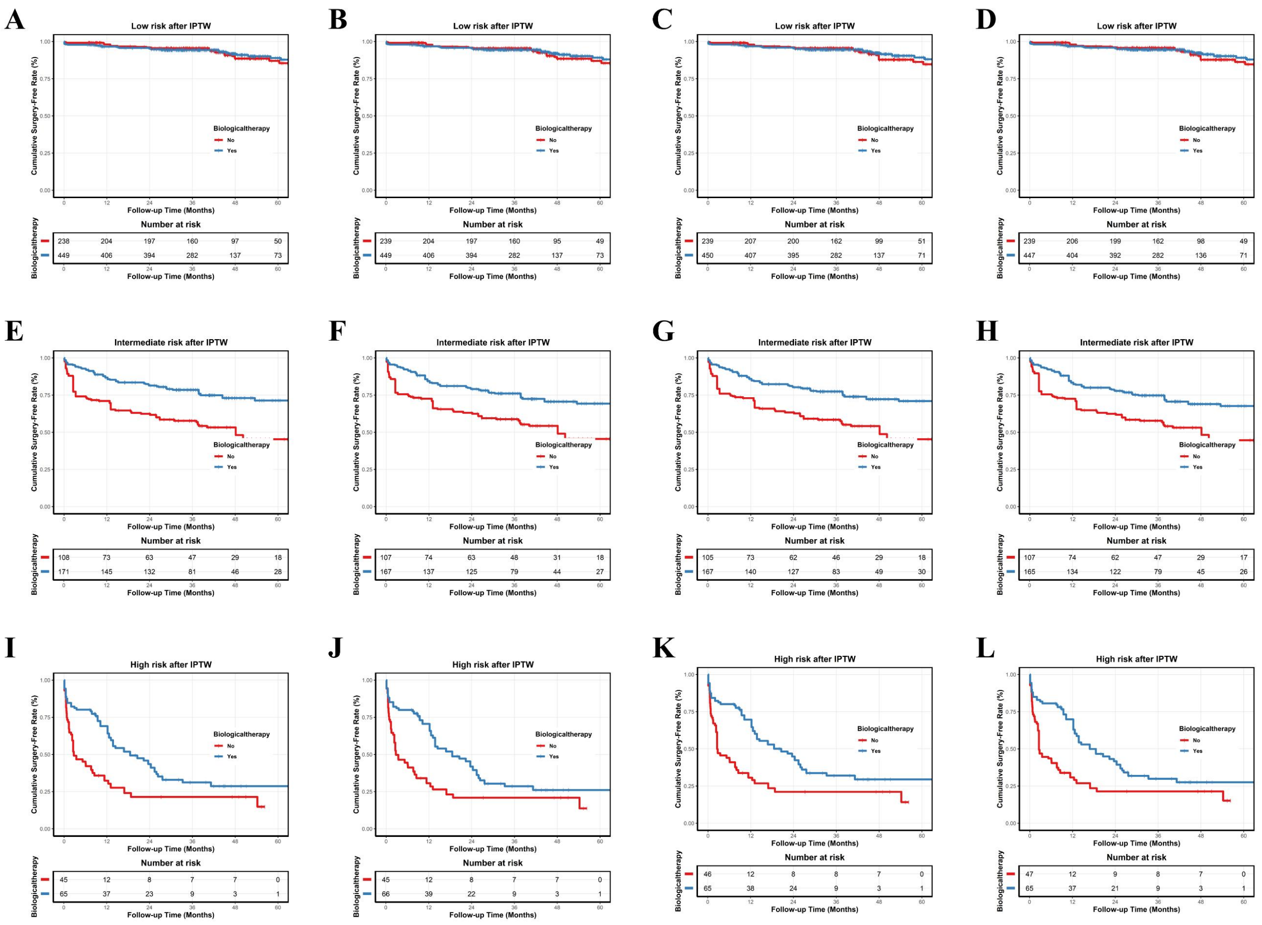

Supplementary Figure 17.

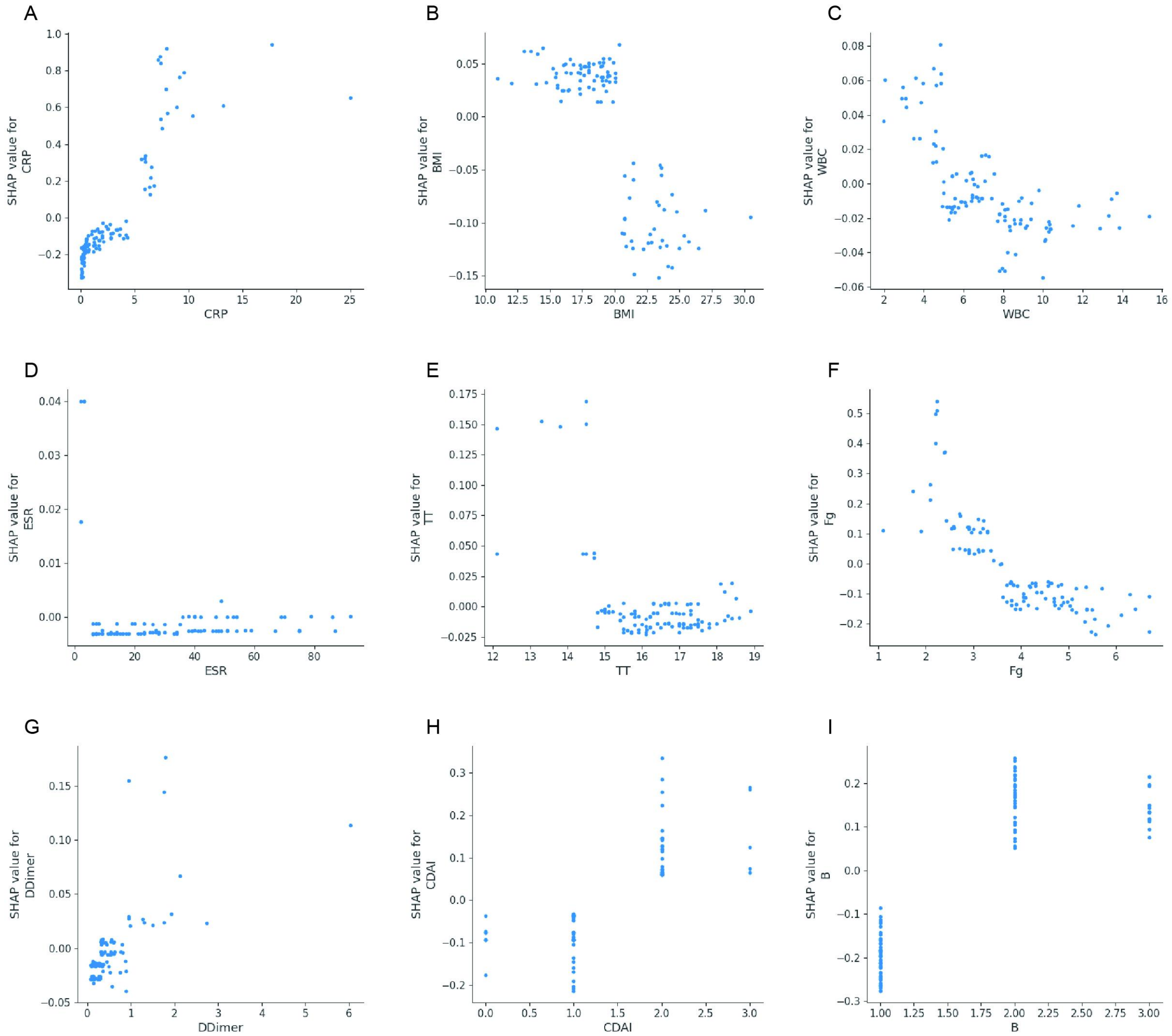

Supplement: Supplementary file 2 [file DataSheet2.pdf]
