# Supplementary material for: Integrating inflammatory and coagulation biomarkers for surgical risk stratification and treatment benefit assessment in Crohn’s disease
Source: Front Immunol. 2026 May 7;17:1657279. doi: 10.3389/fimmu.2026.1657279 (PMC13189891; doi:10.3389/fimmu.2026.1657279)
Supplement: Supplementary file 3 [file DataSheet3.pdf]

Supplementary Table 1. Concordance indices (C-indices) of different models based on out-of-fold individual risk predictions in each of the five imputed datasets

	Imputed dataset 1	Imputed dataset 2	Imputed dataset 3	Imputed dataset 4	Imputed dataset 5	Mean
Cox	0.678(0.640-0.715)	0.667(0.630-0.704)	0.679(0.642-0.716)	0.683(0.646-0.720)	0.674(0.637-0.711)	0.676(0.639-0.713)
RSF	0.783(0.752-0.814)	0.779(0.748-0.810)	0.784(0.755-0.813)	0.781(0.750-0.812)	0.789(0.758-0.820)	0.783(0.753-0.814)
GBM	0.804(0.777-0.831)	0.804(0.777-0.831)	0.801(0.772-0.830)	0.805(0.778-0.832)	0.804(0.777-0.831)	0.804(0.775-0.831)
CoxBoost	0.679(0.642-0.716)	0.628(0.589-0.667)	0.675(0.638-0.712)	0.678(0.641-0.715)	0.671(0.634-0.708)	0.666(0.629-0.704)
SurvivalSVM	0.535(0.492-0.578)	0.547(0.506-0.588)	0.546(0.505-0.587)	0.554(0.513-0.595)	0.542(0.501-0.583)	0.545(0.503-0.586)
XGBoost	0.798(0.789-0.827)	0.786(0.757-0.815)	0.794(0.767-0.823)	0.795(0.767-0.824)	0.794(0.767-0.823)	0.793(0.769-0.822)
SuperPC	0.547(0.504-0.590)	0.56(0.519-0.601)	0.556(0.515-0.597)	0.566(0.525-0.607)	0.553(0.519-0.594)	0.556(0.516-0.598)
PLSR-Cox	0.682(0.645-0.719)	0.667(0.630-0.704)	0.682(0.645-0.719)	0.687(0.650-0.724)	0.679(0.642-0.716)	0.679(0.642-0.716)

Supplementary Table 2. 1-year areas under the curve (AUCs) of each model based on out-of-fold individual risk predictions in each of the five imputed datasets.

	Imputed dataset 1	Imputed dataset 2	Imputed dataset 3	Imputed dataset 4	Imputed dataset 5	Mean
Cox	0.675(0.620-0.731)	0.654(0.598-0.710)	0.677(0.622-0.732)	0.677(0.622-0.731)	0.657(0.602-0.712)	0.668(0.613-0.723)
RSF	0.812 (0.768-0.857)	0.798(0.752-0.844)	0.813(0.770-0.857)	0.801(0.756-0.846)	0.813(0.770-0.857)	0.807(0.763-0.852)
GBM	0.830(0.789-0.871)	0.825(0.786-0.865)	0.823(0.780-0.865)	0.823(0.782-0.864)	0.817(0.776-0.858)	0.824(0.783-0.865)
CoxBoost	0.681(0.627-0.736)	0.626(0.568-0.684)	0.674(0.619-0.729)	0.670(0.615-0.725)	0.658(0.604-0.713)	0.662(0.607-0.717)
SurvivalSVM	0.516(0.456-0.576)	0.533(0.476-0.590)	0.533(0.473-0.592)	0.538(0.479-0.597)	0.520(0.462-0.578)	0.528(0.469-0.587)
XGBoost	0.821(0.778-0.864)	0.802(0.760-0.843)	0.817(0.775-0.859)	0.807(0.765-0.850)	0.805(0.762-0.848)	0.810(0.768-0.853)
SuperPC	0.531(0.470-0.592)	0.551(0.493-0.608)	0.545(0.485-0.605)	0.553(0.494-0.612)	0.533(0.475-0.591)	0.543(0.483-0.602)
PLSR-Cox	0.686(0.632-0.741)	0.655(0.599-0.712)	0.682(0.628-0.737)	0.684(0.629-0.738)	0.667(0.613-0.722)	0.675(0.620-0.730)

Supplementary Table 3. 3-year areas under the curve (AUCs) of each model based on out-of-fold individual risk predictions in each of the five imputed datasets.

	Imputed dataset 1	Imputed dataset 2	Imputed dataset 3	Imputed dataset 4	Imputed dataset 5	Mean
Cox	0.712(0.668-0.755)	0.702(0.658-0.746)	0.716(0.672-0.759)	0.721(0.679-0.764)	0.711(0.668-0.754)	0.712(0.669-0.756)
RSF	0.815(0.779-0.851)	0.820(0.785-0.855)	0.822(0.788-0.856)	0.819(0.784-0.854)	0.823(0.788-0.858)	0.820(0.785-0.855)
GBM	0.836(0.802-0.870)	0.841(0.809-0.874)	0.834(0.800-0.868)	0.840(0.807-0.873)	0.841(0.809-0.874)	0.838(0.805-0.872)
CoxBoost	0.711(0.667-0.754)	0.662(0.616-0.707)	0.708(0.665-0.752)	0.712(0.669-0.756)	0.707(0.664-0.751)	0.700(0.656-0.744)
SurvivalSVM	0.560(0.511-0.610)	0.576(0.528-0.624)	0.577(0.528-0.625)	0.582(0.533-0.631)	0.571(0.522-0.620)	0.573(0.524-0.622)
XGBoost	0.827(0.792-0.863)	0.816(0.782-0.850)	0.821(0.787-0.856)	0.826(0.792-0.860)	0.825(0.792-0.859)	0.823(0.789-0.858)
SuperPC	0.573(0.524-0.623)	0.591(0.543-0.639)	0.588(0.539-0.636)	0.595(0.547-0.643)	0.584(0.536-0.633)	0.586(0.538-0.635)
PLSR-Cox	0.717(0.673-0.760)	0.703(0.659-0.747)	0.719(0.676-0.762)	0.726(0.684-0.769)	0.718(0.675-0.761)	0.717(0.673-0.760)

Supplementary Table 4. 5-year areas under the curve (AUCs) of each model based on out-of-fold individual risk predictions in each of the five imputed datasets.

	Imputed dataset 1	Imputed dataset 2	Imputed dataset 3	Imputed dataset 4	Imputed dataset 5	Mean
Cox	0.679(0.621-0.736)	0.674(0.618-0.731)	0.679(0.622-0.735)	0.690(0.634-0.747)	0.683(0.626-0.739)	0.681(0.624-0.738)
RSF	0.787(0.739-0.834)	0.782(0.735-0.829)	0.774(0.726-0.823)	0.779(0.730-0.828)	0.784(0.736-0.831)	0.781(0.733-0.829)
GBM	0.824(0.780-0.867)	0.819(0.776-0.862)	0.817(0.773-0.861)	0.817(0.773-0.861)	0.818(0.774-0.863)	0.819(0.775-0.863)
CoxBoost	0.670(0.612-0.728)	0.597(0.538-0.656)	0.672(0.614-0.731)	0.690(0.634-0.747)	0.675(0.618-0.733)	0.661(0.603-0.719)
SurvivalSVM	0.497(0.436-0.557)	0.501(0.440-0.561)	0.501(0.440-0.562)	0.517(0.456-0.578)	0.518(0.457-0.579)	0.507(0.446-0.567)
XGBoost	0.816(0.773-0.860)	0.805(0.760-0.850)	0.814(0.770-0.859)	0.811(0.766-0.855)	0.802(0.754-0.849)	0.810(0.765-0.855)
SuperPC	0.513(0.453-0.573)	0.518(0.457-0.579)	0.513(0.452-0.573)	0.528(0.468-0.589)	0.503(0.442-0.564)	0.515(0.454-0.576)
PLSR-Cox	0.679(0.621-0.736)	0.672(0.615-0.729)	0.680(0.624-0.737)	0.691(0.635-0.747)	0.683(0.626-0.740)	0.681(0.624-0.738)

Supplementary Table 5. 1-year brier score of each model based on out-of-fold individual risk predictions in each of the five imputed datasets.

	Imputed dataset 1	Imputed dataset 2	Imputed dataset 3	Imputed dataset 4	Imputed dataset 5	Mean
Cox	0.091	0.092	0.091	0.091	0.092	0.091
RSF	0.075	0.078	0.077	0.078	0.077	0.077
GBM	0.075	0.077	0.074	0.076	0.078	0.076
CoxBoost	0.091	0.094	0.091	0.091	0.092	0.092
SurvivalSVM	0.095	0.095	0.095	0.095	0.096	0.095
XGBoost	0.085	0.084	0.082	0.081	0.082	0.083
SuperPC	0.095	0.095	0.095	0.095	0.096	0.095
PLSR-Cox	0.093	0.093	0.093	0.093	0.093	0.093

Supplementary Table 6. 3-year brier score of each model based on out-of-fold individual risk predictions in each of the five imputed datasets.

	Imputed dataset 1	Imputed dataset 2	Imputed dataset 3	Imputed dataset 4	Imputed dataset 5	Mean
Cox	0.132	0.134	0.133	0.131	0.133	0.133
RSF	0.111	0.111	0.111	0.113	0.109	0.111
GBM	0.105	0.104	0.107	0.104	0.106	0.105
CoxBoost	0.132	0.140	0.134	0.132	0.133	0.134
SurvivalSVM	0.144	0.144	0.144	0.143	0.144	0.144
XGBoost	0.124	0.125	0.122	0.119	0.121	0.122
SuperPC	0.144	0.143	0.144	0.143	0.144	0.144
PLSR-Cox	0.139	0.140	0.139	0.139	0.139	0.139

Supplementary Table 7. 5-year Brier score of each model based on out-of-fold individual risk predictions in each of the five imputed datasets.

	Imputed dataset 1	Imputed dataset 2	Imputed dataset 3	Imputed dataset 4	Imputed dataset 5	Mean
Cox	0.174	0.176	0.176	0.173	0.174	0.175
RSF	0.145	0.149	0.151	0.149	0.145	0.148
GBM	0.134	0.134	0.136	0.134	0.134	0.134
CoxBoost	0.173	0.185	0.175	0.174	0.173	0.176
SurvivalSVM	0.188	0.188	0.188	0.188	0.188	0.188
XGBoost	0.156	0.161	0.158	0.154	0.156	0.157
SuperPC	0.188	0.188	0.188	0.188	0.188	0.188
PLSR-Cox	0.181	0.183	0.183	0.181	0.181	0.182

Supplementary Table 8. Average performance of different models based on individual risk predictions across the five imputed datasets without 5-fold cross-validation.

	C-index	1-year AUC	3-year AUC	5-year AUC	1-year Brier	3-year Brier	5-year Brier	IBS	Slope
Cox	0.699 (0.664-0.734)	0.692 (0.638-0.746)	0.737(0.694-0.780)	0.706 (0.650-0.762)	0.086 (0.073-0.100)	0.126 (0.113-0.140)	0.166 (0.148-0.184)	0.126	1.021
RSF	0.818 (0.790-0.846)	0.839 (0.799-0.879)	0.850 (0.818-0.882)	0.809 (0.764-0.854)	0.071 (0.060-0.082)	0.102 (0.090-0.114)	0.137 (0.118-0.156)	0.103	0.985
GBM	0.843 (0.816-0.870)	0.858 (0.820-0.896)	0.871 (0.840-0.902)	0.851 (0.811-0.891)	0.069 (0.058-0.081)	0.096 (0.084-0.108)	0.123 (0.106-0.140)	0.097	0.993
CoxBoost	0.691 (0.654-0.728)	0.684 (0.628-0.740)	0.726 (0.683-0.769)	0.668 (0.611-0.725)	0.088 (0.074-0.102)	0.128 (0.114-0.142)	0.171 (0.153-0.189)	0.129	1.014
SurvivalSVM	0.567 (0.527-0.607)	0.549 (0.490-0.608)	0.596 (0.548-0.644)	0.523 (0.463-0.583)	0.092 (0.079-0.106)	0.139 (0.125-0.154)	0.182 (0.166-0.199)	0.138	0.963
XGBoost	0.833 (0.806-0.860)	0.850 (0.812-0.888)	0.862 (0.830-0.894)	0.848 (0.807-0.889)	0.076 (0.063-0.089)	0.111 (0.098-0.124)	0.146 (0.131-0.162)	0.111	0.969
SuperPC	0.574 (0.539-0.609)	0.562 (0.503-0.621)	0.607 (0.560-0.654)	0.538 (0.477-0.599)	0.092 (0.079-0.106)	0.139 (0.124-0.154)	0.183 (0.166-0.199)	0.138	0.961
PLSR-Cox	0.702 (0.667-0.737)	0.696 (0.642-0.750)	0.742 (0.700-0.784)	0.706 (0.651-0.761)	0.089 (0.075-0.103)	0.132 (0.118-0.146)	0.175 (0.158-0.192)	0.132	0.991

Supplementary Table 9. Baseline characteristics of patients with and without biologic therapy across gradient boosting machine (GBM)-defined low-, intermediate-, and high-risk strata before inverse probability of treatment weighting in Imputed dataset 1

	Low-risk			Intermediate-risk			High-risk		
	No-Biologics	Biologics	*P*	No-Biologics	Biologics	*P*	No-Biologics	Biologics	*P*
	n =241	n =447		n =105	n =154		n =47	n =66	
**Demographic data**									
Gender (n, %)			0.063			0.134			1
Male	191 (79.3)	324 (72.5)		82 (78.1)	106 (68.8)		35 (74.5)	48 (72.7)	
Female	50 (20.7)	123 (27.5)		23 (21.9)	48 (31.2)		12 (25.5)	18 (27.3)	
Smoking (n, %)			<0.001			0.04			0.423
No	180 (74.7)	384 (85.9)		72 (68.6)	124 (80.5)		30 (63.8)	48 (72.7)	
Yes	61 (25.3)	63 (14.1)		33 (31.4)	30 (19.5)		17 (36.2)	18 (27.3)	
BMI (kg/m^2)	19.26 (17.36, 21.88)	19.05 (17.30, 21.22)	0.116	17.71 (16.16, 20.07)	17.30 (16.14, 19.38)	0.138	17.31 (16.32, 18.95)	17.28 (15.96, 19.92)	0.445
**Laboratory data**									
WBC (10^9/L)	6.22 (5.14, 8.21)	6.51 (5.22, 8.09)	0.382	6.37 (4.54, 9.70)	6.33 (4.77, 8.31)	0.66	6.44 (4.68, 7.46)	6.70 (4.55, 9.74)	0.225
Hb (g/dL)	12.30 (10.80, 13.80)	12.20 (10.75, 13.65)	0.523	11.90 (9.90, 13.30)	11.90 (9.95, 13.60)	0.678	11.40 (9.40, 12.80)	10.00 (8.60, 11.57)	0.035
ESR (mm/h)	15.00 (7.00, 34.00)	19.00 (10.50, 40.00)	0.001	23.00 (6.00, 40.00)	24.00 (9.00, 46.75)	0.245	43.00 (17.00, 52.50)	37.00 (19.25, 62.00)	0.566
CRP (mg/dL)	1.15 (0.35, 2.49)	1.16 (0.40, 2.45)	0.827	2.85 (0.47, 6.44)	1.77 (0.42, 6.56)	0.625	6.59 (1.85, 9.13)	7.34 (1.98, 9.97)	0.413
TT (s)	16.50 (15.90, 17.20)	16.60 (15.80, 17.20)	0.864	16.60 (15.90, 17.10)	16.80 (16.02, 17.20)	0.252	16.90 (16.16, 17.65)	16.30 (15.20, 17.70)	0.046
APTT (s)	29.60 (27.90, 34.10)	29.60 (27.70, 32.45)	0.469	30.50 (28.00, 36.10)	29.90 (27.68, 32.85)	0.276	29.80 (28.70, 39.10)	30.20 (27.95, 35.17)	0.606
PT (s)	11.60 (11.00, 12.40)	11.70 (11.00, 12.70)	0.199	12.30 (11.30, 13.30)	12.15 (11.40, 13.28)	0.924	12.70 (11.95, 13.70)	12.60 (11.70, 13.85)	0.813
Fg (g/L)	3.74 (3.10, 4.31)	3.80 (3.05, 4.58)	0.303	3.34 (2.50, 4.89)	3.20 (2.39, 4.80)	0.646	3.93 (2.38, 4.69)	3.40 (2.76, 4.81)	0.843
DD (mg/L)	0.32 (0.20, 0.56)	0.31 (0.18, 0.56)	0.209	0.34 (0.17, 0.80)	0.34 (0.21, 0.80)	0.894	0.48 (0.26, 1.03)	0.85 (0.35, 1.61)	0.105
ALB (g/dL)	3.76 (3.36, 4.17)	3.77 (3.31, 4.15)	0.853	3.67 (3.19, 4.10)	3.74 (3.21, 4.20)	0.423	3.49 (3.02, 3.91)	3.26 (2.81, 3.71)	0.085
**Crohn's disease**									
Perianal abscess			0.004			0.001			0.311
No	168 (69.7)	261 (58.4)		86 (81.9)	95 (61.7)		35 (74.5)	42 (63.6)	
Yes	73 (30.3)	186 (41.6)		19 (18.1)	59 (38.3)		12 (25.5)	24 (36.4)	
Montreal A			<0.001			0.01			0.703
A1	20 (8.3)	83 (18.6)		5 (4.8)	16 (10.4)		4 (8.5)	5 (7.6)	
A2	182 (75.5)	326 (72.9)		74 (70.5)	120 (77.9)		34 (72.3)	52 (78.8)	

A3	39 (16.2)	38 (8.5)		26 (24.8)	18 (11.7)		9 (19.1)	9 (13.6)	
Montreal B			0.008			0.006			0.314
B1	121 (50.2)	278 (62.2)		16 (15.2)	50 (32.5)		3 (6.4)	5 (7.6)	
B2	60 (24.9)	79 (17.7)		57 (54.3)	72 (46.8)		36 (76.6)	42 (63.6)	
B3	60 (24.9)	90 (20.1)		32 (30.5)	32 (20.8)		8 (17.0)	19 (28.8)	
Montreal L			0.042			0.27			0.145
L1	73 (30.3)	95 (21.3)		33 (31.4)	33 (21.4)		18 (38.3)	16 (24.2)	
L2	14 (5.8)	24 (5.4)		8 (7.6)	12 (7.8)		1 (2.1)	8 (12.1)	
L3	149 (61.8)	322 (72.0)		64 (61.0)	108 (70.1)		27 (57.4)	41 (62.1)	
L4	5 (2.1)	6 (1.3)		0 (0.0)	1 (0.6)		1 (2.1)	1 (1.5)	
CDAI			0.104			0.851			0.735
Remission	38 (15.8)	63 (14.1)		12 (11.4)	16 (10.4)		1 (2.1)	3 (4.5)	
Mild active	131 (54.4)	210 (47.0)		40 (38.1)	63 (40.9)		14 (29.8)	24 (36.4)	
Moderate active	63 (26.1)	158 (35.3)		47 (44.8)	63 (40.9)		27 (57.4)	34 (51.5)	
Severe active	9 (3.7)	16 (3.6)		6 (5.7)	12 (7.8)		5 (10.6)	5 (7.6)	
**Extrintestinal**									
Extrintestinal			0.91			0.921			0.887
No	200 (83.0)	368 (82.3)		88 (83.8)	131 (85.1)		37 (78.7)	50 (75.8)	
Yes	41 (17.0)	79 (17.7)		17 (16.2)	23 (14.9)		10 (21.3)	16 (24.2)	
Oral ulcer			0.724			0.12			0.489
No	219 (90.9)	401 (89.7)		101 (96.2)	139 (90.3)		44 (93.6)	58 (87.9)	
Yes	22 (9.1)	46 (10.3)		4 (3.8)	15 (9.7)		3 (6.4)	8 (12.1)	
Liver/gallbladder			1			0.369			0.696
No	239 (99.2)	442 (98.9)		101 (96.2)	152 (98.7)		44 (93.6)	64 (97.0)	
Yes	2 (0.8)	5 (1.1)		4 (3.8)	2 (1.3)		3 (6.4)	2 (3.0)	
Skin			0.947			1			1
No	233 (96.7)	434 (97.1)		103 (98.1)	150 (97.4)		44 (93.6)	62 (93.9)	
Yes	8 (3.3)	13 (2.9)		2 (1.9)	4 (2.6)		3 (6.4)	4 (6.1)	
Eye			0.229			1			1
No	237 (98.3)	445 (99.6)		103 (98.1)	152 (98.7)		46 (97.9)	65 (98.5)	
Yes	4 (1.7)	2 (0.4)		2 (1.9)	2 (1.3)		1 (2.1)	1 (1.5)	
Osteoarthritis			1			0.195			0.591
No	232 (96.3)	430 (96.2)		100 (95.2)	152 (98.7)		46 (97.9)	62 (93.9)	
Yes	9 (3.7)	17 (3.8)		5 (4.8)	2 (1.3)		1 (2.1)	4 (6.1)	

Supplementary Table 10. Baseline characteristics of patients with and without biologic therapy across gradient boosting machine (GBM)-defined low-, intermediate-, and high-risk strata after inverse probability of treatment weighting in Imputed dataset 1

	Low-risk			Intermediate-risk			High-risk		
	No-Biologics	Biologics	*P*	No-Biologics	Biologics	*P*	No-Biologics	Biologics	*P*
	n =241.7	n =451.7		n =102.9	n =173.6		n =45.2	n =63	
**Demographic data**									
Gender (n, %)			0.982			0.977			0.567
Male	183.8 (76.0)	343.1 (75.9)		79.6 (77.3)	133.9 (77.2)		36.1 (79.7)	47.2 (74.9)	
Female	57.9 (24.0)	108.7 (24.1)		23.3 (22.7)	39.7 (22.8)		9.2 (20.3)	15.8 (25.1)	
Smoking (n, %)			0.983			0.213			1
No	196.3 (81.2)	367.2 (81.3)		76.7 (74.5)	108.6 (62.6)		32.2 (71.1)	44.8 (71.1)	
Yes	45.4 (18.8)	84.6 (18.7)		26.2 (25.5)	65.0 (37.4)		13.1 (28.9)	18.2 (28.9)	
BMI (kg/m^2)	18.73 (17.21, 21.35)	19.10 (17.30, 21.26)	0.467	17.30 (15.87, 19.51)	17.30 (16.40, 19.84)	0.46	17.26 (15.92, 18.75)	17.23 (15.94, 19.70)	0.973
**Laboratory data**									
WBC(10^9/L)	6.46 (5.26, 8.33)	6.43 (5.19, 8.06)	0.462	6.89 (4.53, 9.20)	6.33 (5.04, 8.40)	0.95	6.76 (5.10, 7.48)	6.03 (4.35, 9.45)	0.914
Hb (g/dL)	12.30 (10.36, 13.60)	12.20 (10.80, 13.70)	0.933	12.10 (10.30, 13.30)	11.72 (9.23, 13.60)	0.509	10.81 (8.68, 12.38)	10.38 (8.91, 11.76)	0.604
ESR (mm/h)	16.00 (7.00, 37.34)	18.00 (9.00, 38.00)	0.468	24.33 (7.00, 43.09)	27.00 (9.00, 44.37)	0.445	43.70 (23.13, 51.00)	36.00 (17.51, 58.86)	0.894
CRP (mg/dL)	1.09 (0.41, 2.24)	1.14 (0.35, 2.48)	0.791	2.52 (0.46, 6.46)	1.72 (0.57, 7.27)	0.708	7.18 (5.25, 9.07)	6.99 (1.57, 9.33)	0.723
TT (s)	16.50 (15.90, 17.21)	16.60 (15.80, 17.20)	0.767	16.70 (16.11, 17.10)	16.70 (15.60, 17.20)	0.971	16.70 (16.10, 17.30)	16.70 (15.40, 17.91)	0.702
APTT (s)	29.40 (27.84, 33.53)	29.60 (27.70, 32.70)	0.809	29.20 (27.50, 33.19)	30.20 (27.90, 32.69)	0.397	29.53 (28.32, 35.29)	30.28 (28.80, 35.24)	0.503
PT (s)	11.70 (11.10, 12.54)	11.60 (11.00, 12.70)	0.927	11.73 (11.13, 12.90)	12.15 (11.40, 12.80)	0.161	12.62 (11.67, 13.37)	12.42 (11.52, 13.70)	0.848
Fg (g/L)	3.80 (3.10, 4.41)	3.80 (3.03, 4.54)	0.94	3.34 (2.50, 4.41)	3.50 (2.40, 4.80)	0.562	3.94 (2.74, 4.43)	3.40 (2.48, 4.85)	0.678
DD (mg/L)	0.31 (0.22, 0.56)	0.31 (0.18, 0.55)	0.204	0.28 (0.14, 0.68)	0.31 (0.12, 0.80)	0.915	0.52 (0.27, 0.98)	0.77 (0.24, 1.38)	0.556
ALB (g/dL)	3.74 (3.34, 4.15)	3.72 (3.30, 4.15)	0.897	3.85 (3.22, 4.13)	3.60 (3.24, 4.07)	0.427	3.36 (2.86, 3.83)	3.35 (2.86, 3.78)	0.824
**Crohn's disease**									
Perianal abscess			0.779			0.987			0.735
No	155.3 (64.3)	284.8 (63.0)		75.5 (73.4)	127.6 (73.5)		28.2 (62.3)	41.6 (66.0)	
Yes	86.3 (35.7)	166.9 (37.0)		27.4 (26.6)	46.0 (26.5)		17.1 (37.7)	21.4 (34.0)	
Montreal A			0.973			0.14			0.819
A1	36.2 (15.0)	66.4 (14.7)		17.4 (16.9)	12.8 (7.4)		5.3 (11.7)	5.1 (8.0)	
A2	179.2 (74.2)	338.6 (75.0)		69.1 (67.2)	109.7 (63.2)		34.7 (76.7)	50.3 (79.9)	

A3	26.3 (10.9)	46.7 (10.3)		16.4 (15.9)	51.0 (29.4)		5.2 (11.5)	7.6 (12.1)	
Montreal B			0.974			0.962			0.816
B1	137.3 (56.8)	260.9 (57.7)		24.1 (23.4)	38.1 (21.9)		5.8 (12.8)	5.4 (8.5)	
B2	49.0 (20.3)	90.4 (20.0)		55.5 (53.9)	93.0 (53.6)		29.0 (64.1)	41.2 (65.4)	
B3	55.4 (22.9)	100.5 (22.3)		23.3 (22.7)	42.6 (24.5)		10.5 (23.1)	16.5 (26.1)	
Montreal L			0.991			0.666			0.829
L1	62.8 (26.0)	114.4 (25.3)		24.5 (23.8)	40.3 (23.2)		14.9 (33.0)	19.7 (31.3)	
L2	13.2 (5.5)	23.9 (5.3)		11.2 (10.9)	11.1 (6.4)		1.7 (3.7)	5.3 (8.4)	
L3	162.4 (67.2)	306.2 (67.8)		67.2 (65.3)	121.5 (70.0)		28.0 (61.9)	37.0 (58.8)	
L4	3.2 (1.3)	7.2 (1.6)		0.0 (0.0)	0.6 (0.3)		0.7 (1.5)	0.9 (1.5)	
CDAI			0.894			0.806			0.986
Remission	37.8 (15.7)	64.4 (14.3)		10.0 (9.7)	14.8 (8.5)		1.8 (3.9)	2.9 (4.5)	
Mild active	115.7 (47.9)	230.3 (51.0)		46.4 (45.1)	68.3 (39.4)		13.7 (30.4)	20.5 (32.5)	
Moderate active	80.4 (33.2)	141.3 (31.3)		42.4 (41.2)	81.3 (46.8)		24.9 (55.1)	34.3 (54.5)	
Severe active	7.8 (3.2)	15.7 (3.5)		4.2 (4.0)	9.1 (5.3)		4.8 (10.6)	5.3 (8.5)	
**Extrintestinal**									
Extrintestinal			0.86			0.069			0.685
No	195.0 (80.7)	367.7 (81.4)		89.0 (86.5)	122.6 (70.6)		37.0 (81.7)	49.4 (78.5)	
Yes	46.6 (19.3)	84.1 (18.6)		13.9 (13.5)	51.0 (29.4)		8.3 (18.3)	13.6 (21.5)	
Oral ulcer			0.722			0.365			0.785
No	218.2 (90.3)	402.9 (89.2)		97.7 (95.0)	158.2 (91.1)		41.5 (91.7)	56.7 (90.0)	
Yes	23.5 (9.7)	48.9 (10.8)		5.2 (5.0)	15.4 (8.9)		3.7 (8.3)	6.3 (10.0)	
Liver/gallbladder			0.908			0.4			0.89
No	239.4 (99.1)	447.1 (99.0)		100.0 (97.2)	162.7 (93.7)		43.6 (96.3)	61.0 (96.8)	
Yes	2.2 (0.9)	4.6 (1.0)		2.8 (2.8)	10.9 (6.3)		1.7 (3.7)	2.0 (3.2)	
Skin			0.492			0.823			0.754
No	230.2 (95.2)	436.9 (96.7)		101.4 (98.6)	170.6 (98.3)		43.2 (95.5)	59.3 (94.2)	
Yes	11.5 (4.8)	14.9 (3.3)		1.4 (1.4)	3.0 (1.7)		2.0 (4.5)	3.6 (5.8)	
Eye			0.998			0.858			0.995
No	239.5 (99.1)	447.7 (99.1)		101.0 (98.1)	170.9 (98.5)		44.6 (98.5)	62.0 (98.5)	
Yes	2.2 (0.9)	4.1 (0.9)		1.9 (1.9)	2.7 (1.5)		0.7 (1.5)	1.0 (1.5)	
Osteoarthritis			0.862			0.521			0.425
No	232.5 (96.2)	436.0 (96.5)		100.4 (97.6)	171.1 (98.6)		44.4 (98.2)	60.3 (95.8)	
Yes	9.1 (3.8)	15.7 (3.5)		2.5 (2.4)	2.4 (1.4)		0.8 (1.8)	2.7 (4.2)	

Supplementary Table 11. Baseline characteristics of patients with and without biologic therapy across gradient boosting machine (GBM)-defined low-, intermediate-, and high-risk strata before inverse probability of treatment weighting in Imputed dataset 2

	Low-risk			Intermediate-risk			High-risk		
	No-Biologics	Biologics	*P*	No-Biologics	Biologics	*P*	No-Biologics	Biologics	*P*
	n =241	n =447		n =105	n =154		n =47	n =66	
**Demographic data**									
Gender (n, %)			0.063			0.134			1
Male	191 (79.3)	324 (72.5)		82 (78.1)	106 (68.8)		35 (74.5)	48 (72.7)	
Female	50 (20.7)	123 (27.5)		23 (21.9)	48 (31.2)		12 (25.5)	18 (27.3)	
Smoking (n, %)			0.001			0.064			0.326
No	182 (75.5)	385 (86.1)		74 (70.5)	125 (81.2)		30 (63.8)	49 (74.2)	
Yes	59 (24.5)	62 (13.9)		31 (29.5)	29 (18.8)		17 (36.2)	17 (25.8)	
BMI (kg/m^2)	19.38 (17.58, 21.97)	19.03 (17.30, 21.22)	0.067	17.71 (16.16, 20.07)	17.30 (16.05, 19.27)	0.085	17.92 (16.19, 19.05)	17.28 (15.96, 19.92)	0.468
**Laboratory data**									
WBC (10^9/L)	6.27 (5.23, 8.21)	6.44 (5.22, 8.08)	0.793	6.48 (4.62, 9.70)	6.26 (4.77, 8.18)	0.491	6.38 (4.68, 7.44)	6.64 (4.55, 9.45)	0.29
Hb (g/dL)	12.30 (10.80, 13.80)	12.10 (10.80, 13.70)	0.319	11.90 (10.10, 13.30)	11.90 (10.10, 13.60)	0.637	11.40 (8.85, 12.80)	10.15 (8.65, 11.95)	0.127
ESR (mm/h)	16.00 (7.00, 36.00)	20.00 (9.50, 40.00)	0.012	23.00 (7.00, 40.00)	25.00 (8.25, 47.00)	0.341	42.00 (18.50, 51.00)	37.50 (18.25, 59.00)	0.459
CRP (mg/dL)	1.15 (0.34, 2.49)	1.19 (0.42, 2.49)	0.427	2.85 (0.47, 6.10)	1.71 (0.45, 6.01)	0.378	6.48 (1.85, 9.05)	7.34 (1.98, 9.52)	0.389
TT (s)	16.50 (15.90, 17.30)	16.50 (15.75, 17.20)	0.88	16.40 (15.90, 17.10)	16.80 (16.00, 17.30)	0.077	16.90 (16.20, 17.90)	16.25 (15.20, 17.70)	0.026
APTT (s)	29.60 (27.90, 34.10)	29.60 (27.80, 32.40)	0.573	30.50 (27.90, 36.60)	29.85 (27.92, 33.12)	0.309	29.70 (28.70, 39.10)	30.15 (27.77, 35.67)	0.645
PT (s)	11.60 (11.00, 12.40)	11.60 (11.00, 12.70)	0.214	12.30 (11.30, 13.30)	12.19 (11.40, 13.28)	0.986	12.70 (11.95, 13.60)	12.60 (11.72, 13.90)	0.744
Fg (g/L)	3.77 (3.08, 4.32)	3.77 (3.02, 4.54)	0.622	3.34 (2.50, 4.89)	3.20 (2.39, 4.80)	0.755	3.40 (2.30, 4.65)	3.40 (2.48, 4.86)	0.87
DD (mg/L)	0.32 (0.22, 0.56)	0.31 (0.17, 0.54)	0.046	0.34 (0.17, 0.79)	0.37 (0.22, 0.88)	0.494	0.51 (0.26, 1.03)	0.87 (0.35, 1.61)	0.088
ALB (g/dL)	3.74 (3.34, 4.19)	3.79 (3.30, 4.17)	0.827	3.68 (3.19, 4.11)	3.62 (3.20, 4.19)	0.817	3.43 (2.88, 3.89)	3.29 (2.82, 3.80)	0.393
**Crohn's disease**									
Perianal abscess			0.004			0.001			0.311
No	168 (69.7)	261 (58.4)		86 (81.9)	95 (61.7)		35 (74.5)	42 (63.6)	
Yes	73 (30.3)	186 (41.6)		19 (18.1)	59 (38.3)		12 (25.5)	24 (36.4)	
Montreal A			<0.001			0.01			0.703
A1	20 (8.3)	83 (18.6)		5 (4.8)	16 (10.4)		4 (8.5)	5 (7.6)	
A2	182 (75.5)	326 (72.9)		74 (70.5)	120 (77.9)		34 (72.3)	52 (78.8)	

A3	39 (16.2)	38 (8.5)		26 (24.8)	18 (11.7)		9 (19.1)	9 (13.6)	
Montreal B			0.008			0.006			0.314
B1	121 (50.2)	278 (62.2)		16 (15.2)	50 (32.5)		3 (6.4)	5 (7.6)	
B2	60 (24.9)	79 (17.7)		57 (54.3)	72 (46.8)		36 (76.6)	42 (63.6)	
B3	60 (24.9)	90 (20.1)		32 (30.5)	32 (20.8)		8 (17.0)	19 (28.8)	
Montreal L			0.042			0.27			0.145
L1	73 (30.3)	95 (21.3)		33 (31.4)	33 (21.4)		18 (38.3)	16 (24.2)	
L2	14 (5.8)	24 (5.4)		8 (7.6)	12 (7.8)		1 (2.1)	8 (12.1)	
L3	149 (61.8)	322 (72.0)		64 (61.0)	108 (70.1)		27 (57.4)	41 (62.1)	
L4	5 (2.1)	6 (1.3)		0 (0.0)	1 (0.6)		1 (2.1)	1 (1.5)	
CDAI			0.104			0.851			0.735
Remission	38 (15.8)	63 (14.1)		12 (11.4)	16 (10.4)		1 (2.1)	3 (4.5)	
Mild active	131 (54.4)	210 (47.0)		40 (38.1)	63 (40.9)		14 (29.8)	24 (36.4)	
Moderate active	63 (26.1)	158 (35.3)		47 (44.8)	63 (40.9)		27 (57.4)	34 (51.5)	
Severe active	9 (3.7)	16 (3.6)		6 (5.7)	12 (7.8)		5 (10.6)	5 (7.6)	
**Extrintestinal**									
Extrintestinal			0.91			0.921			0.887
No	200 (83.0)	368 (82.3)		88 (83.8)	131 (85.1)		37 (78.7)	50 (75.8)	
Yes	41 (17.0)	79 (17.7)		17 (16.2)	23 (14.9)		10 (21.3)	16 (24.2)	
Oral ulcer			0.724			0.12			0.489
No	219 (90.9)	401 (89.7)		101 (96.2)	139 (90.3)		44 (93.6)	58 (87.9)	
Yes	22 (9.1)	46 (10.3)		4 (3.8)	15 (9.7)		3 (6.4)	8 (12.1)	
Liver/gallbladder			1			0.369			0.696
No	239 (99.2)	442 (98.9)		101 (96.2)	152 (98.7)		44 (93.6)	64 (97.0)	
Yes	2 (0.8)	5 (1.1)		4 (3.8)	2 (1.3)		3 (6.4)	2 (3.0)	
Skin			0.947			1			1
No	233 (96.7)	434 (97.1)		103 (98.1)	150 (97.4)		44 (93.6)	62 (93.9)	
Yes	8 (3.3)	13 (2.9)		2 (1.9)	4 (2.6)		3 (6.4)	4 (6.1)	
Eye			0.229			1			1
No	237 (98.3)	445 (99.6)		103 (98.1)	152 (98.7)		46 (97.9)	65 (98.5)	
Yes	4 (1.7)	2 (0.4)		2 (1.9)	2 (1.3)		1 (2.1)	1 (1.5)	
Osteoarthritis			1			0.195			0.591
No	232 (96.3)	430 (96.2)		100 (95.2)	152 (98.7)		46 (97.9)	62 (93.9)	
Yes	9 (3.7)	17 (3.8)		5 (4.8)	2 (1.3)		1 (2.1)	4 (6.1)	

Supplementary Table 12. Baseline characteristics of patients with and without biologic therapy across gradient boosting machine (GBM)-defined low-, intermediate-, and high-risk strata after inverse probability of treatment weighting in Imputed dataset 2

	Low-risk			Intermediate-risk			High-risk		
	No-Biologics	Biologics	*P*	No-Biologics	Biologics	*P*	No-Biologics	Biologics	*P*
	n =239.3	n =450.8		n =106.7	n =172.4		n =51	n =63.2	
**Demographic data**									
Gender (n, %)			0.612			0.805			0.491
Male	175.2 (73.2)	339.5 (75.3)		84.3 (79.0)	133.4 (77.4)		41.6 (81.5)	47.8 (75.6)	
Female	64.0 (26.8)	111.3 (24.7)		22.4 (21.0)	39.0 (22.6)		9.5 (18.5)	15.5 (24.4)	
Smoking (n, %)			0.931			0.188			0.684
No	195.1 (81.6)	369.0 (81.8)		81.6 (76.5)	109.9 (63.8)		38.2 (74.8)	44.7 (70.7)	
Yes	44.1 (18.4)	81.9 (18.2)		25.1 (23.5)	62.5 (36.2)		12.9 (25.2)	18.5 (29.3)	
BMI (kg/m^2)	18.78 (17.21, 21.33)	19.13 (17.30, 21.30)	0.408	17.30 (15.87, 19.50)	17.46 (16.36, 19.92)	0.403	16.69 (16.15, 18.34)	17.22 (15.94, 19.68)	0.977
**Laboratory data**									
WBC(10^9/L)	6.44 (5.26, 8.32)	6.44 (5.19, 8.07)	0.574	6.92 (4.51, 9.16)	6.35 (5.03, 9.28)	0.715	6.32 (5.06, 7.27)	6.31 (4.35, 9.40)	0.99
Hb (g/dL)	12.30 (10.70, 13.64)	12.20 (10.80, 13.70)	0.703	12.01 (10.30, 13.16)	11.90 (9.30, 13.60)	0.889	10.12 (8.03, 12.34)	10.38 (9.01, 11.90)	0.666
ESR (mm/h)	16.00 (7.00, 36.00)	18.00 (9.00, 38.00)	0.283	30.00 (9.00, 42.87)	30.95 (9.52, 49.68)	0.374	39.80 (31.00, 47.95)	35.37 (14.26, 58.01)	0.657
CRP (mg/dL)	1.15 (0.37, 2.72)	1.16 (0.39, 2.52)	0.85	1.69 (0.49, 5.74)	1.73 (0.62, 7.09)	0.392	7.38 (4.17, 9.09)	6.76 (1.33, 9.32)	0.405
TT (s)	16.50 (15.90, 17.30)	16.50 (15.80, 17.20)	0.786	16.69 (16.00, 17.00)	16.74 (15.64, 17.50)	0.693	16.43 (15.75, 17.17)	16.70 (15.40, 17.94)	0.896
APTT (s)	29.38 (27.90, 33.67)	29.60 (27.90, 32.50)	0.714	28.63 (27.31, 33.75)	30.50 (28.01, 33.46)	0.214	29.76 (28.73, 40.56)	30.20 (28.01, 35.48)	0.511
PT (s)	11.70 (11.00, 12.48)	11.60 (11.00, 12.60)	0.89	11.64 (10.98, 12.82)	12.20 (11.40, 12.80)	0.078	12.81 (12.07, 14.40)	12.40 (11.48, 13.70)	0.322
Fg (g/L)	3.80 (3.08, 4.34)	3.73 (3.02, 4.49)	0.991	3.70 (2.52, 4.43)	3.59 (2.41, 4.99)	0.645	3.32 (3.08, 4.33)	3.35 (2.35, 4.85)	0.901
DD (mg/L)	0.30 (0.22, 0.55)	0.31 (0.17, 0.54)	0.15	0.28 (0.13, 0.67)	0.37 (0.16, 0.95)	0.392	0.76 (0.30, 0.93)	0.77 (0.26, 1.50)	0.649
ALB (g/dL)	3.77 (3.34, 4.19)	3.78 (3.30, 4.15)	0.877	3.76 (3.14, 4.13)	3.63 (3.00, 4.09)	0.422	3.58 (2.88, 3.78)	3.33 (2.86, 3.83)	0.621
**Crohn's disease**									
Perianal abscess			0.958			0.913			0.717
No	151.0 (63.1)	283.4 (62.9)		77.1 (72.2)	126.2 (73.2)		36.1 (70.7)	42.1 (66.6)	
Yes	88.3 (36.9)	167.4 (37.1)		29.6 (27.8)	46.2 (26.8)		14.9 (29.3)	21.1 (33.4)	
Montreal A			0.979			0.097			0.945
A1	36.9 (15.4)	66.8 (14.8)		21.0 (19.6)	12.8 (7.4)		3.9 (7.7)	4.6 (7.3)	
A2	176.5 (73.8)	334.3 (74.1)		69.5 (65.2)	109.8 (63.7)		41.4 (81.2)	50.2 (79.4)	

A3	25.9 (10.8)	49.7 (11.0)		16.2 (15.2)	49.7 (28.8)		5.7 (11.1)	8.4 (13.3)	
Montreal B			0.986			0.761			0.836
B1	135.4 (56.6)	257.7 (57.2)		25.3 (23.7)	38.1 (22.1)		4.8 (9.4)	5.1 (8.0)	
B2	48.8 (20.4)	92.1 (20.4)		59.1 (55.4)	87.5 (50.7)		35.6 (69.7)	41.3 (65.3)	
B3	55.0 (23.0)	101.0 (22.4)		22.3 (20.9)	46.9 (27.2)		10.7 (20.9)	16.9 (26.8)	
Montreal L			0.982			0.481			0.659
L1	60.4 (25.2)	114.4 (25.4)		24.5 (23.0)	37.4 (21.7)		14.3 (28.0)	20.1 (31.8)	
L2	14.1 (5.9)	24.4 (5.4)		13.6 (12.8)	11.2 (6.5)		9.5 (18.6)	5.5 (8.7)	
L3	161.2 (67.4)	303.5 (67.3)		68.5 (64.2)	123.2 (71.4)		26.5 (51.9)	36.6 (57.9)	
L4	3.6 (1.5)	8.5 (1.9)		0.0 (0.0)	0.6 (0.3)		0.7 (1.4)	1.0 (1.6)	
CDAI			0.982			0.833			0.895
Remission	33.9 (14.2)	65.4 (14.5)		10.4 (9.7)	15.5 (9.0)		1.3 (2.6)	2.5 (3.9)	
Mild active	117.7 (49.2)	226.7 (50.3)		49.9 (46.8)	70.8 (41.1)		21.0 (41.1)	21.2 (33.6)	
Moderate active	79.8 (33.3)	143.1 (31.7)		41.9 (39.2)	76.9 (44.6)		24.6 (48.2)	34.3 (54.3)	
Severe active	7.9 (3.3)	15.7 (3.5)		4.5 (4.2)	9.2 (5.3)		4.1 (8.0)	5.2 (8.2)	
**Extrintestinal**									
Extrintestinal			0.62			0.075			0.53
No	191.2 (79.9)	368.9 (81.8)		93.1 (87.2)	123.3 (71.5)		42.8 (83.9)	49.9 (78.9)	
Yes	48.1 (20.1)	82.0 (18.2)		13.6 (12.8)	49.1 (28.5)		8.2 (16.1)	13.4 (21.1)	
Oral ulcer			0.784			0.637			0.68
No	217.0 (90.7)	405.7 (90.0)		101.3 (95.0)	160.8 (93.3)		47.4 (92.8)	57.2 (90.4)	
Yes	22.3 (9.3)	45.2 (10.0)		5.4 (5.0)	11.6 (6.7)		3.7 (7.2)	6.1 (9.6)	
Liver/gallbladder			0.784			0.203			0.98
No	236.1 (98.7)	446.1 (98.9)		103.7 (97.2)	155.8 (90.3)		49.3 (96.7)	61.1 (96.6)	
Yes	3.2 (1.3)	4.8 (1.1)		3.0 (2.8)	16.6 (9.7)		1.7 (3.3)	2.1 (3.4)	
Skin			0.42			0.759			0.68
No	226.5 (94.7)	435.5 (96.6)		105.3 (98.7)	169.4 (98.3)		49.0 (96.0)	59.7 (94.4)	
Yes	12.8 (5.3)	15.3 (3.4)		1.4 (1.3)	3.0 (1.7)		2.1 (4.0)	3.5 (5.6)	
Eye			0.854			0.966			0.93
No	237.1 (99.1)	446.0 (98.9)		105.4 (98.8)	170.3 (98.8)		50.3 (98.7)	62.3 (98.5)	
Yes	2.2 (0.9)	4.9 (1.1)		1.3 (1.2)	2.1 (1.2)		0.7 (1.3)	0.9 (1.5)	
Osteoarthritis			0.961			0.473			0.35
No	230.6 (96.4)	434.9 (96.5)		104.2 (97.6)	170.2 (98.7)		50.2 (98.5)	60.6 (95.8)	
Yes	8.6 (3.6)	15.9 (3.5)		2.5 (2.4)	2.2 (1.3)		0.8 (1.5)	2.6 (4.2)	

Supplementary Table 13. Baseline characteristics of patients with and without biologic therapy across gradient boosting machine (GBM)-defined low-, intermediate-, and high-risk strata before inverse probability of treatment weighting in Imputed dataset 3

	Low-risk			Intermediate-risk			High-risk		
	No-Biologics	Biologics	*P*	No-Biologics	Biologics	*P*	No-Biologics	Biologics	*P*
	n =241	n =447		n =105	n =154		n =47	n =66	
**Demographic data**									
Gender (n, %)			0.063			0.134			1
Male	191 (79.3)	324 (72.5)		82 (78.1)	106 (68.8)		35 (74.5)	48 (72.7)	
Female	50 (20.7)	123 (27.5)		23 (21.9)	48 (31.2)		12 (25.5)	18 (27.3)	
Smoking (n, %)			<0.001			0.059			0.244
No	179 (74.3)	386 (86.4)		73 (69.5)	124 (80.5)		30 (63.8)	50 (75.8)	
Yes	62 (25.7)	61 (13.6)		32 (30.5)	30 (19.5)		17 (36.2)	16 (24.2)	
BMI (kg/m^2)	19.26 (17.36, 22.21)	19.05 (17.30, 21.24)	0.076	17.71 (16.16, 20.07)	17.30 (16.14, 19.27)	0.119	17.93 (16.33, 19.30)	17.30 (15.96, 20.04)	0.3
**Laboratory data**									
WBC(10^9/L)	6.26 (5.21, 8.21)	6.42 (5.20, 8.07)	0.66	6.37 (4.50, 9.70)	6.26 (4.73, 8.18)	0.654	6.44 (4.68, 7.58)	6.44 (4.48, 9.45)	0.582
Hb (g/dL)	12.40 (10.80, 13.80)	12.20 (10.75, 13.70)	0.295	11.90 (9.90, 13.30)	11.90 (10.10, 13.60)	0.668	11.70 (9.25, 12.80)	10.50 (8.65, 12.38)	0.097
ESR (mm/h)	16.00 (7.00, 34.00)	19.00 (10.00, 40.00)	0.008	23.00 (6.00, 40.00)	25.00 (8.00, 47.00)	0.29	43.00 (18.50, 50.50)	36.00 (19.00, 59.00)	0.616
CRP (mg/dL)	1.15 (0.35, 2.40)	1.29 (0.45, 2.40)	0.431	2.48 (0.47, 6.44)	1.73 (0.45, 6.30)	0.372	6.48 (1.73, 9.05)	7.53 (2.36, 10.33)	0.14
TT (s)	16.40 (15.80, 17.20)	16.50 (15.75, 17.20)	0.992	16.50 (15.80, 17.00)	16.80 (16.00, 17.20)	0.111	16.90 (16.10, 17.65)	16.30 (15.20, 17.70)	0.031
APTT (s)	29.60 (27.90, 34.10)	29.60 (27.65, 32.40)	0.421	30.60 (28.00, 36.40)	29.90 (27.42, 33.35)	0.216	30.00 (28.90, 38.50)	30.15 (27.85, 35.88)	0.692
PT (s)	11.60 (11.00, 12.40)	11.70 (11.00, 12.90)	0.25	12.30 (11.20, 13.30)	12.15 (11.40, 13.30)	0.724	12.70 (12.00, 13.70)	12.60 (11.83, 13.85)	0.832
Fg (g/L)	3.77 (3.08, 4.32)	3.80 (3.02, 4.58)	0.421	3.40 (2.50, 4.89)	3.20 (2.39, 4.80)	0.689	3.40 (2.38, 4.65)	3.40 (2.49, 4.81)	0.995
DD (mg/L)	0.32 (0.20, 0.56)	0.31 (0.17, 0.54)	0.06	0.38 (0.19, 0.79)	0.37 (0.22, 0.88)	0.709	0.52 (0.26, 1.03)	0.87 (0.38, 1.62)	0.059
ALB (g/dL)	3.76 (3.32, 4.19)	3.78 (3.32, 4.15)	0.833	3.59 (3.16, 4.10)	3.74 (3.20, 4.21)	0.241	3.58 (3.05, 3.92)	3.24 (2.78, 3.80)	0.055
**Crohn's disease**									
Perianal abscess			0.004			0.001			0.311
No	168 (69.7)	261 (58.4)		86 (81.9)	95 (61.7)		35 (74.5)	42 (63.6)	
Yes	73 (30.3)	186 (41.6)		19 (18.1)	59 (38.3)		12 (25.5)	24 (36.4)	
Montreal A			<0.001			0.01			0.703
A1	20 (8.3)	83 (18.6)		5 (4.8)	16 (10.4)		4 (8.5)	5 (7.6)	
A2	182 (75.5)	326 (72.9)		74 (70.5)	120 (77.9)		34 (72.3)	52 (78.8)	

A3	39 (16.2)	38 (8.5)		26 (24.8)	18 (11.7)		9 (19.1)	9 (13.6)	
Montreal B			0.008			0.006			0.314
B1	121 (50.2)	278 (62.2)		16 (15.2)	50 (32.5)		3 (6.4)	5 (7.6)	
B2	60 (24.9)	79 (17.7)		57 (54.3)	72 (46.8)		36 (76.6)	42 (63.6)	
B3	60 (24.9)	90 (20.1)		32 (30.5)	32 (20.8)		8 (17.0)	19 (28.8)	
Montreal L			0.042			0.27			0.145
L1	73 (30.3)	95 (21.3)		33 (31.4)	33 (21.4)		18 (38.3)	16 (24.2)	
L2	14 (5.8)	24 (5.4)		8 (7.6)	12 (7.8)		1 (2.1)	8 (12.1)	
L3	149 (61.8)	322 (72.0)		64 (61.0)	108 (70.1)		27 (57.4)	41 (62.1)	
L4	5 (2.1)	6 (1.3)		0 (0.0)	1 (0.6)		1 (2.1)	1 (1.5)	
CDAI			0.104			0.851			0.735
Remission	38 (15.8)	63 (14.1)		12 (11.4)	16 (10.4)		1 (2.1)	3 (4.5)	
Mild active	131 (54.4)	210 (47.0)		40 (38.1)	63 (40.9)		14 (29.8)	24 (36.4)	
Moderate active	63 (26.1)	158 (35.3)		47 (44.8)	63 (40.9)		27 (57.4)	34 (51.5)	
Severe active	9 (3.7)	16 (3.6)		6 (5.7)	12 (7.8)		5 (10.6)	5 (7.6)	
**Extrintestinal**									
Extrintestinal			0.91			0.921			0.887
No	200 (83.0)	368 (82.3)		88 (83.8)	131 (85.1)		37 (78.7)	50 (75.8)	
Yes	41 (17.0)	79 (17.7)		17 (16.2)	23 (14.9)		10 (21.3)	16 (24.2)	
Oral ulcer			0.724			0.12			0.489
No	219 (90.9)	401 (89.7)		101 (96.2)	139 (90.3)		44 (93.6)	58 (87.9)	
Yes	22 (9.1)	46 (10.3)		4 (3.8)	15 (9.7)		3 (6.4)	8 (12.1)	
Liver/gallbladder			1			0.369			0.696
No	239 (99.2)	442 (98.9)		101 (96.2)	152 (98.7)		44 (93.6)	64 (97.0)	
Yes	2 (0.8)	5 (1.1)		4 (3.8)	2 (1.3)		3 (6.4)	2 (3.0)	
Skin			0.947			1			1
No	233 (96.7)	434 (97.1)		103 (98.1)	150 (97.4)		44 (93.6)	62 (93.9)	
Yes	8 (3.3)	13 (2.9)		2 (1.9)	4 (2.6)		3 (6.4)	4 (6.1)	
Eye			0.229			1			1
No	237 (98.3)	445 (99.6)		103 (98.1)	152 (98.7)		46 (97.9)	65 (98.5)	
Yes	4 (1.7)	2 (0.4)		2 (1.9)	2 (1.3)		1 (2.1)	1 (1.5)	
Osteoarthritis			1			0.195			0.591
No	232 (96.3)	430 (96.2)		100 (95.2)	152 (98.7)		46 (97.9)	62 (93.9)	
Yes	9 (3.7)	17 (3.8)		5 (4.8)	2 (1.3)		1 (2.1)	4 (6.1)	

Supplementary Table 14. Baseline characteristics of patients with and without biologic therapy across gradient boosting machine (GBM)-defined low-, intermediate-, and high-risk strata after inverse probability of treatment weighting in Imputed dataset 3

	Low-risk			Intermediate-risk			High-risk		
	No-Biologics	Biologics	*P*	No-Biologics	Biologics	*P*	No-Biologics	Biologics	*P*
	n =239.2	n =451.3		n =102.8	n =165.1		n =53.8	n =64.1	
**Demographic data**									
Gender (n, %)			0.66						
Male	176.5 (73.8)	341.0 (75.5)		77.8 (75.7)	124.8 (75.6)	0.985	44.5 (82.8)	47.9 (74.6)	0.357
Female	62.8 (26.2)	110.4 (24.5)		25.0 (24.3)	40.4 (24.4)		9.3 (17.2)	16.3 (25.4)	
Smoking (n, %)			0.885						
No	193.9 (81.1)	368.0 (81.5)		75.6 (73.5)	109.6 (66.4)	0.434	41.5 (77.2)	46.1 (71.9)	0.599
Yes	45.3 (18.9)	83.3 (18.5)		27.2 (26.5)	55.5 (33.6)		12.3 (22.8)	18.1 (28.1)	
BMI (kg/m^2)	18.77 (17.16, 21.45)	19.13 (17.30, 21.31)	0.417	17.19 (15.88, 19.58)	17.65 (16.36, 19.90)	0.363	16.69 (16.26, 18.33)	17.27 (15.94, 19.74)	0.894
**Laboratory data**									
WBC(10^9/L)	6.42 (5.26, 8.30)	6.42 (5.19, 8.07)	0.615	6.49 (4.48, 8.81)	6.35 (4.82, 9.24)	0.523	6.13 (4.80, 7.11)	6.00 (4.35, 9.45)	0.944
Hb (g/dL)	12.30 (10.61, 13.71)	12.20 (10.80, 13.72)	0.843	12.10 (10.10, 13.30)	11.90 (9.80, 13.60)	0.777	9.91 (8.00, 12.19)	10.80 (9.17, 12.50)	0.434
ESR (mm/h)	16.00 (7.00, 35.00)	18.00 (9.00, 37.00)	0.352	24.00 (6.00, 43.48)	27.00 (8.00, 59.00)	0.319	39.53 (31.21, 48.69)	35.58 (14.00, 58.67)	0.773
CRP (mg/dL)	1.09 (0.35, 2.39)	1.29 (0.43, 2.45)	0.411	2.25 (0.47, 6.06)	1.79 (0.65, 7.06)	0.662	7.71 (5.21, 9.09)	6.80 (1.53, 9.32)	0.491
TT (s)	16.50 (15.80, 17.20)	16.50 (15.78, 17.20)	0.828	16.70 (15.90, 17.00)	16.70 (15.90, 17.30)	0.731	16.20 (15.79, 17.04)	16.70 (15.40, 18.08)	0.663
APTT (s)	29.39 (27.50, 33.47)	29.60 (27.77, 32.63)	0.609	29.20 (27.33, 33.73)	30.27 (27.57, 33.48)	0.418	30.97 (29.26, 31.63)	30.18 (28.16, 35.76)	0.72
PT (s)	11.70 (11.00, 12.57)	11.70 (11.00, 12.80)	0.691	11.70 (11.10, 12.90)	12.18 (11.40, 13.10)	0.145	13.12 (12.24, 13.90)	12.44 (11.60, 13.66)	0.302
Fg (g/L)	3.80 (3.02, 4.34)	3.77 (3.00, 4.52)	0.879	3.58 (2.50, 4.51)	3.50 (2.40, 4.89)	0.658	3.94 (2.86, 4.12)	3.30 (2.38, 4.80)	0.581
DD (mg/L)	0.31 (0.20, 0.56)	0.31 (0.17, 0.55)	0.186	0.32 (0.14, 0.68)	0.39 (0.20, 0.95)	0.439	0.94 (0.30, 1.72)	0.77 (0.25, 1.47)	0.55
ALB (g/dL)	3.78 (3.37, 4.19)	3.78 (3.32, 4.14)	0.749	3.69 (3.15, 4.13)	3.72 (3.05, 4.14)	0.591	3.68 (3.02, 3.81)	3.37 (2.86, 3.84)	0.499
**Crohn's disease**									
Perianal abscess			0.982			0.873			0.691
No	150.9 (63.1)	284.2 (63.0)		72.7 (70.7)	119.0 (72.1)		39.0 (72.5)	43.5 (67.9)	
Yes	88.3 (36.9)	167.1 (37.0)		30.1 (29.3)	46.1 (27.9)		14.8 (27.5)	20.6 (32.1)	
Montreal A			0.952			0.321			0.91
A1	37.4 (15.6)	67.0 (14.8)		14.8 (14.4)	12.6 (7.6)		3.3 (6.1)	4.4 (6.8)	
A2	175.4 (73.3)	336.1 (74.5)		71.2 (69.2)	109.4 (66.2)		44.6 (82.9)	51.2 (79.7)	

A3	26.5 (11.1)	48.2 (10.7)		16.8 (16.4)	43.2 (26.2)		5.9 (11.1)	8.6 (13.4)	
Montreal B			0.978			0.638			0.841
B1	135.6 (56.7)	255.7 (56.7)		20.4 (19.9)	38.0 (23.0)		4.8 (9.0)	5.2 (8.0)	
B2	48.6 (20.3)	94.5 (20.9)		59.3 (57.7)	81.8 (49.5)		38.1 (70.8)	42.2 (65.8)	
B3	55.0 (23.0)	101.1 (22.4)		23.1 (22.4)	45.4 (27.5)		10.9 (20.2)	16.8 (26.2)	
Montreal L			0.986			0.925			0.416
L1	60.2 (25.2)	117.2 (26.0)		24.3 (23.6)	37.2 (22.5)		14.9 (27.7)	20.4 (31.8)	
L2	14.3 (6.0)	24.6 (5.5)		6.6 (6.4)	11.3 (6.9)		12.8 (23.9)	5.4 (8.4)	
L3	161.4 (67.4)	302.3 (67.0)		71.9 (70.0)	116.0 (70.3)		25.3 (47.0)	37.3 (58.1)	
L4	3.3 (1.4)	7.1 (1.6)		0.0 (0.0)	0.6 (0.4)		0.8 (1.4)	1.1 (1.7)	
CDAI			0.991			0.922			0.816
Remission	34.6 (14.5)	64.1 (14.2)		11.6 (11.3)	14.7 (8.9)		1.4 (2.6)	2.6 (4.0)	
Mild active	118.7 (49.6)	229.3 (50.8)		43.7 (42.5)	68.7 (41.6)		23.0 (42.8)	20.9 (32.5)	
Moderate active	77.9 (32.6)	142.2 (31.5)		43.3 (42.1)	72.6 (44.0)		24.9 (46.4)	35.0 (54.6)	
Severe active	8.0 (3.3)	15.7 (3.5)		4.3 (4.2)	9.0 (5.5)		4.4 (8.2)	5.7 (8.9)	
**Extrintestinal**									
Extrintestinal			0.685			0.123			0.437
No	193.5 (80.9)	371.8 (82.4)		89.4 (87.0)	124.1 (75.1)		46.5 (86.4)	51.7 (80.6)	
Yes	45.7 (19.1)	79.5 (17.6)		13.4 (13.0)	41.1 (24.9)		7.3 (13.6)	12.5 (19.4)	
Oral ulcer			0.741			0.5			0.477
No	217.6 (91.0)	406.8 (90.1)		97.8 (95.2)	153.4 (92.9)		50.8 (94.5)	58.3 (90.8)	
Yes	21.6 (9.0)	44.5 (9.9)		5.0 (4.8)	11.8 (7.1)		3.0 (5.5)	5.9 (9.2)	
Liver/gallbladder			0.975			0.27			0.967
No	236.7 (98.9)	446.6 (99.0)		99.9 (97.2)	151.7 (91.9)		52.2 (97.0)	62.3 (97.2)	
Yes	2.6 (1.1)	4.7 (1.0)		2.9 (2.8)	13.4 (8.1)		1.6 (3.0)	1.8 (2.8)	
Skin			0.375			0.742			0.69
No	226.8 (94.8)	436.8 (96.8)		101.4 (98.6)	162.1 (98.2)		51.8 (96.4)	60.9 (95.0)	
Yes	12.4 (5.2)	14.5 (3.2)		1.4 (1.4)	3.0 (1.8)		2.0 (3.6)	3.2 (5.0)	
Eye			0.973			0.904			0.91
No	237.1 (99.1)	447.2 (99.1)		101.2 (98.4)	162.8 (98.6)		53.2 (98.9)	63.3 (98.7)	
Yes	2.1 (0.9)	4.1 (0.9)		1.6 (1.6)	2.3 (1.4)		0.6 (1.1)	0.8 (1.3)	
Osteoarthritis			0.982			0.498			0.359
No	231.0 (96.6)	435.6 (96.5)		100.3 (97.6)	162.9 (98.6)		53.0 (98.5)	61.5 (95.8)	
Yes	8.3 (3.4)	15.7 (3.5)		2.5 (2.4)	2.3 (1.4)		0.8 (1.5)	2.7 (4.2)	

Supplementary Table 15. Baseline characteristics of patients with and without biologic therapy across gradient boosting machine (GBM)-defined low-, intermediate-, and high-risk strata before inverse probability of treatment weighting in Imputed dataset 4

	Low-risk			Intermediate-risk			High-risk		
	No-Biologics	Biologics	*P*	No-Biologics	Biologics	*P*	No-Biologics	Biologics	*P*
	n =241	n =447		n =105	n =154		n =47	n =66	
**Demographic data**									
Gender (n, %)			0.063			0.134			1
Male	191 (79.3)	324 (72.5)		82 (78.1)	106 (68.8)		35 (74.5)	48 (72.7)	
Female	50 (20.7)	123 (27.5)		23 (21.9)	48 (31.2)		12 (25.5)	18 (27.3)	
Smoking (n, %)			<0.001			0.04			0.423
No	180 (74.7)	384 (85.9)		72 (68.6)	124 (80.5)		30 (63.8)	48 (72.7)	
Yes	61 (25.3)	63 (14.1)		33 (31.4)	30 (19.5)		17 (36.2)	18 (27.3)	
BMI (kg/m^2)	19.26 (17.36, 21.88)	19.05 (17.30, 21.22)	0.116	17.71 (16.16, 20.07)	17.30 (16.14, 19.38)	0.138	17.31 (16.32, 18.95)	17.28 (15.96, 19.92)	0.445
**Laboratory data**									
WBC(10^9/L)	6.22 (5.14, 8.21)	6.51 (5.22, 8.09)	0.382	6.37 (4.54, 9.70)	6.33 (4.77, 8.31)	0.66	6.44 (4.68, 7.46)	6.70 (4.55, 9.74)	0.225
Hb (g/dL)	12.30 (10.80, 13.80)	12.20 (10.75, 13.65)	0.523	11.90 (9.90, 13.30)	11.90 (9.95, 13.60)	0.678	11.40 (9.40, 12.80)	10.00 (8.60, 11.57)	0.035
ESR (mm/h)	15.00 (7.00, 34.00)	19.00 (10.50, 40.00)	0.001	23.00 (6.00, 40.00)	24.00 (9.00, 46.75)	0.245	43.00 (17.00, 52.50)	37.00 (19.25, 62.00)	0.566
CRP (mg/dL)	1.15 (0.35, 2.49)	1.16 (0.40, 2.45)	0.827	2.85 (0.47, 6.44)	1.77 (0.42, 6.56)	0.625	6.59 (1.85, 9.13)	7.34 (1.98, 9.97)	0.413
TT (s)	16.50 (15.90, 17.20)	16.60 (15.80, 17.20)	0.864	16.60 (15.90, 17.10)	16.80 (16.02, 17.20)	0.252	16.90 (16.16, 17.65)	16.30 (15.20, 17.70)	0.046
APTT (s)	29.60 (27.90, 34.10)	29.60 (27.70, 32.45)	0.469	30.50 (28.00, 36.10)	29.90 (27.68, 32.85)	0.276	29.80 (28.70, 39.10)	30.20 (27.95, 35.17)	0.606
PT (s)	11.60 (11.00, 12.40)	11.70 (11.00, 12.70)	0.199	12.30 (11.30, 13.30)	12.15 (11.40, 13.28)	0.924	12.70 (11.95, 13.70)	12.60 (11.70, 13.85)	0.813
Fg (g/L)	3.74 (3.10, 4.31)	3.80 (3.05, 4.58)	0.303	3.34 (2.50, 4.89)	3.20 (2.39, 4.80)	0.646	3.93 (2.38, 4.69)	3.40 (2.76, 4.81)	0.843
DD (mg/L)	0.32 (0.20, 0.56)	0.31 (0.18, 0.56)	0.209	0.34 (0.17, 0.80)	0.34 (0.21, 0.80)	0.894	0.48 (0.26, 1.03)	0.85 (0.35, 1.61)	0.105
ALB (g/dL)	3.76 (3.36, 4.17)	3.77 (3.31, 4.15)	0.853	3.67 (3.19, 4.10)	3.74 (3.21, 4.20)	0.423	3.49 (3.02, 3.91)	3.26 (2.81, 3.71)	0.085
**Crohn's disease**									
Perianal abscess			0.004			0.001			0.311
No	168 (69.7)	261 (58.4)		86 (81.9)	95 (61.7)		35 (74.5)	42 (63.6)	
Yes	73 (30.3)	186 (41.6)		19 (18.1)	59 (38.3)		12 (25.5)	24 (36.4)	
Montreal A			<0.001			0.01			0.703
A1	20 (8.3)	83 (18.6)		5 (4.8)	16 (10.4)		4 (8.5)	5 (7.6)	
A2	182 (75.5)	326 (72.9)		74 (70.5)	120 (77.9)		34 (72.3)	52 (78.8)	

A3	39 (16.2)	38 (8.5)		26 (24.8)	18 (11.7)		9 (19.1)	9 (13.6)	
Montreal B			0.008			0.006			0.314
B1	121 (50.2)	278 (62.2)		16 (15.2)	50 (32.5)		3 (6.4)	5 (7.6)	
B2	60 (24.9)	79 (17.7)		57 (54.3)	72 (46.8)		36 (76.6)	42 (63.6)	
B3	60 (24.9)	90 (20.1)		32 (30.5)	32 (20.8)		8 (17.0)	19 (28.8)	
Montreal L			0.042			0.27			0.145
L1	73 (30.3)	95 (21.3)		33 (31.4)	33 (21.4)		18 (38.3)	16 (24.2)	
L2	14 (5.8)	24 (5.4)		8 (7.6)	12 (7.8)		1 (2.1)	8 (12.1)	
L3	149 (61.8)	322 (72.0)		64 (61.0)	108 (70.1)		27 (57.4)	41 (62.1)	
L4	5 (2.1)	6 (1.3)		0 (0.0)	1 (0.6)		1 (2.1)	1 (1.5)	
CDAI			0.104			0.851			0.735
Remission	38 (15.8)	63 (14.1)		12 (11.4)	16 (10.4)		1 (2.1)	3 (4.5)	
Mild active	131 (54.4)	210 (47.0)		40 (38.1)	63 (40.9)		14 (29.8)	24 (36.4)	
Moderate active	63 (26.1)	158 (35.3)		47 (44.8)	63 (40.9)		27 (57.4)	34 (51.5)	
Severe active	9 (3.7)	16 (3.6)		6 (5.7)	12 (7.8)		5 (10.6)	5 (7.6)	
**Extrintestinal**									
Extrintestinal			0.91			0.921			0.887
No	200 (83.0)	368 (82.3)		88 (83.8)	131 (85.1)		37 (78.7)	50 (75.8)	
Yes	41 (17.0)	79 (17.7)		17 (16.2)	23 (14.9)		10 (21.3)	16 (24.2)	
Oral ulcer			0.724			0.12			0.489
No	219 (90.9)	401 (89.7)		101 (96.2)	139 (90.3)		44 (93.6)	58 (87.9)	
Yes	22 (9.1)	46 (10.3)		4 (3.8)	15 (9.7)		3 (6.4)	8 (12.1)	
Liver/gallbladder			1			0.369			0.696
No	239 (99.2)	442 (98.9)		101 (96.2)	152 (98.7)		44 (93.6)	64 (97.0)	
Yes	2 (0.8)	5 (1.1)		4 (3.8)	2 (1.3)		3 (6.4)	2 (3.0)	
Skin			0.947			1			1
No	233 (96.7)	434 (97.1)		103 (98.1)	150 (97.4)		44 (93.6)	62 (93.9)	
Yes	8 (3.3)	13 (2.9)		2 (1.9)	4 (2.6)		3 (6.4)	4 (6.1)	
Eye			0.229			1			1
No	237 (98.3)	445 (99.6)		103 (98.1)	152 (98.7)		46 (97.9)	65 (98.5)	
Yes	4 (1.7)	2 (0.4)		2 (1.9)	2 (1.3)		1 (2.1)	1 (1.5)	
Osteoarthritis			1			0.195			0.591
No	232 (96.3)	430 (96.2)		100 (95.2)	152 (98.7)		46 (97.9)	62 (93.9)	
Yes	9 (3.7)	17 (3.8)		5 (4.8)	2 (1.3)		1 (2.1)	4 (6.1)	

Supplementary Table 16. Baseline characteristics of patients with and without biologic therapy across gradient boosting machine (GBM)-defined low-, intermediate-, and high-risk strata after inverse probability of treatment weighting in Imputed dataset 4

	Low-risk			Intermediate-risk			High-risk		
	No-Biologics	Biologics	*P*	No-Biologics	Biologics	*P*	No-Biologics	Biologics	*P*
	n =241.7	n =451.7		n =102.9	n =173.6		n =45.2	n =63	
**Demographic data**									
Gender (n, %)			0.982			0.977			0.567
Male	183.8 (76.0)	343.1 (75.9)		79.6 (77.3)	133.9 (77.2)		36.1 (79.7)	47.2 (74.9)	
Female	57.9 (24.0)	108.7 (24.1)		23.3 (22.7)	39.7 (22.8)		9.2 (20.3)	15.8 (25.1)	
Smoking (n, %)			0.983			0.213			1
No	196.3 (81.2)	367.2 (81.3)		76.7 (74.5)	108.6 (62.6)		32.2 (71.1)	44.8 (71.1)	
Yes	45.4 (18.8)	84.6 (18.7)		26.2 (25.5)	65.0 (37.4)		13.1 (28.9)	18.2 (28.9)	
BMI (kg/m^2)	18.73 (17.21, 21.35)	19.10 (17.30, 21.26)	0.467	17.30 (15.87, 19.51)	17.30 (16.40, 19.84)	0.46	17.26 (15.92, 18.75)	17.23 (15.94, 19.70)	0.973
**Laboratory data**									
WBC (10^9/L)	6.46 (5.26, 8.33)	6.43 (5.19, 8.06)	0.462	6.89 (4.53, 9.20)	6.33 (5.04, 8.40)	0.95	6.76 (5.10, 7.48)	6.03 (4.35, 9.45)	0.914
Hb (g/dL)	12.30 (10.36, 13.60)	12.20 (10.80, 13.70)	0.933	12.10 (10.30, 13.30)	11.72 (9.23, 13.60)	0.509	10.81 (8.68, 12.38)	10.38 (8.91, 11.76)	0.604
ESR (mm/h)	16.00 (7.00, 37.34)	18.00 (9.00, 38.00)	0.468	24.33 (7.00, 43.09)	27.00 (9.00, 44.37)	0.445	43.70 (23.13, 51.00)	36.00 (17.51, 58.86)	0.894
CRP (mg/dL)	1.09 (0.41, 2.24)	1.14 (0.35, 2.48)	0.791	2.52 (0.46, 6.46)	1.72 (0.57, 7.27)	0.708	7.18 (5.25, 9.07)	6.99 (1.57, 9.33)	0.723
TT (s)	16.50 (15.90, 17.21)	16.60 (15.80, 17.20)	0.767	16.70 (16.11, 17.10)	16.70 (15.60, 17.20)	0.971	16.70 (16.10, 17.30)	16.70 (15.40, 17.91)	0.702
APTT (s)	29.40 (27.84, 33.53)	29.60 (27.70, 32.70)	0.809	29.20 (27.50, 33.19)	30.20 (27.90, 32.69)	0.397	29.53 (28.32, 35.29)	30.28 (28.80, 35.24)	0.503
PT (s)	11.70 (11.10, 12.54)	11.60 (11.00, 12.70)	0.927	11.73 (11.13, 12.90)	12.15 (11.40, 12.80)	0.161	12.62 (11.67, 13.37)	12.42 (11.52, 13.70)	0.848
Fg (g/L)	3.80 (3.10, 4.41)	3.80 (3.03, 4.54)	0.94	3.34 (2.50, 4.41)	3.50 (2.40, 4.80)	0.562	3.94 (2.74, 4.43)	3.40 (2.48, 4.85)	0.678
DD (mg/L)	0.31 (0.22, 0.56)	0.31 (0.18, 0.55)	0.204	0.28 (0.14, 0.68)	0.31 (0.12, 0.80)	0.915	0.52 (0.27, 0.98)	0.77 (0.24, 1.38)	0.556
ALB (g/dL)	3.74 (3.34, 4.15)	3.72 (3.30, 4.15)	0.897	3.85 (3.22, 4.13)	3.60 (3.24, 4.07)	0.427	3.36 (2.86, 3.83)	3.35 (2.86, 3.78)	0.824
**Crohn's disease**									
Perianal abscess			0.779			0.987			0.735
No	155.3 (64.3)	284.8 (63.0)		75.5 (73.4)	127.6 (73.5)		28.2 (62.3)	41.6 (66.0)	
Yes	86.3 (35.7)	166.9 (37.0)		27.4 (26.6)	46.0 (26.5)		17.1 (37.7)	21.4 (34.0)	
Montreal A			0.973			0.14			0.819
A1	36.2 (15.0)	66.4 (14.7)		17.4 (16.9)	12.8 (7.4)		5.3 (11.7)	5.1 (8.0)	
A2	179.2 (74.2)	338.6 (75.0)		69.1 (67.2)	109.7 (63.2)		34.7 (76.7)	50.3 (79.9)	

A3	26.3 (10.9)	46.7 (10.3)		16.4 (15.9)	51.0 (29.4)		5.2 (11.5)	7.6 (12.1)	
Montreal B			0.974			0.962			0.816
B1	137.3 (56.8)	260.9 (57.7)		24.1 (23.4)	38.1 (21.9)		5.8 (12.8)	5.4 (8.5)	
B2	49.0 (20.3)	90.4 (20.0)		55.5 (53.9)	93.0 (53.6)		29.0 (64.1)	41.2 (65.4)	
B3	55.4 (22.9)	100.5 (22.3)		23.3 (22.7)	42.6 (24.5)		10.5 (23.1)	16.5 (26.1)	
Montreal L			0.991			0.666			0.829
L1	62.8 (26.0)	114.4 (25.3)		24.5 (23.8)	40.3 (23.2)		14.9 (33.0)	19.7 (31.3)	
L2	13.2 (5.5)	23.9 (5.3)		11.2 (10.9)	11.1 (6.4)		1.7 (3.7)	5.3 (8.4)	
L3	162.4 (67.2)	306.2 (67.8)		67.2 (65.3)	121.5 (70.0)		28.0 (61.9)	37.0 (58.8)	
L4	3.2 (1.3)	7.2 (1.6)		0.0 (0.0)	0.6 (0.3)		0.7 (1.5)	0.9 (1.5)	
CDAI			0.894			0.806			0.986
Remission	37.8 (15.7)	64.4 (14.3)		10.0 (9.7)	14.8 (8.5)		1.8 (3.9)	2.9 (4.5)	
Mild active	115.7 (47.9)	230.3 (51.0)		46.4 (45.1)	68.3 (39.4)		13.7 (30.4)	20.5 (32.5)	
Moderate active	80.4 (33.2)	141.3 (31.3)		42.4 (41.2)	81.3 (46.8)		24.9 (55.1)	34.3 (54.5)	
Severe active	7.8 (3.2)	15.7 (3.5)		4.2 (4.0)	9.1 (5.3)		4.8 (10.6)	5.3 (8.5)	
**Extrintestinal**									
Extrintestinal			0.86			0.069			0.685
No	195.0 (80.7)	367.7 (81.4)		89.0 (86.5)	122.6 (70.6)		37.0 (81.7)	49.4 (78.5)	
Yes	46.6 (19.3)	84.1 (18.6)		13.9 (13.5)	51.0 (29.4)		8.3 (18.3)	13.6 (21.5)	
Oral ulcer			0.722			0.365			0.785
No	218.2 (90.3)	402.9 (89.2)		97.7 (95.0)	158.2 (91.1)		41.5 (91.7)	56.7 (90.0)	
Yes	23.5 (9.7)	48.9 (10.8)		5.2 (5.0)	15.4 (8.9)		3.7 (8.3)	6.3 (10.0)	
Liver/gallbladder			0.908			0.4			0.89
No	239.4 (99.1)	447.1 (99.0)		100.0 (97.2)	162.7 (93.7)		43.6 (96.3)	61.0 (96.8)	
Yes	2.2 (0.9)	4.6 (1.0)		2.8 (2.8)	10.9 (6.3)		1.7 (3.7)	2.0 (3.2)	
Skin			0.492			0.823			0.754
No	230.2 (95.2)	436.9 (96.7)		101.4 (98.6)	170.6 (98.3)		43.2 (95.5)	59.3 (94.2)	
Yes	11.5 (4.8)	14.9 (3.3)		1.4 (1.4)	3.0 (1.7)		2.0 (4.5)	3.6 (5.8)	
Eye			0.998			0.858			0.995
No	239.5 (99.1)	447.7 (99.1)		101.0 (98.1)	170.9 (98.5)		44.6 (98.5)	62.0 (98.5)	
Yes	2.2 (0.9)	4.1 (0.9)		1.9 (1.9)	2.7 (1.5)		0.7 (1.5)	1.0 (1.5)	
Osteoarthritis			0.862			0.521			0.425
No	232.5 (96.2)	436.0 (96.5)		100.4 (97.6)	171.1 (98.6)		44.4 (98.2)	60.3 (95.8)	
Yes	9.1 (3.8)	15.7 (3.5)		2.5 (2.4)	2.4 (1.4)		0.8 (1.8)	2.7 (4.2)	

Supplementary Table 17. Baseline characteristics of patients with and without biologic therapy across gradient boosting machine (GBM)-defined low-, intermediate-, and high-risk strata before inverse probability of treatment weighting in Imputed dataset 5

	Low-risk			Intermediate-risk			High-risk		
	No-Biologics	Biologics	*P*	No-Biologics	Biologics	*P*	No-Biologics	Biologics	*P*
	n =241	n =447		n =105	n =154		n =47	n =66	
**Demographic data**									
Gender (n, %)			0.063			0.134			1
Male	191 (79.3)	324 (72.5)		82 (78.1)	106 (68.8)		35 (74.5)	48 (72.7)	
Female	50 (20.7)	123 (27.5)		23 (21.9)	48 (31.2)		12 (25.5)	18 (27.3)	
Smoking (n, %)			<0.001			0.054			0.326
No	179 (74.3)	385 (86.1)		72 (68.6)	123 (79.9)		30 (63.8)	49 (74.2)	
Yes	62 (25.7)	62 (13.9)		33 (31.4)	31 (20.1)		17 (36.2)	17 (25.8)	
BMI (kg/m^2)	19.10 (17.36, 21.88)	19.03 (17.30, 21.22)	0.116	17.71 (16.16, 20.24)	17.30 (16.14, 19.43)	0.165	17.92 (16.19, 19.05)	17.30 (15.96, 20.04)	0.574
**Laboratory data**									
WBC (10^9/L)	6.26 (5.12, 8.07)	6.46 (5.22, 8.07)	0.448	6.50 (4.62, 9.70)	6.26 (4.71, 8.08)	0.44	6.44 (4.68, 7.58)	6.42 (4.48, 9.43)	0.616
Hb (g/dL)	12.30 (10.70, 13.80)	12.20 (10.80, 13.70)	0.57	11.90 (9.90, 13.30)	11.90 (10.10, 13.60)	0.708	11.40 (9.00, 12.80)	10.30 (8.65, 11.90)	0.094
ESR (mm/h)	15.00 (7.00, 34.00)	20.00 (10.00, 40.00)	0.001	24.00 (6.00, 40.00)	25.50 (8.00, 48.50)	0.224	41.00 (17.50, 51.00)	41.00 (19.25, 59.00)	0.341
CRP (mg/dL)	1.08 (0.34, 2.32)	1.16 (0.40, 2.46)	0.313	2.48 (0.47, 6.10)	1.73 (0.39, 6.47)	0.403	6.48 (1.73, 9.13)	7.85 (2.45, 9.97)	0.208
TT (s)	16.40 (15.80, 17.20)	16.50 (15.80, 17.20)	0.855	16.50 (15.80, 17.10)	16.80 (16.00, 17.20)	0.089	16.90 (16.20, 17.80)	16.10 (15.12, 17.70)	0.015
APTT (s)	29.60 (27.90, 33.50)	29.80 (27.80, 32.50)	0.963	30.50 (28.00, 36.10)	29.90 (27.52, 33.12)	0.33	30.20 (28.90, 39.70)	30.40 (28.72, 35.67)	0.556
PT (s)	11.60 (11.00, 12.40)	11.70 (11.10, 12.80)	0.115	12.30 (11.30, 13.30)	12.20 (11.40, 13.30)	0.832	12.70 (11.95, 13.60)	12.75 (11.83, 13.90)	0.56
Fg (g/L)	3.71 (3.10, 4.31)	3.80 (3.03, 4.54)	0.436	3.34 (2.41, 4.89)	3.20 (2.40, 4.78)	0.646	3.93 (2.38, 4.65)	3.40 (2.76, 4.86)	0.977
DD (mg/L)	0.33 (0.21, 0.57)	0.31 (0.17, 0.54)	0.031	0.35 (0.18, 0.79)	0.36 (0.22, 0.85)	0.755	0.44 (0.26, 0.98)	0.86 (0.35, 1.61)	0.039
ALB (g/dL)	3.77 (3.35, 4.19)	3.77 (3.31, 4.16)	0.986	3.67 (3.22, 4.11)	3.70 (3.20, 4.19)	0.718	3.58 (3.02, 3.89)	3.24 (2.82, 3.71)	0.066
**Crohn's disease**									
Perianal abscess			0.004			0.001			0.311
No	168 (69.7)	261 (58.4)		86 (81.9)	95 (61.7)		35 (74.5)	42 (63.6)	
Yes	73 (30.3)	186 (41.6)		19 (18.1)	59 (38.3)		12 (25.5)	24 (36.4)	
Montreal A			<0.001			0.01			0.703
1	20 (8.3)	83 (18.6)		5 (4.8)	16 (10.4)		4 (8.5)	5 (7.6)	
2	182 (75.5)	326 (72.9)		74 (70.5)	120 (77.9)		34 (72.3)	52 (78.8)	

3	39 (16.2)	38 (8.5)		26 (24.8)	18 (11.7)		9 (19.1)	9 (13.6)	
Montreal B			0.008			0.006			0.314
1	121 (50.2)	278 (62.2)		16 (15.2)	50 (32.5)		3 (6.4)	5 (7.6)	
2	60 (24.9)	79 (17.7)		57 (54.3)	72 (46.8)		36 (76.6)	42 (63.6)	
3	60 (24.9)	90 (20.1)		32 (30.5)	32 (20.8)		8 (17.0)	19 (28.8)	
Montreal L			0.042			0.27			0.145
1	73 (30.3)	95 (21.3)		33 (31.4)	33 (21.4)		18 (38.3)	16 (24.2)	
2	14 (5.8)	24 (5.4)		8 (7.6)	12 (7.8)		1 (2.1)	8 (12.1)	
3	149 (61.8)	322 (72.0)		64 (61.0)	108 (70.1)		27 (57.4)	41 (62.1)	
4	5 (2.1)	6 (1.3)		0 (0.0)	1 (0.6)		1 (2.1)	1 (1.5)	
CDAI			0.104			0.851			0.735
0	38 (15.8)	63 (14.1)		12 (11.4)	16 (10.4)		1 (2.1)	3 (4.5)	
1	131 (54.4)	210 (47.0)		40 (38.1)	63 (40.9)		14 (29.8)	24 (36.4)	
2	63 (26.1)	158 (35.3)		47 (44.8)	63 (40.9)		27 (57.4)	34 (51.5)	
3	9 (3.7)	16 (3.6)		6 (5.7)	12 (7.8)		5 (10.6)	5 (7.6)	
**Extrintestinal**									
Extrintestinal			0.91			0.921			0.887
No	200 (83.0)	368 (82.3)		88 (83.8)	131 (85.1)		37 (78.7)	50 (75.8)	
Yes	41 (17.0)	79 (17.7)		17 (16.2)	23 (14.9)		10 (21.3)	16 (24.2)	
Oral ulcer			0.724			0.12			0.489
No	219 (90.9)	401 (89.7)		101 (96.2)	139 (90.3)		44 (93.6)	58 (87.9)	
Yes	22 (9.1)	46 (10.3)		4 (3.8)	15 (9.7)		3 (6.4)	8 (12.1)	
Liver/gallbladder			1			0.369			0.696
No	239 (99.2)	442 (98.9)		101 (96.2)	152 (98.7)		44 (93.6)	64 (97.0)	
Yes	2 (0.8)	5 (1.1)		4 (3.8)	2 (1.3)		3 (6.4)	2 (3.0)	
Skin			0.947			1			1
No	233 (96.7)	434 (97.1)		103 (98.1)	150 (97.4)		44 (93.6)	62 (93.9)	
Yes	8 (3.3)	13 (2.9)		2 (1.9)	4 (2.6)		3 (6.4)	4 (6.1)	
Eye			0.229			1			1
No	237 (98.3)	445 (99.6)		103 (98.1)	152 (98.7)		46 (97.9)	65 (98.5)	
Yes	4 (1.7)	2 (0.4)		2 (1.9)	2 (1.3)		1 (2.1)	1 (1.5)	
Osteoarthritis			1			0.195			0.591
No	232 (96.3)	430 (96.2)		100 (95.2)	152 (98.7)		46 (97.9)	62 (93.9)	
Yes	9 (3.7)	17 (3.8)		5 (4.8)	2 (1.3)		1 (2.1)	4 (6.1)	

Supplementary Table 18. Baseline characteristics of patients with and without biologic therapy across gradient boosting machine (GBM)-defined low-, intermediate-, and high-risk strata after inverse probability of treatment weighting in Imputed dataset 5

	Low-risk			Intermediate-risk			High-risk		
	No-Biologics	Biologics	*P*	No-Biologics	Biologics	*P*	No-Biologics	Biologics	*P*
	n =240.4	n =446.9		n =102.4	n =167.5		n =47.4	n =63.6	
**Demographic data**									
Gender (n, %)									
Male	180.3 (75.0)	336.9 (75.4)	0.921	79.5 (77.6)	128.3 (76.6)	0.881	38.8 (81.8)	48.3 (75.9)	0.456
Female	60.1 (25.0)	110.0 (24.6)		22.9 (22.4)	39.2 (23.4)		8.6 (18.2)	15.3 (24.1)	
Smoking (n, %)									
No	194.1 (80.7)	363.8 (81.4)	0.84	76.4 (74.6)	110.0 (65.7)	0.299	34.5 (72.7)	44.8 (70.5)	0.827
Yes	46.3 (19.3)	83.1 (18.6)		26.0 (25.4)	57.5 (34.3)		12.9 (27.3)	18.7 (29.5)	
BMI (kg/m^2)	18.82 (17.21, 21.45)	19.10 (17.30, 21.26)	0.738	17.25 (15.87, 19.77)	17.58 (16.36, 20.05)	0.358	16.75 (15.90, 18.38)	17.28 (15.94, 19.72)	0.779
**Laboratory data**									
WBC(10^9/L)	6.38 (5.12, 8.29)	6.43 (5.19, 8.06)	0.834	6.93 (4.62, 9.14)	6.41 (4.82, 8.35)	0.986	6.76 (4.97, 7.56)	5.67 (4.35, 9.38)	0.491
Hb (g/dL)	12.30 (10.50, 13.61)	12.20 (10.80, 13.80)	0.984	12.10 (10.30, 13.30)	11.81 (9.31, 13.60)	0.507	10.80 (8.31, 12.32)	10.47 (9.16, 11.72)	0.917
ESR (mm/h)	16.00 (7.00, 38.00)	18.00 (9.00, 37.61)	0.36	25.29 (7.29, 43.24)	26.00 (8.74, 50.51)	0.365	40.47 (31.00, 49.23)	36.00 (14.00, 56.99)	0.913
CRP (mg/dL)	0.93 (0.34, 2.13)	1.16 (0.37, 2.40)	0.287	1.69 (0.47, 6.10)	1.73 (0.52, 7.06)	0.72	7.21 (2.67, 9.13)	7.34 (1.52, 9.34)	0.683
TT (s)	16.50 (15.90, 17.30)	16.50 (15.80, 17.20)	0.437	16.70 (15.90, 17.00)	16.70 (15.63, 17.26)	0.818	16.68 (16.18, 17.30)	16.69 (15.34, 18.10)	0.668
APTT (s)	29.60 (27.90, 33.92)	29.90 (27.80, 32.64)	0.979	29.00 (27.19, 33.18)	30.22 (27.70, 32.83)	0.342	29.62 (28.54, 38.50)	30.45 (28.80, 35.04)	0.838
PT (s)	11.70 (11.00, 12.70)	11.60 (11.00, 12.70)	0.976	11.84 (11.11, 12.90)	12.18 (11.40, 12.88)	0.234	12.67 (11.85, 13.60)	12.40 (11.52, 13.70)	0.741
Fg (g/L)	3.79 (3.08, 4.34)	3.77 (3.02, 4.34)	0.941	3.58 (2.50, 4.66)	3.51 (2.41, 4.80)	0.78	3.98 (2.98, 4.65)	3.31 (2.35, 4.85)	0.609
DD (mg/L)	0.32 (0.22, 0.57)	0.31 (0.17, 0.54)	0.047	0.33 (0.13, 0.68)	0.37 (0.18, 0.81)	0.556	0.44 (0.26, 0.97)	0.75 (0.23, 1.32)	0.373
ALB (g/dL)	3.77 (3.39, 4.19)	3.77 (3.31, 4.15)	0.878	3.68 (3.14, 4.13)	3.61 (2.99, 4.09)	0.513	3.38 (2.88, 3.82)	3.35 (2.90, 3.78)	0.817
**Crohn's disease**									
Perianal abscess			0.654			0.861			0.804
No	155.1 (64.5)	279.9 (62.6)		72.7 (71.0)	121.6 (72.6)		30.3 (63.8)	42.4 (66.6)	
Yes	85.3 (35.5)	167.1 (37.4)		29.7 (29.0)	45.9 (27.4)		17.2 (36.2)	21.2 (33.4)	
Montreal A			0.91			0.195			0.7
1	32.9 (13.7)	66.5 (14.9)		18.0 (17.6)	12.7 (7.6)		6.2 (13.0)	4.8 (7.6)	
2	181.5 (75.5)	334.9 (74.9)		67.4 (65.8)	110.2 (65.8)		35.7 (75.2)	50.5 (79.4)	

3	26.0 (10.8)	45.5 (10.2)		17.0 (16.6)	44.6 (26.6)		5.6 (11.7)	8.2 (12.9)	
Montreal B			0.941			0.829			0.844
1	134.8 (56.1)	257.1 (57.5)		21.6 (21.1)	38.0 (22.7)		5.2 (10.9)	5.1 (8.0)	
2	50.4 (21.0)	89.5 (20.0)		57.9 (56.5)	86.2 (51.5)		31.8 (67.2)	41.6 (65.4)	
3	55.2 (22.9)	100.3 (22.4)		22.9 (22.4)	43.2 (25.8)		10.4 (21.9)	16.9 (26.6)	
Montreal L			0.982			0.939			0.996
1	63.7 (26.5)	113.8 (25.5)		24.3 (23.8)	38.7 (23.1)		14.6 (30.8)	20.8 (32.7)	
2	13.7 (5.7)	24.0 (5.4)		9.5 (9.3)	14.3 (8.5)		4.1 (8.7)	5.4 (8.5)	
3	159.7 (66.4)	302.3 (67.6)		68.5 (66.9)	113.8 (68.0)		28.0 (59.0)	36.4 (57.3)	
4	3.2 (1.3)	6.9 (1.5)		0.0 (0.0)	0.6 (0.4)		0.7 (1.5)	1.0 (1.6)	
CDAI			0.985			0.923			0.968
0	36.9 (15.4)	64.3 (14.4)		9.5 (9.3)	15.0 (9.0)		1.2 (2.6)	2.4 (3.8)	
1	117.7 (49.0)	225.0 (50.4)		46.4 (45.3)	69.5 (41.5)		17.6 (37.2)	20.9 (33.0)	
2	77.4 (32.2)	141.6 (31.7)		41.9 (40.9)	73.7 (44.0)		24.3 (51.2)	34.7 (54.6)	
3	8.3 (3.5)	16.0 (3.6)		4.5 (4.4)	9.3 (5.5)		4.3 (9.0)	5.5 (8.7)	
**Extrintestinal**									
Extrintestinal			0.905			0.132			0.58
No	197.5 (82.2)	369.0 (82.6)		88.6 (86.5)	125.5 (74.9)		39.3 (82.9)	49.9 (78.6)	
Yes	42.9 (17.8)	77.9 (17.4)		13.8 (13.5)	42.0 (25.1)		8.1 (17.1)	13.6 (21.4)	
Oral ulcer			0.758			0.436			0.715
No	218.7 (91.0)	403.1 (90.2)		97.0 (94.7)	153.3 (91.5)		43.8 (92.4)	57.4 (90.3)	
Yes	21.7 (9.0)	43.9 (9.8)		5.4 (5.3)	14.2 (8.5)		3.6 (7.6)	6.2 (9.7)	
Liver/gallbladder			0.993			0.471			0.954
No	237.9 (99.0)	442.3 (99.0)		99.4 (97.1)	157.5 (94.1)		45.7 (96.4)	61.1 (96.2)	
Yes	2.5 (1.0)	4.6 (1.0)		3.0 (2.9)	10.0 (5.9)		1.7 (3.6)	2.4 (3.8)	
Skin			0.713			0.726			0.742
No	231.2 (96.2)	432.7 (96.8)		101.0 (98.7)	164.5 (98.2)		45.5 (95.8)	60.2 (94.6)	
Yes	9.2 (3.8)	14.2 (3.2)		1.3 (1.3)	3.0 (1.8)		2.0 (4.2)	3.4 (5.4)	
Eye			0.888			0.934			0.974
No	238.3 (99.1)	443.5 (99.2)		100.9 (98.5)	165.2 (98.6)		46.8 (98.6)	62.6 (98.5)	
Yes	2.1 (0.9)	3.5 (0.8)		1.5 (1.5)	2.3 (1.4)		0.7 (1.4)	0.9 (1.5)	
Osteoarthritis			0.896			0.481			0.406
No	231.4 (96.2)	431.1 (96.5)		99.9 (97.5)	165.2 (98.6)		46.6 (98.3)	60.9 (95.8)	
Yes	9.1 (3.8)	15.9 (3.5)		2.5 (2.5)	2.3 (1.4)		0.8 (1.7)	2.7 (4.2)	
